# *Streptomyces coelicolor* strains lacking polyprenol phosphate mannose synthase and protein O-mannosyl transferase are hyper-susceptible to multiple antibiotics

**DOI:** 10.1099/mic.0.000605

**Published:** 2018-02-01

**Authors:** Robert Howlett, Nicholas Read, Anpu Varghese, Charles Kershaw, Y. Hancock, Margaret C. M. Smith

**Affiliations:** ^1^​ Department of Biology, University of York, York, UK; ^2^​ Institute of Medical Sciences, University of Aberdeen, Aberdeen, UK; ^3^​ Department of Physics, University of York, York, UK; ^4^​ York Centre for Complex Systems Analysis, University of York, York, UK

**Keywords:** protein, O-glycosylation, bacteriophage, receptor, RNAseq, Raman spectroscopy, ECF sigma factor, cell wall biogenesis

## Abstract

Polyprenol phosphate mannose (PPM) is a lipid-linked sugar donor used by extra-cytoplasmic glycosyl tranferases in bacteria. PPM is synthesiszed by polyprenol phosphate mannose synthase, Ppm1, and in most Actinobacteria is used as the sugar donor for protein O-mannosyl transferase, Pmt, in protein glycosylation. Ppm1 and Pmt have homologues in yeasts and humans, where they are required for protein O-mannosylation. Actinobacteria also use PPM for lipoglycan biosynthesis. Here we show that *ppm1* mutants of *Streptomyces coelicolor* have increased susceptibility to a number of antibiotics that target cell wall biosynthesis. The *pmt* mutants also have mildly increased antibiotic susceptibilities, in particular to β-lactams and vancomycin. Despite normal induction of the vancomycin gene cluster, *vanSRJKHAX*, the *pmt* and *ppm1* mutants remained highly vancomycin sensitive indicating that the mechanism of resistance is blocked post-transcriptionally. Differential RNA expression analysis indicated that catabolic pathways were downregulated and anabolic ones upregulated in the *ppm1* mutant compared to the parent or complemented strains. Of note was the increase in expression of fatty acid biosynthetic genes in the *ppm1^-^* mutant. A change in lipid composition was confirmed using Raman spectroscopy, which showed that the *ppm1*^-^ mutant had a greater relative proportion of unsaturated fatty acids compared to the parent or the complemented mutant. Taken together, these data suggest that an inability to synthesize PPM (*ppm1*) and loss of the glycoproteome (*pmt^-^* mutant) can detrimentally affect membrane or cell envelope functions leading to loss of intrinsic and, in the case of vancomycin, acquired antibiotic resistance.

## Introduction

**Fig. 1.**
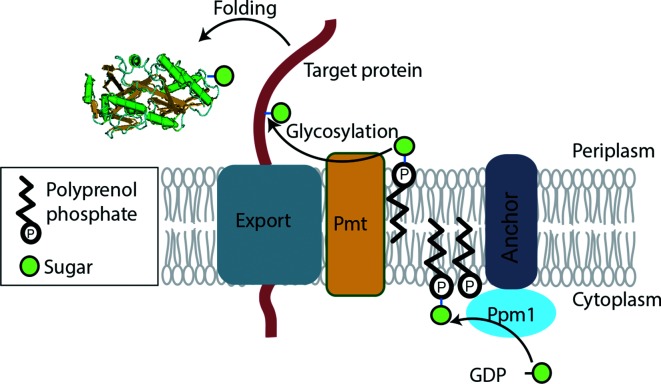
The protein O-glycosylation pathway in *S. coelicolor*. Two enzyme activities, Ppm1 and Pmt (encoded by *sco1423* and *sco3154*, respectively), required for glycosylation of the periplasmic phosphate-binding protein, PstS, are membrane bound [9]. *In vitro* Ppm1 transfers mannose from GDP-mannose to a nonaprenol to generate polyprenol phosphate mannose, which then acts as the sugar carrier for Pmt to glycosylate peptides containing a glycosylation site [9]. In *Mycobacterium tuberculosis* protein, O-glycosylation was associated with the secretion apparatus [13].

In the Actinobacteria, mannose is an important component of extracellular glycoconjugates including lipoglycans and glycoproteins [1, 2]. Lipomannan (LM) and lipoarabinomannan (LAM) are essential constituents of the cell envelope in mycobacteria [3], and mutants of corynebacteria that lack these molecules grow poorly [4]. Membrane and secreted proteins are also modified by mannose residues in the Actinobacteria and species of *Mycobacterium*, *Corynebacterium* and *Streptomyces* have been shown to contain glycoproteins [5–9]. Extra-cytoplasmic glycosyl transferases require a lipid-linked sugar donor, which for the transfer of mannose is polyprenol phosphate mannose (PPM). The enzyme polyprenol phosphate mannose synthase, Ppm1, synthesizes PPM by catalysing the transfer of mannose from GDP-mannose to polyprenol phosphate on the cytoplasmic face of the plasma membrane and PPM is then flipped in the membrane, transporting the mannose moiety to the periplasm [10] (Fig. 1). In mycobacteria, Ppm1 is an essential enzyme as it is required for lipomannan biosynthesis [11], and *Corynebacterium ppm1* mutants have a reduced growth rate [12].

We are interested in the role of protein O-glycosylation in bacteria and ultimately we aim to understand how the glycans affect protein function. The protein O-glycosylation pathway in the Actinobacteria is highly reminiscent of the O-mannosylation pathway in yeasts and humans [2, 9, 13]. In fungi, mutations that inactivate PMTs lead to loss of fitness and/or avirulent phenotypes [14–19]. In humans, loss of PoMT leads to a developmental phenotype [20]. Overall the evidence suggests that protein O-mannosylation is a fundamentally important protein modification in prokaryotes and eukaryotes.

*S. coelicolor* is a convenient model to study the protein O-glycosylation pathway in bacteria, as the *pmt* and *ppm1* genes are not essential [21, 22] and *Streptomyces* spp. are not known to generate the heavily glycosylated lipomannans and lipoarabinomannans that require Ppm1 for synthesis. In previous work we showed that *S. coelicolor* Ppm1 (SCO1423) catalyses the transfer of mannose from GDP-mannose to polyprenol phosphate to generate PPM, and Pmt (SCO3154) was shown to transfer mannose from PPM to target peptides [9] (Fig. 1). Ppm1 and Pmt activities are confined to the membrane fractions. The periplasmic phosphate-binding protein, PstS, part of a high-affinity phosphate uptake system, was shown to be *O*-glycosylated with a trihexose [9]. The enzymes Pmt and Ppm1 were first identified as being required for phage ϕC31 infection, suggesting that the phage receptor is a glycoprotein(s) [21, 22].

Here we report on the global phenotypes of mutations that lead to an inability to synthesize PPM and the glycoproteome in *S. coelicolor*. Mutations in *pmt* and *ppm1* confer pleiotropic phenotypes including increased susceptibilities to multiple antibiotics, most of which act at different stages of cell wall biogenesis. Global transcriptional analysis in the *ppm1*^–^ mutant compared to the parent (*ppm1^+^*) strain showed that this mutation conferred a general switch from catabolism to anabolism and a major change in fatty acid metabolism. Changes in lipid composition in the *ppm1^-^* mutant were confirmed using Raman spectroscopy. We propose that these global effects on the cell envelope affect periplasmic and membrane protein function, leading to antibiotic susceptibility.

## Methods

### Bacterial strains

*Streptomyces coelicolor* strains with mutations in *ppm1* (DT3017; *ppm1E218V*, DT1020; *ppm1H116D* and DT1029; *ppm1S163L*) and *pmt* (DT1025; frameshift from A121, and DT2008; mutation uncharacterized) were obtained previously [21, 22]. *E. coli* strain DH5α was used for propagation of plasmids pDT10 and pDT16, also published previously [21, 22]. The DNA methylation-deficient strain of *E. coli*, ET12567 (pUZ8002), was used as the donor host for plasmid conjugation to *Streptomyces* species as described previously [23, 24]. *Streptomyces* strains were routinely maintained on soya mannitol (SM) agar at 30 °C [24] and spore stocks maintained at −38 °C.

### Disc diffusion assays

Difco nutrient (DN) agar plates were evenly spread with approximately 1×10^7^ *Streptomyces* spores. Sterile filter discs (5 mm width) were placed on the surface of the inoculated agar plates and 10 µl of an antibiotic stock solution was allowed to absorb to the disc. Plates were incubated at 30 °C for 2 days and the zones of inhibition were measured. At least three biological replicates and at least two technical replicates were used for each strain.

### Quantitative mRNA analysis and culture conditions

Samples were prepared for quantitative reverse transcriptase-polymerase chain reaction (qRT-PCR) analysis by inoculation of 500 ml DN broth in a 2 l baffled flask to a starting OD_450_ of 0.05 with heat-shocked pre-germinated spores. Pre-germination was performed according to Kieser *et al.* [24]; approximately 10^9^ c.f.u. *S. coelicolor* spores were germinated for 10 h in 100 ml germination medium in 1 l baffled flasks at 30 °C. Cultures were then grown at 30 °C for 6 h at 250 r.p.m. before the addition of vancomycin to a final concentration of 0.1 µg ml^−1^. Next, 43 ml of culture was sampled and added to 7 ml stop solution (5 % phenol in ethanol, stored at –20 °C) at time-points 0, 30, 60 and 90 min. RNA was extracted from samples as described below and cDNA was created using SuperScript III reverse transcriptase (Life technologies) using the manufacturer's instructions with the following altered temperature cycle: 25 °C, 10 min; 42 °C, 120 min; 50 °C, 30 min; 55 °C, 30 min; 85 °C, 5 min. Primers used for qRT-PCR were designed using Primer3 software [25]. Reactions were carried out on a StepOne PCR machine (Applied Biosystems) with each 20 µl qRT-PCR reaction containing 10 µl Fast SYBR Green Mast Mix (Applied Biosystems) 25 ng cDNA and optimized primer and DMSO concentrations. Optimized conditions were as follows: primers RH111 and RH112 300 nM no DMSO; RH107 and RH108 500 nM with 4.8 % DMSO; RH109 and RH110 200 nM with 4.8 % DMSO (Table S1, available in the online version of this article). The principal sigma factor of *S. coelicolor* (*hrdB*) was used as a representative constitutively expressed, housekeeping gene.

### Fermentor cultivation conditions

Cultivations for RNAseq analysis were performed in 7 l fermentors (Applikon) containing 2.5 l of DN broth and 2 ml antifoam C emulsion (Sigma-Aldrich), with a starting pH adjusted to 7 with NaOH. Temperature was maintained at 30 °C and pH maintained at 7 through the automatic addition of 2 M HCl. Dissolved oxygen was maintained at a minimum of 30 % by automatic adjustment of the agitation speed, with a minimum and maximum rate of 300 and 1000 ,respectively, and a constant aeration pressure of 2.5–3.5 bar. For the inoculum approximately 10^9^ c.f.u*. S. coelicolor* spores were germinated for 10 h in 100 ml germination medium in 1 l baffled flasks at 30 °C following heat shock as described in Kieser *et al.* [24], before being used to inoculate equilibrated fermentor culture medium to an OD_450_ of 0.05. Growth was measured using dry weight while dissolved oxygen, pH, agitation speed and temperature were logged online throughout.

### Isolation of RNA and transcriptome analysis

RNA for RNAseq analysis was isolated from fermentor-grown cultures as described above. The equivalent of 40 OD_600_ units were sampled and immediately added to 1/8 volume stop solution (5 % phenol in ethanol, stored at –20 °C). Cultures were spun down at 12 000 r.p.m., 4 °C for 5 min before pellets were flash-frozen in liquid nitrogen and stored at −80 °C for future processing. RNA isolation was performed using a modified version of the Kirby mix protocol as follows [24]: 14 g of glass beads were added to defrosted pellets along with 5 ml of 30 °C modified Kirby mix before being vigorously vortexed for 2 min. Acid phenol:chloroform 1 : 1 was then used to isolate nucleic acids before being precipitated with 4 M acetate and 100 % isopropanol. Nucleic acids were subject to an ethanol wash before final resuspension in water. Nucleic acids were repeatedly treated with DNase I using conditions described by the vendor (Roche) until DNA could no longer be amplified using polymerase chain reaction (PCR). RNA was then enriched for mRNA using *MICROBE*xpress Bacteria beads, as described by the manufacturer (Ambion).

The use of stop solution to prevent artefacts due to variation in harvesting of samples was validated as follows: two samples were collected from the fermentor (containing J1929, time point 24 h) and stop solution was added to both. RNA was prepared immediately for one sample whereas the other was incubated on ice for 1 h prior to preparation of RNA. The RNA sequences of both samples was analysed for significant changes that might have resulted from RNA degradation. Subsequent linear regression analysis gave an *R*^2^ of 0.959, indicating that the use of the stop solution was a valid procedure for sample preparation (Fig. S2).

Generation of cDNA and production of Illumina HiSeq data were performed by Vertis Biotechnologie AG (Germany). In brief, mRNAs were fragmented with ultrasound before Antarctic phosphatase treatment and re-phosphorylation with polynucleotide kinase. RNA fragments were then poly(A)-tailed using poly(A) polymerase, and an RNA adapter was ligated to mRNA 5′ phosphate. First-strand cDNA synthesis was performed using an oligo(dT)-adapter primer and M-MLV reverse transcriptase before PCR amplification. The cDNA samples were pooled and size fractionated in the range 250–500 bp and run on an Illumina HiSeq 2000 system with a 100 bp read length.

Sequences were trimmed using Sickle and aligned to the *S. coelicolor* genome (NCBI, accession number NC_003888) using BWA MEM (http://bio-bwa.sourceforge.net). Normalized counts and differential gene expression analysis were performed with DESeq2 in RIBioconductor [26]. Gene ontology analysis was performed using GOEAST [27], and read counts were visualized using the Integrated Genomics Viewer [28, 29]. RNA sequencing data is available at the GEO database accession number GSE107982.

### Raman spectroscopy sample preparation

*S. coelicolor* strains J1929, DT3017 (*ppm1*^–^ mutant) and DT3017:pDT16 (complemented mutant) were prepared for Raman spectroscopy by subjecting spores (approximately 10^9^ c.f.u. in 10 ml TES 0.5 M, pH8) to heat shock (50 °C, 10 mins) and then adding to 10 ml double strength germination medium in a 250 ml baffled conical flask [24]. After incubation (37 °C, 180 r.p.m. for 8–9 h) and centrifugation to pellet cells, the pellet was resuspended in 40 ml DN broth to an OD_450_ of 0.05 and incubation continued for a further 12–13 h. Ten millilitres was removed for dry weight measurement and the remaining culture was centrifuged, the cell pellet washed once in 30 ml high-purity deionized water and then plated via pipette-deposit directly onto small CaF_2_ disks (13 mm diameter and 1 mm thickness). The bacteria were then gently spread over the disk surface, with excess water removed by pipette aspiration and then air-dried in a fume cupboard in preparation for Raman spectroscopy.

### Raman spectroscopy experiments and analyses

Raman spectroscopy point spectra were collected from randomly selected cell populations of *S. coelicolor* J1929 (parent), DT3017 (*ppm1^-^* mutant) and DT3017:pDT16 (complemented mutant) using an HORIBA XploRA micro-Raman instrument with 532 nm laser, ×100 objective (0.9 NA) and 2400 lines/mm spectral grating. Measurements were made in confocal mode using a laser spot size of ~1 µm, with 3.5 mW laser power and 1 cm^−1^ spectral resolution.

Spectra were collected using the HORIBA LabSpec 6 software in the 600–1800 cm^−1^ cell-fingerprint and 2750–3110 cm^−1^ high-wavenumber ranges. Each spectrum was obtained using 90s acquisition time averaged over two spectral repetitions, with 40–55 spectra randomly collected across each cell line over three experimental repeats. The total number of spectra was averaged to obtain a single representative spectrum for each strain.

The averaged spectra were baseline-corrected from the first to the last spectral point in the fingerprint and high-wavenumber ranges using the ‘*Raman tool set*’ package [30]. Peak regions in the acquired spectral ranges were then linear baseline-corrected and Gaussian peak-fitted using IGOR Pro 6.35 to obtain the peak properties (peak positions, peak intensities, etc.). Biomolecular peak assignments were made by correlating the fitted peak positions to literature references [31, 32]. The total unsaturated fatty acid (TUFA)/total fatty acid (TFA) peak intensity ratio for each cell line was determined with uncertainties calculated from the propagated standard error of the mean in the measured TUFA and TFA peak intensity values. The percentage standard error of the mean for the TUFA/TFA ratio was converged as a function of the number of spectra in each spectral average, ensuring that sufficient spectra were collected for accurate representation of each sample population.

## Results

### Mutations in *ppm1* and *pmt* in *S. coelicolor* confer hypersensitivity to cell wall-acting antibiotics

**Fig. 2.**
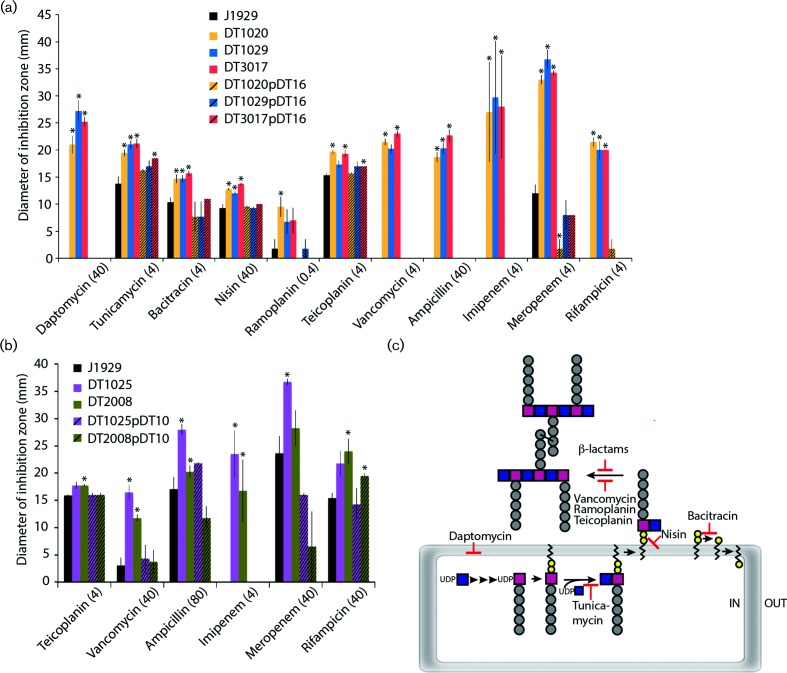
*Streptomyces coelicolor* strains defective in protein glycosylation genes are hypersensitive to multiple antibiotics. Shown are diameters of growth inhibition zones from disc diffusion assays for the *ppm1*^–^ mutants, DT1020, DT1029 and DT3017 against the parent strain J1929 and the complemented strains DT2010:pDT16, DT1029:pDT16 and DT3017:pDT16 (a), and the *pmt*^–^ mutants, DT1025 and DT2008, the parent strain J1929 and the complemented strains DT1025:pDT10 and DT2008:pDT10 (b). Shown are the averages of 4 replicates with SEM. *Indicates a *P*<0.05 that the observed difference between the mutant strains versus J1929 has occurred by chance. The concentration of antibiotic used is shown in parentheses; the full set of antibiotic concentrations used is in Dataset S1. (c) Diagrammatic summary of antibiotic targets in cell wall biosynthesis.

*S. coelicolor* A3(2) J1929 strains with mutations in *ppm1* (DT3017, DT1020, DT1029) and *pmt* (DT2008, DT1025) were isolated previously [21, 22] (see also Methods). The *ppm1* and *pmt* mutants display a small colony phenotype when growing on Difco nutrient agar or soya mannitol agar that could be complemented by plasmids pDT16 or pDT10, encoding the wild-type *ppm1* or *pmt*, respectively (Fig. S1). Microscopic techniques revealed no obvious morphological differences in the mutant strains so, to probe the underlying physiological changes, the *ppm1* and *pmt* mutants were subjected to antibiotic stress. Increased sensitivities to a number of antibiotics, measured using disc diffusion assays, were detected in multiple independently isolated *ppm1*^–^ and *pmt*^–^ mutant strains (Fig. 2; Dataset S1).

Whilst both *ppm1* and *pmt* mutants had increased susceptibility to ampicillin, imipenem, meropenem, rifampicin and vancomycin compared to the parent strain, the *ppm1* mutants also had greater sensitivity to daptomycin, tunicamycin, bacitracin, nisin and ramoplanin. Complementation of the *ppm1*^–^ and *pmt*^–^ mutations with their corresponding wild-type alleles alleviated the antibiotic-susceptible phenotypes (Fig. 2). The *ppm1* and *pmt* mutant strains showed no change in susceptibility to d-cycloserine, polymyxin, monensin, tetracycline, kanamycin, thiostrepton, chloramphenicol, ciprofloxacin and moenomycin, arguing against a general increase in permeability underlying the antibiotic susceptible phenotype. Overall, with the exception of rifampicin, which targets RNA polymerase, the antibiotics with increased efficacy on the *ppm1^–^* and *pmt^–^* mutants act at different stages of cell wall biosynthesis (Fig. 2).

### The vancomycin resistance cluster is induced by vancomycin in the *ppm1* and *pmt* mutants

**Fig. 3.**
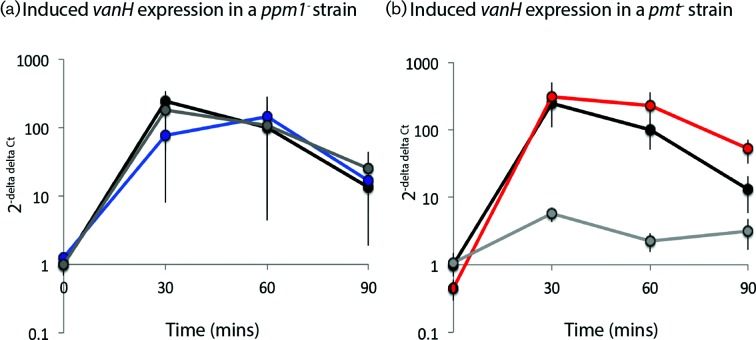
Transcription of *vanH* in response to vancomycin induction. qRT-PCR was performed to measure relative levels of *vanH* mRNA following addition of 0.1 µg ml^−1^ vancomycin to (a) DT3017 (blue; *ppm1*^–^) and (b) DT2008 (red; *pmt*^–^) strains alongside the parent strain (black;J1929) and their respective complemented strains (grey; panel a; DT3017:pDT16 and panel b; DT2008:pDT10). In each instance *hrdB* was used as the reference gene and J1929 time-point 0 as the reference sample.

*S. coelicolor* encodes a well-characterized vancomycin resistance mechanism conferring a MIC of approximately 80 µg ml^−1^ [33, 34]. This mechanism of resistance is shared with the pathogenic bacterium *Enterococcus faecium* and involves reprogramming of cell wall biosynthesis so that the vancomycin target of the peptidoglycan pentapeptide, the terminal d-Ala-d-Ala, is replaced by d-Ala-d-Lac [35–37]. The *S. coelicolor* resistance cluster consists of seven genes split into four operons: *vanRS*, a two-component signal transduction system, *vanHAX*, encoding enzymes required for the production of d-Lac, ligation of d-Ala to d-Lac and breakdown of d-Ala-d-Ala, respectively; *vanK*, encoding a Fem protein, a non-ribosomal peptidyltransferase that adds the cross-bridge amino acids to the stem pentapeptide ending in d-Ala-d-Lac; and *vanJ*, required for resistance to the structurally related antibiotic teicoplanin [33, 34, 38]. The addition of vancomycin, but not teicoplanin, induces expression of the *van* cluster genes. To test whether the *van* cluster was being induced in the *ppm1* and *pmt* mutants, we measured the level of *vanH* mRNA (Fig. 3; Dataset S2). As the *ppm1* and *pmt* mutant strains are highly sensitive to vancomycin, we used a very low concentration of the drug to induce (0.1 µg ml^−1^ vancomycin). Even at this low concentration the *van* cluster in the parent strain and in DT2008 is induced between 10- and 100 -old. The *ppm1* mutant strain also showed induction of *vanH,* but the extent varied widely from experiment to experiment leading to a large standard error. This observation and the low levels of induction in the complemented *pmt*^–^ strain are most likely due to the use of a vancomycin concentration for induction that is at the lower limit. We conclude that failure to transcribe the *van* cluster in the *ppm1* or the *pmt* mutants is not the reason why these strains are highly sensitive to vancomycin, and that delivery of resistance in these strains is blocked post-transcriptionally.

### Loss of Ppm1 activity leads to a global switch from catabolism to anabolism, including a change in fatty acid metabolism

In order to further our understanding of the underlying processes that led to a loss of intrinsic antibiotic resistance, global transcriptional profiling using RNAseq was performed in the *ppm1* mutant, DT3017, the original isogenic parent, J1929, and the complemented strain, DT3017:pDT16. A *ppm1*^–^ mutant was chosen for RNAseq analysis because of the more extreme phenotype of the *ppm1*^–^ mutants. Samples were collected during growth under controlled fermentation conditions where DT3017 showed only a modest growth defect (Fig. S2). Deseq analysis revealed that expression of 658 genes had changed with a *P*<0.05 level of significance between DT3017 and J1929 (461 with a >2-fold difference); 574 of these changes showed at least some restoration towards J1929 levels in DT3017:pDT16 and were used for further gene ontology analysis (Dataset S3). The majority (69 %) of the 574 genes used for gene ontology analysis were down-regulated in the *ppm1* mutant compared to the parent, J1929.

Gene ontology analysis showed that membrane and periplasmic functions had been severely affected in the *ppm1* mutant. The most prevalent down-regulated genes are associated with the GO terms transport and catabolic processes and in particular organic acid, carbohydrate and amino acid transport, and carboxylic acid, fatty acid and lipid catabolism are significantly down-regulated (Dataset S4; Table 1). The GO terms that describe the up-regulated genes are broadly biosynthetic processes; these notably involve carboxylic acids (31 genes), which include fatty acid (9 genes) and amino acid biosynthesis (19 genes). Sixty-six genes reached the Benjamini–Hochberg level of significance and were at least partially restored through complementation (Table S2). Almost half of the genes in the Benjamini–Hochberg set for which a function is predicted have a putative role in lipid metabolism.

**Table 1.** Enriched GO terms

	Count of genes associated with the listed GOID in the dataset	Count of genes associated with the listed GOID in the full gene list	*P* value
Up-regulated enriched GO terms			
Organic acid biosynthetic process	31	276	0.000
Cytoplasm (cellular component)	42	984	0.004
Fatty acid synthase activity (molecular-function)	5	25	0.034
Nickel cation binding (molecular-function)	4	13	0.029
Carboxylic acid biosynthetic process	31	268	0.000
Nucleoside catabolic process	3	7	0.053
Nucleoside phosphate biosynthetic process	9	109	0.078
Cellular amino acid biosynthetic process	19	179	0.000
Monocarboxylic acid biosynthetic process	12	82	0.000
Nucleoside monophosphate biosynthetic process	6	43	0.053
Glutamine family amino acid biosynthetic process	10	35	0.000
Alpha-amino acid biosynthetic process	18	150	0.000
Fatty acid biosynthetic process	9	64	0.003
Branched-chain amino acid biosynthetic process	5	19	0.010
Ribonucleoside monophosphate biosynthetic process	6	39	0.034
Biotin biosynthetic process	3	7	0.053
Arginine biosynthetic process	7	15	0.000
Leucine biosynthetic process	3	7	0.053
‘*De novo*' UMP biosynthetic process	3	7	0.053
Down-regulated enriched GO terms			
Periplasmic space (cellular-component)	17	83	0.002
Transport	57	693	0.054
Single-organism catabolic process	19	151	0.069
Organic substance transport	31	261	0.008
Organic acid transport	13	69	0.020
Carbohydrate transport	13	80	0.062
Anion transport	16	97	0.018
Monocarboxylic acid catabolic process	14	63	0.003
Carboxylic acid transport	13	69	0.020
Fatty acid metabolic process	16	108	0.053
Fatty acid catabolic process	10	27	0.002
Amino acid transport	13	61	0.008

**Fig. 4.**
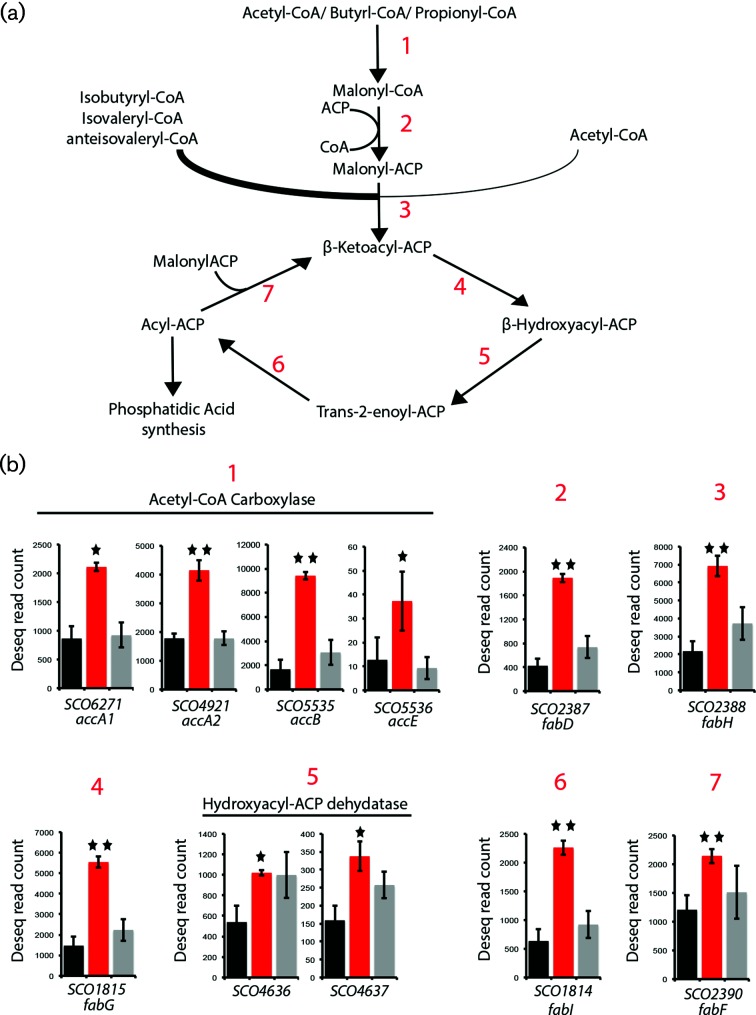
Expression of genes involved in fatty acid metabolism in the *ppm1*^–^ strain DT3017, the parent strain, J1929 and the complemented strain DT3017:pDT16. (a) Pathway for the synthesis of fatty acids in *S. coelicolor*. The thick line feeding into reaction 3 reflects the preference by *S. coelicolor* FabH for branched-chain acyl-CoA precursors [83] (b) Deseq normalized expression levels for fatty acid metabolism genes of *S. coelicolor* for strains J1929 (black), DT3017 (red) and DT3017 pDT16 (grey). Red numbering above refers to steps in the pathway indicated in (a) above. Shown values are the average of three replicates. Above DT3017 one star equals *P*<0.05 and two stars equal Benjamini–Hockberg-corrected *P*<0.05 significance levels against J1929.

Closer inspection of fatty acid metabolism revealed an up-regulation of all the components of the *S. coelicolor* ACCase and FAS II complex (Fig. 4) and a general down-regulation of a variety of genes predicted to perform β-oxidation and fatty acid degradation. These results suggest up-regulation of fatty acid production in DT3017, and hence possible alterations to membrane composition. Alterations to membrane fluidity, topology and composition are all known to effect stability and sensitivity to stress agents such as antibiotics [39–42].

The enriched gene set was searched for genes that might be involved in cell wall biosynthesis, and/or antibiotic targets. Genes that were up-regulated and might be directly involved in peptidoglycan biosynthesis encode an FtsI-like PBP (*sco3771*), a putative muramoyl-pentapeptide carboxypeptidase (*sco5467*) and a putative deacetylase (*sco2962*). Genes that were down-regulated encode a putative PG deacetylase (*sco6178*), a putative AmpC β−lactamase/ d-alanyl-d-alanine carboxypeptidase (*sco5660*), a putative d-alanyl-d-alanine dipeptidase (*sco1396*) and a putative murein dd-endopeptidase (*sco0543*). Enriched GO terms did not include cell envelope biogenesis, and the expression of the putative major PG biogenesis operon in *S. coelicolor* (*sco2077-sco2092*) was unchanged in DT3017. Amongst the down-regulated gene set, there were no obvious candidates that might contribute to enhanced antibiotic susceptibility. On the contrary, genes that are predicted to encode efflux systems were up-regulated (*sco5950*, *sco5516*, *sco3206*, *sco1742* and *sco0756*) as well as one gene, *sco4049*, encoding a putative penicillin amidase/acylase. Two up-regulated genes have been linked with vancomycin resistance: *sco6183* (*cwgE*), a GT1 putative heptosyl transferase that is part of the vancomycin-induced putative cell wall glycan operon (*cwg*) [43], and *sco2472,* a homologue of an uncharacterized protein, SanA, that affects membrane permeability to vancomycin [44].

**Fig. 5.**
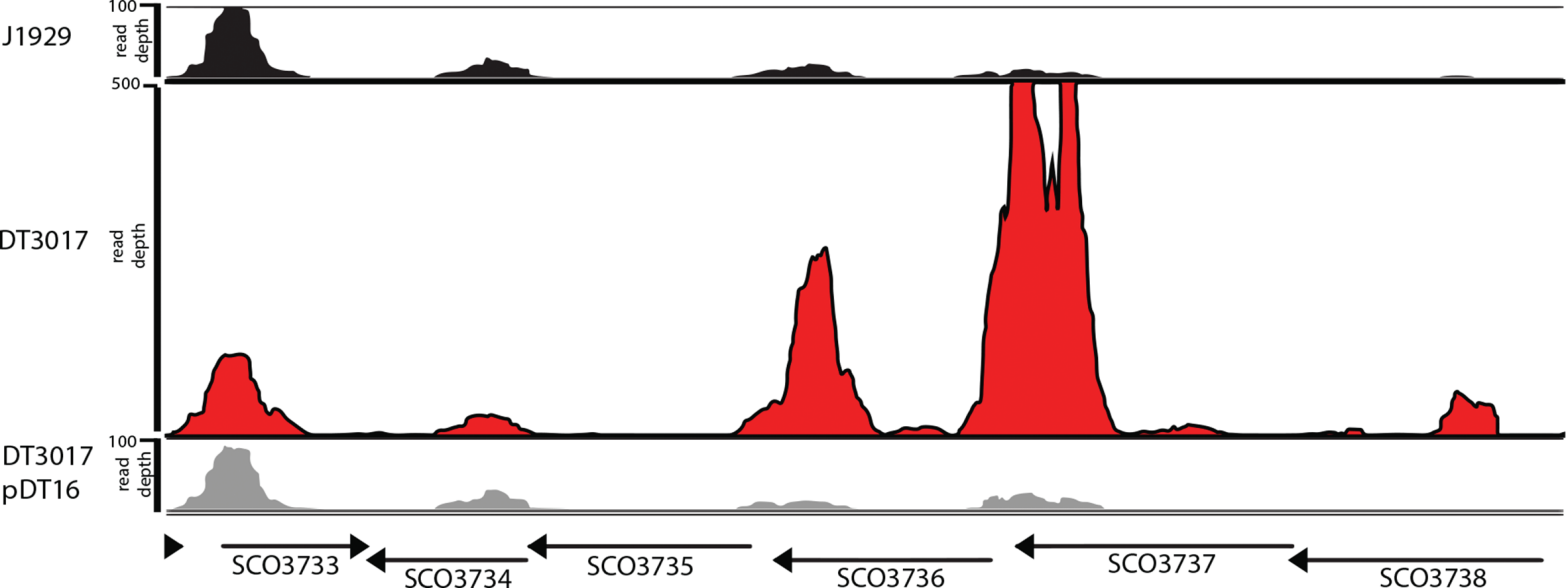
RNAseq mapped read depth across SCO3736–SCO3738, the genes most significantly changed in DT3017. Deseq analysis of differential gene expression between J1929 and DT3017 revealed that the top three most significant changes occurred in one operon, SCO3736–SCO3738, encoding a putative extracytoplasmic sigma factor, lipoprotein and integral membrane protein, respectively. Shown are the mapped read depths across this region alongside downstream SCO3735, a putative secreted protein, as visualized by the Integrated Genomics Viewer. Replicate one is shown as representative of all replicates.

The gene *sco3736*, encoding an ECF sigma factor and the two upstream genes *sco3737* and *sco3738*, encoding a putative lipoprotein/sortase (homology to SrtE family – cd05829; E=3e^−51^) and membrane protein, respectively, are the most significantly up-regulated genes in the *ppm1*^–^ strain, DT3017 (13-, 37- and 32-fold, respectively) (Fig. 5). The ECF sigma factor *sco3450* is also 1.9 fold up-regulated and there is a strong down-regulation (15-fold) of ECF sigma factor gene, *sco4938*. It is worth emphasizing that the expression of *sco3356* encoding the known cell wall stress ECF sigma factor, SigE, [45] did not change (Dataset S3).

### The Ppm1-defective strain contains a higher relative proportion of unsaturated fatty acids

Raman spectroscopy was used to investigate changes in the fatty acid profile of the *ppm1* mutant by means of quantitative lipid phenotyping. The Raman spectroscopy approach involves the inelastic scattering of light to produce high-discriminatory, molecular-scale cell characterization (i.e. fingerprinting). Raman fingerprinting provides detailed information on the cellular components including proteins, nucleic acids, lipids and carbohydrates [31, 46], and has been used to characterize microbial species and subspecies, and phenotypic changes [32, 47–49].

Raman spectroscopy was previously applied to lipid profiling in several studies. Wu *et al.* correlated the relative intensities of the 1650 cm^−1^ C=C and 1440 cm^−1^ CH_2_ Raman bands (*I_1650_*/*I_1440_* ratio) to the degree of lipid unsaturation in extracted lipids from algae cells [50]. Potcoava *et al.* used the degree of unsaturation (*I_1655_*/*I_1440_* and *I_1655_*/*I_1294_*), lipid chain length (*I_2930_*/*I_2959_*) and total unsaturated fatty acid (TUFA) to total fatty acid (TFA) (*I_3015_*/*I_2851_*) ratios to characterize lipid droplets in treated and untreated breast and prostate cancer cells, noting that the TUFA/TFA ratio correlates to the number of C=C to CH_2_ species in pure fatty acids [51]. The TUFA/TFA ratio has also been used by Nieva *et al.* to stratify the degree of malignancy in breast cancer cells, and is regarded as a key Raman biomarker for lipid phenotyping [52]. Whereas peak intensity ratios for the degree of lipid unsaturation and chain length also relate to proteins, the TUFA/TFA ratio comprises lipid molecule vibrations only [31] and is therefore a reliable biomarker to interrogate the fatty acids in intact cells. In this work, the TUFA/TFA ratio was used to determine changes in the lipid content of the parent *S. coelicolor strain* J1929, the *ppm1* mutant derivative, DT3017 and the complemented mutant, DT3017:pDT16.

**Fig. 6.**
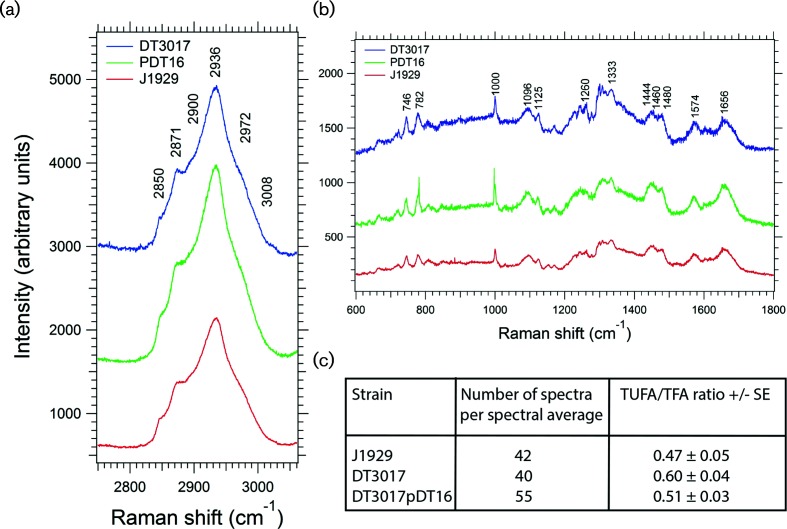
Raman spectroscopy analysis of *S. coelicolor* strains. Averaged Raman spectra obtained in the (a) high-wave number region and (b) the fingerprint region (600–1800 cm^−1^) for the *S. coelicolor* parent strain J1929 (red; *N*=42), the *ppm1*^-^ mutant strain DT3017 (*blue*; *N*=55) and the complemented mutant, DT3017:pDT16 (red; *N*=40). *N* refers to the total number of individual spectra in each spectral average. Key peak assignments are shown (Table S3) with the spectra vertically shifted to aid visualization. Panel c; TUFA/TFA (*I_3008_*/*I_2850_*) peak intensity ratios derived from the averaged spectra in panels a and b.

Averaged Raman spectra and peak assignments obtained for J1929, DT3017 DT3017:pDT16 (Figs 6 and S3, Table S3) agree with previously published spectra for *Streptomyces* cells [32] and other published works on bacteria [48]. The TUFA/TFA peak intensity ratios determined from these spectra showed that the parent strain, J1929, and the complemented mutant DT3017:pDT16 have similar lipid composition (Fig. 6c). For the DT3017 *ppm1* strain, the TUFA/TFA value was outside of the J1929 and DT3017:pDT16 ranges, confirming that it has a larger relative unsaturated fatty acid component and hence different lipid composition. The converged percentage standard error of the mean for the TUFA/TFA values as a function of the number of randomly selected individual spectra in the spectral averages indicated that sufficient data were collected (*N*=40–55 spectra/strain) to establish reliable statistics for these measurements (Fig. S3).

## Discussion

Previous work has shown that mutations in the genes encoding Ppm1 and Pmt required for synthesis of polyprenol phosphate mannose (PPM; *ppm1*) and O-glycoproteins (*pmt* and *ppm1*) in *Streptomyces coelicolor* lead to phage resistance [9, 21, 22]. Here we show that *pmt*^–^ and *ppm1*^–^ mutations also lead to an increase in susceptibility to antibiotics, particularly those that target cell wall biogenesis. We propose that in *ppm1*^–^ strains, the extremely antibiotic-susceptible phenotype is due to global changes in lipid, membrane and periplasmic protein functions. The *pmt* mutants have a similar but less extreme phenotype to the *ppm1* mutants, suggesting that loss of membrane and periplasmic glycoproteins contributes to antibiotic hyper-susceptibility.

The *ppm1* mutants show greater sensitivity to vancomycin and β-lactams, and higher susceptibility to a greater range of antibiotics that target membrane or periplasmic enzymes than *pmt*^–^ mutant strains (Fig. 2). *ppm1* mutants fail to generate the lipid-linked mannose donor, polyprenol phosphate mannose, PPM, whereas *pmt*^–^ strains are able to generate PPM but fail to use PPM in protein O-glycosylation [9]. *Streptomyces* spp. have been shown to produce phosphotidylinositolmannosides (PIMs) whose synthesis could also require PPM, depending on the degree of mannosylation [53, 54]. In mycobacteria myo-inositol is sequentially mannosylated generating PIM_1_ to PIM_6_, with PPM acting as the sugar donor for the later steps in the pathway [55]. *S. coelicolor* contains close homologues of the *M. tuberculosis* genes required for PIM biosynthesis:PimA (Rv2610c, SCO1525; E=3e^−122^) and PimB (Rv2188c, SCO2132; E=9e^−127^) that synthesize *myo*-inositol with one (PIM_1_) or two (PIM_2_) mannose residues attached, respectively. Both PimA and PimB act on the inner face of the cytoplasmic membrane and use GDP-mannose as the sugar donor. PimE in *M. tuberculosis* uses PPM to transfer mannose to the growing mannose chain, i.e. PIM_4_ to PIM_5_. SCO2335 is a distant (E=4e^−15^) homologue of *M. tuberculosis* PimE (Rv1159) and could be involved in the polymerization of the mannoside chain using PPM as the sugar donor. Another candidate protein from *S. coelicolor* that could utilize PPM is SCO4023, which is annotated as a dolichyl-phosphate-mannose protein mannosyltransferase (PMT_2, Pfam13231). The gene *sco4023* is located adjacent to *sco4022,* which is a homologue of *ppm1*. In preliminary work we have knocked out both of these genes, and mutation in neither gene confers resistance to ϕC31 (Dr Anpu Varghese, unpublished work). According to the Carbohydrate Active Enzymes (CAZY) database (www.cazy.org), the *S. coelicolor* genome encodes 29 and 17 GT2 and GT4 family glycosyl transferases, respectively; members of both families can use polyprenol-containing glycoconjugates as substrates. Loss of PPM from the *S. coelicolor* membrane is therefore expected to affect synthesis of periplasmic O-glycoproteins, periplasmic mannosylation of PIMs and, possibly, a variety of as yet uncharacterized cell envelope macromolecules. While disruption of the O-glycosylation pathway by mutation of *pmt* leads to mild antibiotic hypersusceptibility, the extreme antibiotic hyper-susceptibility when PPM is depleted indicates that multiple pathways depend on PPM as a sugar donor.

RNAseq was used to provide insight into the global changes in the *ppm1*^–^ mutant strain compared to its parent. The *ppm1*^–^ strain has undergone a major shift from a largely catabolic metabolism to an anabolic one. Genes coding for membrane and periplasmic proteins, particularly transport proteins, were down-regulated in the *ppm1* mutant, DT3017, whereas genes for biosynthetic cytoplasmic functions were up-regulated. This response might be expected after transfer of a bacterial culture to a minimal medium where biosynthetic functions would be up-regulated. However, the growth of all the strains for RNAseq was performed in rich medium (Difco nutrient broth). The logical interpretation of the switch from the parental catabolic metabolism to the *ppm1* mutant anabolic metabolism is that some systems for sensing nutrients and their uptake are defective. At this stage we cannot, however, propose a direct mechanism through which loss of PPM might lead to the observed metabolic switch, other than multiple effects caused by a lack of mannose-containing glycoconjugates in the cell envelope and/or an accumulation of polyprenol phosphate.

Differential gene expression showed a switch in fatty acid metabolism in the *ppm1*^–^ strain, with genes coding for β-oxidation enzymes down-regulated and those for synthesis up-regulated (Table 1; Fig. 4) [25, 56]. The up-regulation of the ACC complex might have increased the proportion of straight-chain fatty acids as ACC, but not the PCC complex, can use acetyl-CoA as a primer for FA biosynthesis [56]. To study lipids in the *Streptomyces* strains, we used Raman spectroscopy of whole cells. The *ppm1*^–^ mutant was shown to have a small but significant increase in the ratio of TUFA acids to total fatty acids (TFA) compared to the TUFA/TFA ratios in the parent strain or the complemented mutant. The only enzyme in *Streptomyces* known to introduce the C=C double bond in FA biosynthesis is FabA (SCO4636 and SCO4637), a hydroxyacyl-ACP dehydratase [57], and the corresponding genes are up-regulated in the *ppm1* mutant. Up-regulation of *fabA* in *E. coli* is known to lead to a higher proportion of unsaturated FAs [25, 58, 59]. However, it is not clear whether up-regulation of the *fabA* genes in the *S. coelicolor ppm1^-^* mutant led directly to the observed higher TUFA/TFA ratio or whether the up-regulation is simply in line with the overall up-regulation of the FA biosynthesis pathway. The net result, however, is a change in lipid composition in the *ppm1*^–^ strain DT3017 compared to its parent.

Changes in lipid composition can have profound effects on the activities of membrane proteins, perhaps providing a mechanism for the proposed defects in periplasmic nutrient sensing and transport protein function. Membrane composition is important in maintaining the topology of membrane proteins. Although the ‘positive inside rule’ is believed to be the major determinant of membrane protein topology, head group charge, fatty acid chain length and fatty acid unsaturation have also been shown to be important [60]. Pertinent to our work is that changes in membrane composition are also correlated with changes in sensitivity to antibiotics and/or detergents. For example, a *B. subtilis* strain lacking three extracytoplasmic functions, ECF, sigma factors (σM, σW and σX) is significantly more sensitive to cell wall-acting antibiotics than either the double mutants or the parents [61]. Expression of σW was shown to lead to a decrease in membrane fluidity with concomitant increase in resistance to detergents [42]. The *S. coelicolor ppm1* mutants are highly susceptible to daptomycin, an antibiotic that targets the cytoplasmic membrane, possibly causing depolarization, and in *Enterococcus faecalis* inhibits cell division by concentrating at the septum [62]. Changes in membrane fluidity have been associated with daptomycin resistance in *S. aureus*; *in vitro-*derived daptomycin-resistant strains have increased membrane fluidity, although highly fluid membranes also display increased resistance to cationic peptides including daptomycin, supporting the view that membrane fluidity is only one component of the cell envelope that mediates susceptibility to this antibiotic [39, 62, 63]. A decrease in the supply of undecaprenol phosphate (*uppS* mutant) in *B. subtilis* can lead to increased vancomycin resistance, and this was shown to be due to elevated σM expression. Mutation in a teichoic acid biosynthesis gene (TagF) can also increase sensitivity to vancomycin and lysozyme in *Streptomyces* [64].

Gene regulation by ECF sigma factors is important in *B. subtilis* in sensing and reacting to cell wall stresses [65]. The RNAseq data on the *S. coelicolor ppm1*^-^ mutant showed significant changes in expression of regulators, but not where expected. For example, genes for sigma factors such as SigE (*sco3356*, linked to cell wall stress; [45]), SCO0600 (*sigB*; osmotic stress; [66]), SCO2954 (linked to increased expression of extracellular proteases; [67]), SCO4005 (up-regulated by ppGpp; [68]) and SCO4938 were down-regulated in the *ppm1*^–^ strain DT3017. Three of four of the most significantly up-regulated genes in DT3017 were the consecutive genes *sco3736–sco3738*, probably co-transcribed and encoding a putative ECF sigma factor, a lipoprotein with putative sortase E function, and integral membrane protein, respectively (Fig. 5). Sortases are transpeptidases that attach proteins to the cell wall [69]. The major ‘housekeeping’ sortase normally recognizes a conserved pentapeptide sorting sequence in a variety of substrates that in *S. coelicolor* is predicted to be LAXTG [70]. Sortase E homologues are thought to have a more specific range of substrates, and two of these, SCO3849 and SCO3850, have been shown to be required for normal development in *S. coelicolor* [71]. SCO3737 belongs to a more divergent group of putative sortases and it has been proposed that genes for this group are co-transcribed with their substrates. In the case of *sco3737,* this would be *sco3738* encoding a putative membrane protein [69, 71]. A gene encoding a second putative ECF sigma factor, *sco3450* (SigR2 transcribed by SigR, which mediates the major thiol-oxidative stress response) [72, 73], is also up-regulated. Both up-regulated sigma factors are expressed early in the life cycle of *Streptomyces*; *sco3736* and *sco3450* were identified in a study examining regulatory networks during germination [73].

The changes in the complement of sigma factors in DT3017 might be directly responsible for the global phenotypic response to growth without the ability to synthesize PPM. Changes in sigma factors might also explain why *ppm1* and *pmt* mutants are more sensitive to rifampicin, an antibiotic inhibiting transcription initiation by binding within the DNA/RNA channel of the β-subunit of RNA polymerase [74]. Although rifampicin is considered a general inhibitor of RNA polymerase, there are examples of altered promoter responses to rifampicin for differing holo-enzymes. These include: the *tdc* operon is insensitive to rifampicin [75] and there is increased sensitivity to rifampicin *in vivo* and *in vitro* for σ^70^-RNApol over σ^32^-RNApol in *E. coli* [76]. In *B. subtilis* SigB dependent transcription is less sensitive to rifampicin [77], and transcription of a *sigR-*dependent promoter is more resistant to rifampicin than transcription of a *hrdB-*dependent promoter (*rrn*) [78].

The *pmt* mutants are, like the *ppm1* mutants, considerably more sensitive to vancomycin and β**-**lactams (including ampicillin and imipenem) than the parent strain (Fig. 2). Our hypothesis, which is consistent with the mechanism of protein mannosyl transferases in yeasts and in another actinobacterium, *M. smegmatis*, is that secreted and membrane proteins are targets for O-glycosylation [2, 9, 13] and that the modification affects protein function. It has been established by others that protein glycosylation can have an impact on protein function [79, 80], and it has recently been shown to have a role in regulating the function of a peptidoglycan hydrolase [81]. The relative activities of transpeptidase and carboxypeptidase cell wall biosynthesis enzymes are known to play a role in providing intrinsic vancomycin resistance in *S. coelicolor*, suggesting that subtle changes to protein activities could have a large effect on antibiotic sensitivities [82]. Our observations are reminiscent of the phenotypes of PMT mutants of fungi, where the most common phenotype is increased susceptibility to cell wall-acting inhibitors [2]. The consistency across kingdoms in the phenotypes of *pmt* mutants implies that O-glycosylation has similar roles in protein function in fungi and bacteria.

Both *pmt* and *ppm1* mutants are highly susceptible to vancomycin, despite the presence in *S. coelicolor* of the *vanRSJHAX* gene cluster conferring resistance [33, 34]. This cluster is induced by vancomycin, which binds the VanS sensor protein and, through phosphorylation of VanR, switches on the *van* promoters. We showed that the *van* genes are induced normally by vancomycin in the *ppm1* and *pmt* mutants, and yet the strains are still very sensitive to the antibiotic. There are two possible explanations for the persistent sensitivity to vancomycin: either the substrates for the resistance enzymes to generate the d-ala-d-lac pentapeptides are in limited supply or resistance could be masked epistatically by membrane-associated steps such as flipping of lipid II, or the lipid II polymerization steps transglycosylation and transpeptidation. In the *ppm1* mutant, changes in membrane physiology are predicted by RNAseq analysis and observed through Raman spectroscopy, arguing that perturbation of membrane-associated steps in peptidoglycan biosynthesis might be responsible for preventing the normal ability of *van* gene products to deliver resistance to vancomycin.

In summary we have provided insights into the complex phenotypes of mutants in *S. coelicolor* that lack the ability to synthesize PPM and *O*-glycoproteins. We have presented evidence for a remodelling of the plasma membrane in terms of the protein and fatty acid components, possibly mediated by changes in ECF sigma factors in the *ppm1*^–^ mutant. The result of these adaptations is a loss of intrinsic resistance to antibiotics that target cell wall biogenesis, particularly those that act outside the plasma membrane, and to rifampicin. As the *pmt*^–^ mutant strains have also lost some intrinsic resistance to a similar subset of antibiotics (particularly vancomycin, imipenem and rifampicin) as the *ppm1*^–^ strain, we conclude that loss of protein O-glycosylation contributes in part to the phenotype of the *ppm1*^–^ strains.

## Supplementary Data

2912 Supplementary File 1
