# Erratum: *Streptomyces coelicolor* strains lacking polyprenol phosphate mannose synthase and protein O-mannosyl transferase are hyper-susceptible to multiple antibiotics

**DOI:** 10.1099/mic.0.000647

**Published:** 2018-04-05

**Authors:** Robert Howlett, Nicholas Read, Anpu Varghese, Charles Kershaw, Y. Hancock, Margaret C. M. Smith

**Affiliations:** ^1^​ Department of Biology, University of York, York, UK; ^2^​ Institute of Medical Sciences, University of Aberdeen, Aberdeen, UK; ^3^​ Department of Physics, University of York, York, UK; ^4^​ York Centre for Complex Systems Analysis, University of York, York, UK

An error occurred in the process of publishing this article. In the second sentence of the abstract, ‘synthesiszed’ is incorrect and should read ‘synthesized’.

The Microbiology Society apologizes for any inconvenience caused.

