# Supplementary material for: Streptomyces coelicolor strains lacking polyprenol phosphate mannose synthase and protein O-mannosyl transferase are hyper-susceptible to multiple antibiotics
Source: Microbiology (Reading). 2018 Feb 1;164(3):369–82. doi: 10.1099/mic.0.000605 (PMC5882110; doi:10.1099/mic.0.000605)
Supplement: Supplementary File 1 [file mic-164-369-s001.pdf]

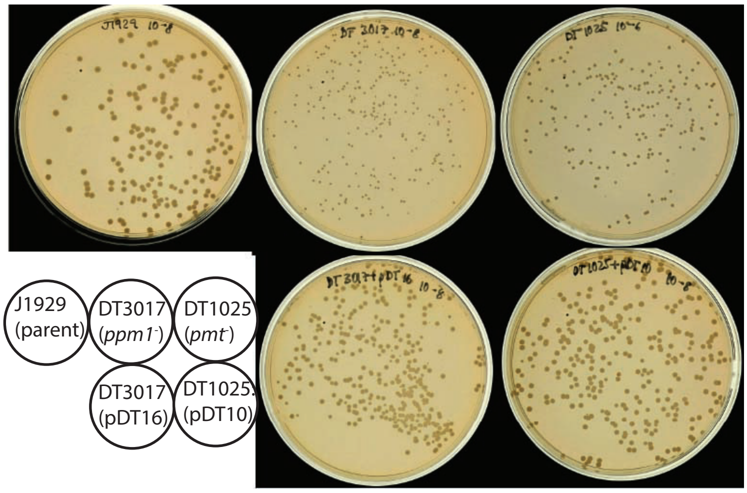
J1929
(parent)
DT3017
($ppm1^-$)
DT1025
(pmt^-)
DT3017
(pDT16)
DT1025
(pDT10)

Figure S1

A

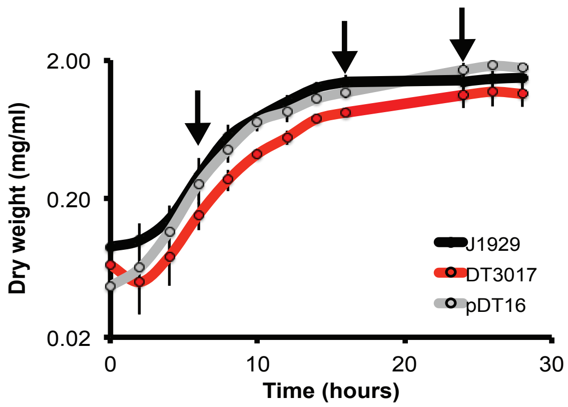
2.00
0.20
0.02
Dry weight (mg/ml)
0
10
20
30
Time (hours)
J1929
DT3017
pDT16

B

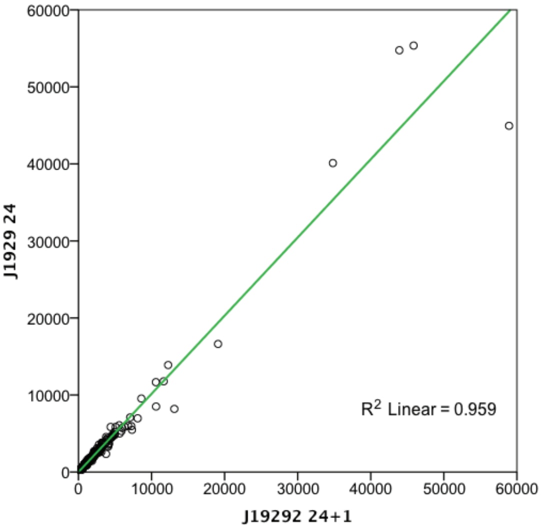
60000
50000
40000
30000
20000
10000
0
J1929 24
R2 Linear = 0.959
0
10000
20000
30000
40000
50000
60000
J19292 24+1

Figure S2

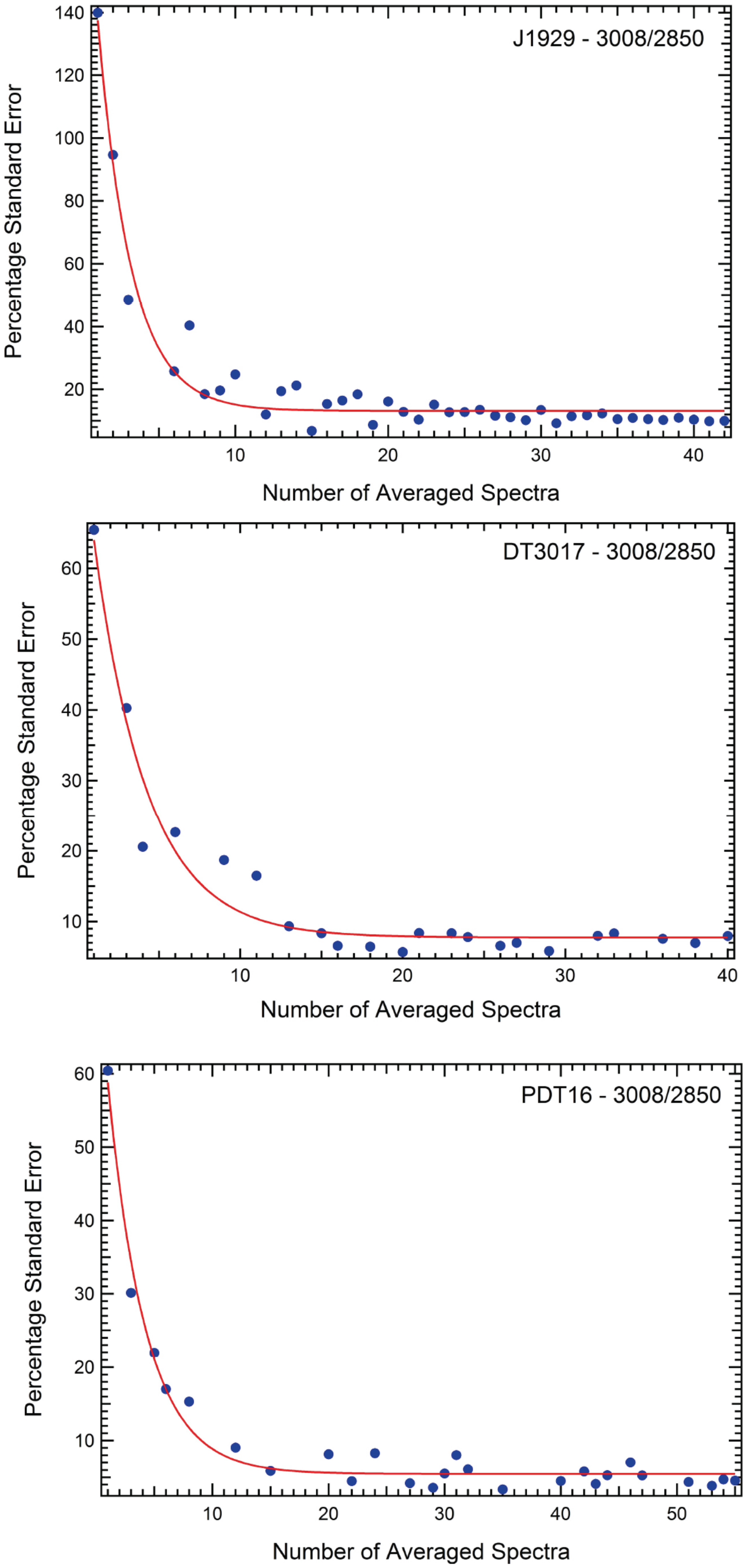

J1929 - 3008/2850
Percentage Standard Error
Number of Averaged Spectra
140
120
100
80
60
40
20
10
20
30
40
DT3017 - 3008/2850
Percentage Standard Error
Number of Averaged Spectra
60
50
40
30
20
10
10
20
30
40
PDT16 - 3008/2850
Percentage Standard Error
Number of Averaged Spectra
60
50
40
30
20
10
10
20
30
40
50

Figure S3

Supplementary Figure legends (Howlett, Read *et al*)

Figure S1. Small colony phenotype in the *pmt*⁻ and *ppm1*⁻ mutants. S. coelicolor *pmt*⁻ (DT1025) and *ppm1*⁻ (DT3017) mutants are derived from J1929 (*pglY*⁻) [1-3]. Plasmids pDT10 and pDT16 used to complement *pmt*⁻ and *ppm1*⁻ mutants, respectively, are derivatives from vector pSET152 and encode wild type *pmt* and *ppm1*, respectively [2, 3]. Strains were grown on Difco nutrient agar and were incubated at 30 ^oC for 48 hours. Key to strains is bottom left.

Figure S2. Sampling of the transcriptome from *S. coelicolor* J1929, DT3017 and DT3017 pDT16 strains for RNAseq analysis. (*A*) *S. coelicolor* strains were cultured in controlled fermentor growth conditions in Difco nutrient broth where DT3017 (red) showed a modest growth defect compared to its parent and complement strains J1929 (black) and DT3017 pDT16 (grey) respectively. Samples were collected from all samples at time-point 6 for comparative transcriptome analysis and J1929 replicate 1 at time-point 24 to confirm transcriptome quenching (indicated by black arrows). Shown are averages of three biological repeats with error bars. (*B*) Validation of the sampling protocol for RNASeq. Two samples were collected from J1929 replicate 1 at time-point 24, one of which was left on ice for one hr following the addition of a stop solution; RNA from both samples was prepared and analyzed by RNAseq to look for significant changes that might have resulted from RNA degradation. Subsequent linear regression analysis was performed, giving an R^2 value of 0.959 indicating that the use of the stop solution was a valid procedure for sample preparation.

Figure S3. Convergence of the percentage standard error of the mean(SE) for the TUFA/TFA peak intensity ratio I_{3008}/I_{2850} as a function of the number of randomly chosen spectra comprising the spectral average for the parent strain (J1929, top panel), the *ppm1*⁻ mutant (DT3017, middle panel) and the complemented mutant (DT3017:pDT16, bottom panel). In total 42, 55 and 40 spectra were averaged for J1929, DT3017 and DT3017:pDT16, respectively.

References

1. **Bedford, D.J., C. Laity, and M.J. Buttner,** 1995. Two genes involved in the phase-variable fC31 resistance mechanism of *Streptomyces coelicolor* A3(2). J Bacteriol, **177**(16): p. 4681-9.
2. **Cowlishaw, D.A. and M.C.M Smith,** 2001. Glycosylation of a Streptomyces coelicolor A3(2) cell envelope protein is required for infection by bacteriophage fC31. Mol Microbiol, **41**(3): p. 601-10.
3. **Cowlishaw, D.A. and M.C.M Smith,** 2002. A gene encoding a homologue of dolichol phosphate-b-D mannose synthase is required for infection of *Streptomyces coelicolor* A3(2) by phage fC31. J Bacteriol, **184**(21): p. 6081-6083.

Table S1 Oligonucleotides used for qRT-PCR

Oligonucleotide	Sequence	Use
RH107	5' CTGATGGACGTCCTGAAGGT	*sigE* forward
RH108	5' GTCTCCTCCGTCGACATCTG	*sigE* reverse
RH109	5' ACGAGAATCACGGTTTACGG	*vanH* forward
RH110	5' CTTGTGGTCAATGCTGATGC	*vanH* reverse
RH111	5' CCGAGTCCGTCTCTGTCATG	*hrdB* forward
RH112	5' CGGAATCTGGTCAGCTTCGA	*hrdB* reverse

Table S2 Benjamin-Hockberg list

	log2 fold change DT3017 Vs		
Gene	J1929	DT3017 pDT16	Function
SCO3737	5.23	4.92	lipoprotein
SCO3736	3.76	3.35	RNA polymerase ECF sigma factor
SCO3738	5.02	4.81	hypothetical protein
SCO2774	-2.07	-0.91	acyl-CoA dehydrogenase
SCO3051	-1.88	-0.92	acyl-CoA dehydrogenase
SCO1182	1.66	0.13	hypothetical protein
SCO3706	2.24	1.71	ABC transporter ATP-binding protein
SCO1346	-1.67	-0.61	3-oxoacyl-ACP reductase
SCO2387	2.16	1.36	ACP S-malonyltransferase
SCO2776	-3.84	-2.13	acetyl/propionyl CoA carboxylase subunit beta
SCO1454	-2.61	-0.90	amino oxidase
SCO3707	2.03	2.35	lipoprotein
SCO3079	-1.81	-0.65	acetyl-CoA acetyltransferase
SCO0107	-2.17	-1.45	aminoglycoside nucleotidyltransferase
SCO1345	-1.89	-0.46	3-ketoacyl-ACP reductase
SCO5515	1.76	1.10	D-3-phosphoglycerate dehydrogenase
SCO4980	-2.01	-1.43	hypothetical protein
SCO6009	-1.61	-0.54	solute-binding protein
SCO2014	1.21	0.52	pyruvate kinase
SCO2779	-3.25	-1.54	acyl-CoA dehydrogenase
SCO5535	2.50	1.61	carboxyl transferase
SCO1459	-3.01	-1.01	amino acid transporter
SCO0215	-4.17	-3.84	hypothetical protein
SCO0174	-4.11	-4.53	DNA-binding protein
SCO2587	-1.35	-1.10	gamma-glutamyl kinase
SCO2773	-2.26	-0.27	acyl CoA thioesterase II
SCO1815	1.92	1.30	3-oxacyl-ACP reductase
SCO0165	-2.72	-2.50	hypothetical protein
SCO6010	-1.48	-0.37	ABC transporter ATP-binding protein
SCO2388	1.67	0.89	3-oxoacyl-ACP synthase
SCO0923	-3.69	-3.82	succinate dehydrogenase flavoprotein subunit
SCO1082	-1.35	-1.00	electron transfer flavoprotein subunit beta
SCO6011	-1.43	-0.27	ABC transporter
SCO4921	1.22	1.22	acyl-CoA carboxylase complex A subunit
SCO7036	1.69	0.75	argininosuccinate synthase
SCO0167	-3.29	-3.78	hypothetical protein
SCO1814	1.84	1.29	enoyl-ACP reductase
SCO4690	-3.41	-2.82	hypothetical protein
SCO0543	-1.85	-1.50	peptidase
SCO1081	-1.57	-1.04	electron transfer flavoprotein subunit alpha
SCO5676	-2.28	-0.44	4-aminobutyrate aminotransferase
SCO5516	1.51	0.92	integral membrane efflux protein
SCO1453	-1.83	-0.41	hypothetical protein

SCO0166	-2.86	-3.17	regulator
SCO6502	-2.74	-3.10	gas vesicle synthesis protein
SCO6564	1.57	1.34	3-oxoacyl-ACP synthase
SCO0168	-3.60	-4.13	regulator protein
SCO3607	-2.02	-1.37	hypothetical protein
SCO4107	1.22	0.89	hypothetical protein
SCO3656	1.68	0.35	hypothetical protein
SCO0211	-4.01	-4.22	hypothetical protein
SCO1225	-1.84	-0.99	osmoprotectant transporter
SCO3286	-2.62	-0.95	hypothetical protein
SCO4255	-1.64	-1.26	hypothetical protein
SCO2492	-4.03	-1.84	hypothetical protein
SCO2391	1.39	0.79	hypothetical protein
SCO2561	-1.03	-0.42	long-chain fatty-acid CoA ligase
SCO1455	-2.31	-0.79	hydrolase
SCO7469	-3.47	-1.25	phenylacetate-CoA ligase
SCO1222	-3.07	-1.14	hypothetical protein
SCO0426	-2.57	-1.74	hypothetical protein
SCO1342	1.55	0.01	hypothetical protein
SCO1457	-2.16	-0.36	transporter
SCO6731	-1.28	-0.07	acetyl-CoA acetyltransferase
SCO0596	-2.02	-2.97	DNA-binding protein
SCO3287	-2.59	-1.40	serine/arginine rich protein

Table S3: Peak positions averaged across the *Streptomyces coelicolor* J1929 (wild-type parent strain), the DT3017 (*ppm1*$^-$ mutant strain) and DT3017:pDT16 (the complemented mutant strain) with a maximum uncertainty of ±3cm^{-1} obtained from the average. The TFA band (2850 cm^{-1}) and TUFA band (3008cm^{-1}) are highlighted.

**Peak position (cm^{-1})**	**Assignment**
746	DNA/RNA*
782	DNA/RNA*
1000	Phenylalanine*
1096	DNA/RNA & nucleic acids*
1125	Lipids*
1260	Amide III*
1333	DNA/RNA*
1444	Lipids (CH_2 deformation)*, proteins*
1460	Lipids*, proteins*, DNA*
1480	Amide II*, DNA§
1574	DNA*§
1656	Lipids (C=C)*, proteins*
2850	Lipids (CH_2) **TFA band**
2871	Lipids (CH_2)*, proteins*
2900	Lipids (CH stretch), proteins*
2934	Lipids (CH_2 asymmetric stretch)*, proteins*
2970	Lipids (CH_3)*
3008	=CH stretch lipids* **TUFA band**

* Movasaghi *et al.*, 2007.
§ Walter *et al.*, 2012.

Dataset S1; Disc diffusion assays

		J1929						*DT1020 ppm1-*						Vs *J1929*	*DT1020 pDT16*						Vs *J1929*	Vs *DT1020*
**Antibiotic**	**conc (µg/disc)**	**1**	**2**	**3**	**4**	**AVERAGE**	**SEM**	**1**	**2**	**3**	**4**	**AVERAGE**	**SEM**	**P-Value**	**1**	**2**	**3**	**4**	**AVERAGE**	**SEM**	**P-value**	**Pvalue**
Ampicillin	40	0	0	0	0	0	0.00	20	16	20	19	19	0.95	**0.00**	0	0	0	0	0	0.00	#DIV/0!	**0.00**
Bacitracin	4	11	12	8	9	10	0.91	14	16	14	12	14	0.82	**0.02**	0	12	11	10	8	2.78	0.59	0.13
Bacitracin	40	19	20	18	18	19	0.48	24	27	24	23	25	0.87	**0.00**	20	21	18	18	19	0.75	0.60	**0.00**
Daptamycin	40	0	0	0	0	0	0.00	25	21	21	17	21	1.63	**0.00**	0	0	0	0	0	0.00	#DIV/0!	**0.00**
Imipenem	4	0	0	0	0	0	0.00	35	34	10	12	23	6.80	**0.04**	0	0	0	0	0	0.00	#DIV/0!	**0.04**
Imipenem	40	36	35	12	20	26	5.86	55	56	45	48	51	2.68	**0.02**	38	40	30	0	27	9.26	0.91	0.08
Meropenem	4	8	12	12	16	12	1.63	31	34	32	35	33	0.91	**0.00**	0	0	0	7	2	1.75	**0.01**	**0.00**
Meropenem	40	37	38	35	35	36	0.75	47	52	46	48	48	1.31	**0.00**	36	38	31	30	34	1.93	0.30	**0.00**
Nisin	4	0	7	7	7	5	1.75	9	10	10	9	10	0.29	0.09	7	7	7	8	7	0.25	0.34	**0.00**
Nisin	40	7	10	10	10	9	0.75	12	13	13	13	13	0.25	**0.01**	9	9	9	9	9	0.00	0.76	**0.00**
Ramoplanin	0.4	0	0	7	0	2	1.75	8	7	15	8	10	1.85	**0.02**	0	0	0	0	0	0.00	0.39	**0.01**
Ramoplanin	4	11	12	13	11	12	0.48	13	16	15	15	15	0.63	**0.01**	11	12	12	13	12	0.41	0.71	**0.01**
Ramoplanin	40	17	20	17	18	18	0.71	19	20	20	17	19	0.71	0.36	18	19	17	19	18	0.48	0.78	0.42
Rifampicin	4	0	0	0	0	0	0.00	24	21	21	20	22	0.87	**0.00**	0	0	7	0	2	1.75	0.39	**0.00**
Rifampicin	40	15	16	11	11	13	1.31	26	33	32	31	31	1.55	**0.00**	14	15	15	18	16	0.87	0.21	**0.00**
Teicoplanin	0.4	0	6	7	0	3	1.89	10	10	10	10	10	0.00	**0.04**	0	7	8	0	4	2.17	0.87	0.06
Teicoplanin	4	15	16	15	14	15	0.41	21	19	19	20	20	0.48	**0.00**	16	16	15	15	16	0.29	0.36	**0.00**
Teicoplanin	40	22	24	22	21	22	0.63	26	27	26	25	26	0.41	**0.00**	23	23	23	22	23	0.25	0.50	**0.00**
Tunicamycin	0.4	0	0	0	0	0	0.00	0	7	7	0	4	2.02	0.18	0	0	0	0	0	0.00	#DIV/0!	0.18
Tunicamycin	4	15	17	12	11	14	1.38	18	19	21	20	20	0.65	**0.02**	16	16	17	16	16	0.25	0.17	**0.01**
Tunicamycin	40	21	22	42	23	27	5.02	20	38	45	25	32	5.76	0.54	24	28	43	22	29	4.75	0.76	0.73
Vancomycin	0.4	0	0	0	0	0	0.00	7	11	12	0	8	2.72	0.07	0	0	0	0	0	0.00	#DIV/0!	0.07
Vancomycin	4	0	0	0	0	0	0.00	21	23	22	20	22	0.65	**0.00**	0	0	0	0	0	0.00	#DIV/0!	**0.00**
Vancomycin	40	0	0	0	0	0	0.00	30	28	28	28	29	0.50	**0.00**	0	0	0	0	0	0.00	#DIV/0!	**0.00**

		J1929						*DT1029 ppm1-*						Vs *J1929*	*DT1029 pDT16*						Vs *J1929*	Vs *DT1029*
**Antibiotic**	**conc (µg/disc)**	**1**	**2**	**3**	**4**	**AVERAGE**	**SEM**	**1**	**2**	**3**	**4**	**AVERAGE**	**SEM**	**P-value**	**1**	**2**	**3**	**4**	**AVERAGE**	**SEM**	**P-value**	**P-value**
Ampicillin	40	0	0	0	0	0	0.00	18	23	20	20	20	1.03	**0.00**	0	0	0	0	0	0.00	#DIV/0!	**0.00**
Bacitracin	4	11	12	8	9	10	0.91	15	16	13	13	14	0.75	**0.01**	0	12	11	10	8	2.78	0.59	0.12
Bacitracin	40	19	20	18	18	19	0.48	26	28	24	26	26	0.82	**0.00**	22	20	19	20	20	0.63	0.11	**0.00**
Daptamycin	40	0	0	0	0	0	0.00	25	21	21	17	21	1.63	**0.00**	0	0	0	0	0	0.00	#DIV/0!	**0.00**
Imipenem	4	0	0	0	0	0	0.00	38	40	12	17	27	7.16	**0.03**	0	0	0	0	0	0.00	#DIV/0!	**0.03**
Imipenem	40	36	35	12	20	26	5.86	58	65	48	46	54	4.44	**0.01**	43	45	31	0	30	10.39	0.75	0.09
Meropenem	4	8	12	12	16	12	1.63	35	42	34	36	37	1.80	**0.00**	10	0	12	10	8	2.71	0.26	**0.00**
Meropenem	40	37	38	35	35	36	0.75	50	60	50	50	53	2.50	**0.01**	37	39	33	35	36	1.29	0.87	**0.00**
Nisin	4	0	7	7	7	5	1.75	8	9	9	9	9	0.25	0.14	8	6	6	7	7	0.48	0.46	**0.02**
Nisin	40	7	10	10	10	9	0.75	11	12	12	13	12	0.41	**0.03**	9	9	9	10	9	0.25	1.00	**0.00**
Ramoplanin	0.4	0	0	7	0	2	1.75	8	10	9	0	7	2.29	0.14	0	7	0	0	2	1.75	1.00	0.14
Ramoplanin	4	11	12	13	11	12	0.48	16	16	15	16	16	0.25	**0.00**	14	12	12	12	13	0.50	0.32	**0.00**
Ramoplanin	40	17	20	17	18	18	0.71	18	23	18	20	20	1.18	0.26	19	18	17	19	18	0.48	0.78	0.31
Rifampicin	4	0	0	0	0	0	0.00	19	25	18	18	20	1.68	**0.00**	0	0	0	0	0	0.00	#DIV/0!	**0.00**
Rifampicin	40	15	16	11	11	13	1.31	31	36	30	33	33	1.32	**0.00**	15	20	12	13	15	1.78	0.46	**0.00**
Teicoplanin	0.4	0	6	7	0	3	1.89	11	9	10	10	10	0.41	**0.03**	8	0	9	9	7	2.18	0.30	0.21
Teicoplanin	4	15	16	15	14	15	0.41	18	16	18	15	17	0.75	0.10	15	19	17	18	17	0.85	0.07	0.68
Teicoplanin	40	22	24	22	21	22	0.63	25	22	22	25	24	0.87	0.29	25	23	24	23	24	0.48	0.11	0.81
Tunicamycin	0.4	0	0	0	0	0	0.00	0	0	0	8	2	2.00	0.39	0	0	0	0	0	0.00	#DIV/0!	0.39
Tunicamycin	4	15	17	12	11	14	1.38	20	23	21	20	21	0.71	**0.01**	17	19	14	18	17	1.08	0.12	**0.03**
Tunicamycin	40	21	22	42	23	27	5.02	25	32	31	25	28	1.89	0.83	23	37	47	23	33	5.85	0.50	0.53
Vancomycin	0.4	0	0	0	0	0	0.00	10	12	0	12	9	2.87	0.06	0	0	0	0	0	0.00	#DIV/0!	0.06
Vancomycin	4	0	0	0	0	0	0.00	21	21	18	21	20	0.75	**0.00**	0	0	0	0	0	0.00	#DIV/0!	**0.00**
Vancomycin	40	0	0	0	0	0	0.00	27	25	25	25	26	0.50	**0.00**	0	0	0	0	0	0.00	#DIV/0!	**0.00**

		J1929						*DT3017 ppm1-*						Vs *J1929*	*DT3017 pDT16*						Vs *J1929*	Vs *DT3017*
**Antibiotic**	**conc (µg/disc)**	**1**	**2**	**3**	**4**	**AVERAGE**	**SEM**	**1**	**2**	**3**	**4**	**AVERAGE**	**SEM**	**P-value**	**1**	**2**	**3**	**4**	**AVERAGE**	**SEM**	**P-value**	**P-value**
Ampicillin	40	0	0	0	0	0	0.00	24	20	24	25	23	1.11	**0.00**	0	0	0	0	0	0.00	#DIV/0!	**0.00**
Bacitracin	4	11	12	8	9	10	0.91	16	16	15	14	15	0.48	**0.00**	11	12	10	10	11	0.48	0.50	**0.00**
Bacitracin	40	19	20	18	18	19	0.48	27	27	27	26	27	0.25	**0.00**	23	21	20	20	21	0.71	**0.04**	**0.00**

Daptamycin	40	0	0	0	0	0	0.00	28	24	24	25	25	0.95	**0.00**	0	0	0		0	0.00	#DIV/0!	**0.00**
Imipenem	4	0	0	0	0	0	0.00	40	40	32	30	36	2.63	**0.00**	0	0	0	0	0	0.00	#DIV/0!	**0.00**
Imipenem	40	36	35	12	20	26	5.86	55	60	46	43	51	3.94	**0.01**	35	35	17	8	24	6.75	0.83	**0.02**
Meropenem	4	8	12	12	16	12	1.63	33	34	35	35	34	0.48	**0.00**	0	8	11	13	8	2.86	0.28	**0.00**
Meropenem	40	37	38	35	35	36	0.75	51	49	48	50	50	0.65	**0.00**	42	40	35	36	38	1.65	0.33	**0.00**
Nisin	4	0	7	7	7	5	1.75	8	9	9	10	9	0.41	0.12	8	7	7	8	8	0.29	0.29	**0.03**
Nisin	40	7	10	10	10	9	0.75	13	14	14	14	14	0.25	**0.01**	10	10	10	10	10	0.00	0.39	**0.00**
Ramoplanin	0.4	0	0	7	0	2	1.75	10	9	9	0	7	2.35	0.13	0	0	0	0	0	0.00	0.39	0.06
Ramoplanin	4	11	12	13	11	12	0.48	17	16	16	15	16	0.41	**0.00**	11	12	12	15	13	0.87	0.48	**0.02**
Ramoplanin	40	17	20	17	18	18	0.71	22	21	21	21	21	0.25	**0.01**	19	18	18	21	19	0.71	0.36	**0.04**
Rifampicin	4	0	0	0	0	0	0.00	20	20	20	20	20	0.00	#DIV/0!	0	0	0	0	0	0.00	#DIV/0!	#DIV/0!
Rifampicin	40	15	16	11	11	13	1.31	31	33	33	33	33	0.50	**0.00**	20	14	11	23	17	2.74	0.28	**0.01**
Teicoplanin	0.4	0	6	7	0	3	1.89	10	12	8	10	10	0.82	**0.03**	9	9	10	10	10	0.29	**0.04**	0.60
Teicoplanin	4	15	16	15	14	15	0.41	18	21	19	18	19	0.71	**0.00**	18	17	16	17	17	0.41	**0.01**	0.06
Teicoplanin	40	22	24	22	21	22	0.63	26	27	26	27	27	0.29	**0.00**	26	25	22	25	25	0.87	0.08	0.10
Tunicamycin	0.4	0	0	0	0	0	0.00	7	7	8	8	8	0.29	**0.00**	0	0	0	0	0	0.00	#DIV/0!	**0.00**
Tunicamycin	4	15	17	12	11	14	1.38	21	22	23	19	21	0.85	**0.01**	18	19	18	19	19	0.29	**0.04**	**0.04**
Tunicamycin	40	21	22	42	23	27	5.02	26	30	48	26	33	5.25	0.48	26	45	48	24	36	6.25	0.32	0.70
Vancomycin	0.4	0	0	0	0	0	0.00	10	14	12	13	12	0.85	**0.00**	0	0	0	0	0	0.00	#DIV/0!	**0.00**
Vancomycin	4	0	0	0	0	0	0.00	22	24	22	24	23	0.58	**0.00**	0	0	0	0	0	0.00	#DIV/0!	**0.00**
Vancomycin	40	0	0	0	0	0	0.00	30	28	30	30	30	0.50	**0.00**	0	0	0	0	0	0.00	#DIV/0!	**0.00**

		J1929						*DT2008 pmt-*						*Vs J1929*	*DT2008 pDT10*						*Vs J1929*	*Vs DT2008*
**Antibiotic**	**conc (µg/disc)**	**1**	**2**	**3**	**4**	**AVERAGE**	**SEM**	**1**	**2**	**3**	**4**	**AVERAGE**	**SEM**	**P-value**	**1**	**2**	**3**	**4**	**AVERAGE**	**SEM**	**P-value**	**P-value**
Ampicillin	80	15	16	9	7	12	2.21	23	21	18	19	20	1.11	**0.02**	16	15	9	7	12	2.21	1.00	**0.02**
Bacitracin	4	18	17	29	20	21	2.74	23	21	23	24	23	0.63	0.57	20	20	19	18	19	0.48	0.57	0.57
Bacitracin	40	30	17	28	30	26	3.12	31	21	31	46	32	5.15	0.37	28	20	28	28	26	2.00	0.95	0.37
Imipenem	4	0	0	0	0	0	0.00	20	19	23	25	22	1.38	**0.00**	0	0	0	0	0	0.00	#DIV/0!	**0.00**
Imipenem	40	42	30	33	30	34	2.84	50	41	45	46	46	1.85	**0.02**	40	24	28	28	30	3.46	0.44	**0.02**
Meropenem	40	15	9	34	0	15	7.19	24	26	38	25	28	3.28	0.15	0	0	26	0	7	6.50	0.44	0.15
Nisin	4	7	7	8	8	8	0.29	7	0	8	8	6	1.93	0.43	0	7	8	7	6	1.85	0.36	0.43
Nisin	40	10	10	10	10	10	0.00	10	10	11	11	11	0.29	0.18	10	10	10	10	10	0.00	#DIV/0!	0.18
Ramoplanin	4	18	14	14	14	15	1.00	23	14	15	16	17	2.04	0.42	20	14	14	14	16	1.50	0.79	0.42
Ramoplanin	40	30	20	20	20	23	2.50	31	20	20	23	24	2.60	0.79	28	21	20	21	23	1.85	1.00	0.79
Rifampicin	40	15	15	17	18	16	0.75	20	20	27	29	24	2.35	**0.04**	20	20	18	20	20	0.50	**0.01**	**0.04**
Teicoplanin	4	16	16	16	15	16	0.25	18	18	17	18	18	0.25	**0.00**	16	17	16	15	16	0.41	0.62	**0.00**
Teicoplanin	40	22	23	22	22	22	0.25	25	24	24	25	25	0.29	**0.00**	22	24	23	22	23	0.48	0.40	**0.00**
Tunicamycin	4	15	14	15	12	14	0.71	16	32	14	16	20	4.19	0.28	15	21	12	15	16	1.89	0.44	0.28
Tunicamycin	40	19	21	40	33	28	4.99	19	36	40	41	34	5.12	0.45	21	35	43	41	35	4.97	0.37	0.45
Vancomycin	40	0	8	0	0	2	2.00	10	13	12	12	12	0.63	**0.01**	0	8	0	7	4	2.17	0.58	**0.01**

		J1929						*DT1025 pmt-*						*Vs J1929*	*DT1025 pDT10*						*Vs J1929*	*Vs DT1025*
**Antibiotic**	**conc (µg/disc)**	**1**	**2**	**3**	**4**	**AVERAGE**	**SEM**	**1**	**2**	**3**	**4**	**AVERAGE**	**SEM**	**P-value**	**1**	**2**	**3**	**4**	**AVERAGE**	**SEM**	**P-value**	**P-value**
Ampicillin	80	22	23	23	21	22	0.48	29	29	29	25	28	1.00	**0.01**	22	22	22	21	22	0.25	0.40	**0.01**
Bacitracin	4	11	18	19	17	16	1.80	15	24	22	22	21	1.97	0.14	10	21	20	18	17	2.50	0.76	0.14
Bacitracin	40	20	28	29	27	26	2.04	24	32	33	31	30	2.04	0.22	19	30	30	29	27	2.68	0.78	0.22
Imipenem	4	0	0	0	0	0	0.00	32	30	17	15	24	4.37	**0.01**	0	0	0	0	0	0.00	#DIV/0!	**0.01**
Imipenem	40	37	34	25	24	30	3.24	48	50	42	40	45	2.38	**0.01**	33	30	20	20	26	3.38	0.40	**0.01**
Meropenem	40	27	27	26	27	27	0.25	38	37	35	37	37	0.63	**0.00**	27	25	25	26	26	0.48	0.13	**0.00**
Nisin	4	7	8	7	7	7	0.25	7	9	9	8	8	0.48	0.13	7	9	8	8	8	0.41	0.18	0.13
Nisin	40	10	9	10	9	10	0.29	10	10	12	12	11	0.58	0.07	10	10	10	10	10	0.00	0.18	0.07
Ramoplanin	4	12	12	14	13	13	0.48	14	15	15	15	15	0.25	**0.02**	13	13	14	12	13	0.41	0.71	**0.02**
Ramoplanin	40	20	18	21	19	20	0.65	20	20	22	20	21	0.50	0.27	19	18	20	18	19	0.48	0.39	0.27
Rifampicin	40	10	15	19	14	15	1.85	15	25	25	22	22	2.36	0.05	6	19	18	14	14	2.95	0.95	0.05
Teicoplanin	4	16	16	17	15	16	0.41	19	18	18	16	18	0.63	0.07	16	15	17	16	16	0.41	1.00	0.07
Teicoplanin	40	23	23	25	23	24	0.50	25	24	24	24	24	0.25	0.24	25	22	25	21	23	1.03	0.84	0.24
Tunicamycin	4	10	14	13	13	13	0.87	12	13	16	16	14	1.03	0.24	11	14	14	14	13	0.75	0.54	0.24
Tunicamycin	40	18	37	21	20	24	4.38	20	35	20	22	24	3.61	0.97	22	36	21	24	26	3.47	0.77	0.97
Vancomycin	40	9	7	0	0	4	2.35	20	17	15	14	17	1.32	**0.01**	10	7	0	0	4	2.53	0.94	**0.01**

Dataset S2; RT-PCR raw data

		Ct					
		hrdB			sigE		
Strain	Timpoint	A	B	C	A	B	C
J1929	0	18.799630	20.650000	21.454816	21.586304	24.590130	24.186640
J1929	30	19.581444	23.660000	20.810285	22.035706	27.638845	23.175257
J1929	60	19.978937	19.110000	20.519775	22.709986	22.212990	23.320545
J1929	90	20.026252	18.590000	21.138070	21.947371	21.624706	23.812683
DT3017	0	19.865672	20.277826	20.366220	23.448399	23.262208	23.075092
DT3017	30	21.201283	20.630247	20.216869	24.763846	23.714276	22.807042
DT3017	60	20.747127	21.744322	21.703077	23.967405	25.427483	25.351522
DT3017	90	21.062787	21.567923	20.694414	24.580005	25.288694	23.855792
DT3017pDT16	0	20.527110	22.274062	20.908951	24.340387	25.227647	24.092606
DT3017pDT16	30	20.731414	23.753307	21.081755	24.288817	26.961072	24.056971
DT3017pDT16	60	21.233016	21.756851	20.932545	24.536002	24.266238	23.999046
DT3017pDT16	90	21.658429	20.026058	20.660345	24.698671	22.467578	23.170101
DT2008	0	21.553286	20.571101	19.820496	26.340847	26.255304	25.973239
DT2008	30	20.809232	19.534068	19.895908	26.176449	24.638397	25.580193
DT2008	60	21.621939	21.673930	20.498451	24.718257	25.050780	26.486415
DT2008	90	20.848227	21.170345	20.352083	25.562053	23.913292	26.416597
DT2008pDT10	0	20.604540	22.577943	20.941118	26.006975	24.979057	24.884230
DT2008pDT10	30	21.918685	19.754117	20.233721	23.995457	23.747550	24.766716
DT2008pDT10	60	20.382804	20.032450	21.274306	25.829701	26.071708	24.279032
DT2008pDT10	90	20.897380	18.734000	21.068324	24.963149	24.430338	24.189709

			delta Ct						delta Ct	
vanH			sigE			vanH			sigE	
A	B	C	A	B	C	A	B	C	AVERAGE	SEM
30.002860	31.654559	32.280459	2.786674	3.940130	2.731825	11.203229	11.004559	10.825644	3.152876	0.393945
22.645180	29.102357	23.006241	2.454262	3.978845	2.364972	3.063736	5.442357	2.195956	2.932693	0.523710
23.616922	25.818885	24.868385	2.731049	3.102990	2.800770	3.637986	6.708885	4.348610	2.878270	0.114149
26.429050	26.584279	30.017014	1.921118	3.034706	2.674613	6.402798	7.994279	8.878943	2.543479	0.328083
31.197264	30.991518	30.474890	3.582727	2.984381	2.708872	11.331591	10.713692	10.108669	3.091993	0.257935
24.671139	29.624201	27.444438	3.562563	3.084029	2.590172	3.469856	8.993954	7.227568	3.078921	0.280717
23.249606	31.000706	29.374309	3.220278	3.683160	3.648445	2.502479	9.256384	7.671232	3.517294	0.148846
26.725033	32.216434	30.198291	3.517218	3.720771	3.161378	5.662246	10.648511	9.503878	3.466456	0.163465
31.073481	33.990208	32.113710	3.813277	2.953584	3.183655	10.546370	11.716145	11.204759	3.316839	0.256951
23.102547	30.039613	25.842295	3.557404	3.207766	2.975216	2.371134	6.286306	4.760540	3.246795	0.169192
24.130721	31.840541	28.976849	3.302986	2.509387	3.066500	2.897706	10.083689	8.044303	2.959624	0.235242
26.894819	27.536483	30.405720	3.040242	2.441521	2.509755	5.236390	7.510426	9.745375	2.663839	0.189229
34.868998	33.004412	31.097172	4.787561	5.684203	6.152743	13.315713	12.433311	11.276676	5.541503	0.400501
22.589537	24.661365	23.365168	5.367217	5.104329	5.684285	1.780305	5.127297	3.469261	5.385277	0.167662
23.948778	28.534428	23.711927	3.096317	3.376850	5.987964	2.326839	6.860498	3.213476	4.153710	0.920695
25.956992	28.649881	24.925369	4.713826	2.742947	6.064514	5.108766	7.479537	4.573286	4.507096	0.964409
33.495525	32.853308	31.452630	5.402435	2.401114	3.943112	12.890985	10.275365	10.511512	3.915554	0.866517
31.483201	28.127752	28.104384	2.076772	3.993434	4.532995	9.564516	8.373635	7.870663	3.534400	0.745272
31.703098	29.328746	30.740419	5.446898	6.039258	3.004726	11.320295	9.296295	9.466113	4.830294	0.928663
31.610954	27.096535	30.927499	4.065769	5.696338	3.121385	10.713573	8.362535	9.859175	4.294497	0.752071

average		delta delta Ct						2-de		
vanH		sigE			vanH			sigE		
AVERAGE	SEM	A	B	C	A	B	C	A	B	C
11.011144	0.109049	0.000000	0.000000	0.000000	0.000000	0.000000	0.000000	1.000000	1.000000	1.000000
3.567350	0.970395	-0.332411	0.038715	-0.366852	-8.139493	-5.562202	-8.629688	1.259116	0.973522	1.289536
4.898493	0.928149	-0.055625	-0.837139	0.068945	-7.565243	-4.295675	-6.477034	1.039309	1.786504	0.953335
7.758674	0.724444	-0.865555	-0.905424	-0.057212	-4.800431	-3.010280	-1.946700	1.822041	1.873095	1.040453
10.717984	0.353034	0.796053	-0.955748	-0.022953	0.128362	-0.290868	-0.716974	0.575923	1.939586	1.016037
6.563793	1.628841	0.775889	-0.856101	-0.141652	-7.733374	-2.010605	-3.598075	0.584029	1.810140	1.103168
6.476698	2.039117	0.433604	-0.256970	0.916620	-8.700750	-1.748176	-3.154411	0.740410	1.194966	0.529749
8.604878	1.507963	0.730544	-0.219359	0.429553	-5.540983	-0.356049	-1.321766	0.602676	1.164216	0.742492
11.155758	0.338573	1.026603	-0.986546	0.451831	-0.656859	0.711586	0.379115	0.490865	1.981435	0.731115
4.472660	1.139342	0.770730	-0.732364	0.243391	-8.832095	-4.718253	-6.065104	0.586121	1.661359	0.844757
7.008566	2.138080	0.516312	-1.430743	0.334675	-8.305524	-0.920870	-2.781340	0.699157	2.695855	0.792962
7.497397	1.301648	0.253568	-1.498609	-0.222069	-5.966839	-3.494134	-1.080268	0.838819	2.825702	1.166405
12.341900	0.590391	2.000888	1.744074	3.420918	2.112483	1.428751	0.451032	0.249846	0.298526	0.093369
3.458954	0.966207	2.580543	1.164199	2.952460	-9.422924	-5.877262	-7.356383	0.167178	0.446212	0.129188
4.133604	1.387263	0.309643	-0.563280	3.256139	-8.876390	-4.144061	-7.612167	0.806841	1.477625	0.104666
5.720529	0.892985	1.927153	-1.197183	3.332689	-6.094464	-3.525023	-6.252357	0.262948	2.292915	0.099257
11.225954	0.835302	2.615761	-1.539016	1.211287	1.687756	-0.729195	-0.314131	0.163146	2.905963	0.431883
8.602938	0.502235	-0.709902	0.053304	1.801170	-1.638713	-2.630924	-2.954981	1.635693	0.963727	0.286942
10.027568	0.648220	2.660224	2.099128	0.272902	0.117065	-1.708264	-1.359531	0.158195	0.233399	0.827653
9.645095	0.687076	1.279095	1.756208	0.389560	-0.489656	-2.642025	-0.966468	0.412054	0.296025	0.763362

lta delta Ct			2-delta delta Ct Average						
vanH			sigE		vanH				sigE
A	B	C	Average	SEM	AVERAGE	SEM			A
1.000000	1.000000	1.000000	1.000000	0.000000	1.000000	0.000000		J1929	0.000000
281.988654	47.248683	396.090867	1.174058	0.100652	241.776068	102.689672		J1929	0.100066
189.393552	19.639345	89.080245	1.259716	0.264561	99.371047	49.273212		J1929	0.016745
27.865940	8.057208	3.854919	1.578530	0.269442	13.259356	7.403356		J1929	0.260558
0.914870	1.223376	1.643731	1.177182	0.401817	1.260659	0.211228		DT3017	-0.239636
212.802847	4.029512	12.109566	1.165779	0.355329	76.313975	68.284286		DT3017	-0.233566
416.089581	3.359335	8.903739	0.821708	0.196287	142.784218	136.662054		DT3017	-0.130528
46.558840	1.279916	2.499719	0.836461	0.168774	16.779491	14.893837		DT3017	-0.219916
1.576646	0.610649	0.768909	1.067805	0.462050	0.985401	0.299132		pDT16	-0.309038
455.748932	26.323019	66.954241	1.030746	0.324026	183.008731	136.873588		pDT16	-0.232013
316.381966	1.893257	6.874907	1.395992	0.650496	108.383377	104.009237		pDT16	-0.155425
62.545729	11.267799	2.114429	1.610309	0.615010	25.309319	18.804775		pDT16	-0.076332
0.231249	0.371452	0.731519	0.213913	0.061889	0.444740	0.148992		DT2008	-0.602327
686.408707	58.780363	163.867167	0.247526	0.099947	303.018746	194.080487		DT2008	-0.776821
469.958795	17.680180	195.654890	0.796377	0.396374	227.764621	131.545002		DT2008	-0.093212
68.330779	11.511650	76.233726	0.885040	0.705522	52.025385	20.384930		DT2008	-0.580131
0.310409	1.657714	1.243263	1.166997	0.872937	1.070462	0.398415		pDT10	-0.787423
3.113879	6.194228	7.754216	0.962120	0.389352	5.687441	1.363306		pDT10	0.213702
0.922061	3.267675	2.566017	0.406416	0.211735	2.251918	0.695095		pDT10	-0.800807
1.404110	6.242070	1.954051	0.490481	0.140492	3.200077	1.529259		pDT10	-0.385046

Log fold change					Log fold change Average			
		vanH			sigE		vanH	
B	C	A	B	C	Average	SEM	AVERAGE	SEM
0.000000	0.000000	0.000000	0.000000	0.000000	0.000000	0.000000	0.000000	0.000000
-0.011654	0.110434	2.450232	1.674390	2.597795	0.066282	0.039083	2.240805	0.286394
0.252004	-0.020754	2.277365	1.293127	1.949781	0.082665	0.085359	1.840091	0.289370
0.272560	0.017223	1.445074	0.906185	0.586015	0.183447	0.083184	0.979091	0.250654
0.287709	0.006910	-0.038641	0.087560	0.215831	0.018328	0.152338	0.088250	0.073460
0.257712	0.042642	2.327977	0.605252	1.083129	0.022263	0.142185	1.338786	0.513474
0.077356	-0.275930	2.619187	0.526253	0.949572	-0.109701	0.102515	1.365004	0.638887
0.066034	-0.129308	1.668002	0.107181	0.397891	-0.094397	0.084372	0.724358	0.479227
0.296980	-0.136015	0.197734	-0.214209	-0.114125	-0.049358	0.180228	-0.043533	0.124045
0.220464	-0.073268	2.658726	1.420336	1.825778	-0.028272	0.132542	1.968280	0.364524
0.430697	-0.100747	2.500212	0.277210	0.837267	0.058175	0.186929	1.204896	0.667532
0.451126	0.066850	1.796198	1.051839	0.325193	0.147215	0.157477	1.057743	0.424653
-0.525018	-1.029799	-0.635921	-0.430097	-0.135774	-0.719048	0.156970	-0.400597	0.145131
-0.350459	-0.888779	2.836583	1.769232	2.214492	-0.672020	0.163997	2.273436	0.309524
0.169564	-0.980196	2.672060	1.247487	2.291491	-0.301281	0.347830	2.070346	0.425845
0.360388	-1.003239	1.834616	1.061138	1.882147	-0.407661	0.402980	1.592634	0.266102
0.463290	-0.364634	-0.508065	0.219510	0.094563	-0.229589	0.367309	-0.064664	0.224615
-0.016046	-0.542206	0.493302	0.791987	0.889538	-0.114850	0.223734	0.724942	0.119195
-0.631900	-0.082152	-0.035240	0.514239	0.409260	-0.504953	0.216951	0.296086	0.168412
-0.528671	-0.117269	0.147401	0.795329	0.290936	-0.343662	0.120551	0.411222	0.196472

Dataset S3: Gene for GO analysis

	DT3017 : J1929					
**Gene**	log2 FC	p-value	GeneID	Symbol	Aliases	description
**SCO3737**	5.22732857	7.15E-47	1099173	SCO3737	SCO3737SCH22A.15c	lipoprotein, putative Sortase E
**SCO3738**	5.01876726	3.18E-14	1099174	SCO3738	SCO3738SCH22A.16c	hypothetical protein
**SCO6183**	4.04646883	0.03337329	1101624	SCO6183	SCO6183SC2G5.04 cwgE	transferase part of cwg operon sco6179-6190, GT1 heptosyl-transferase
**SCO3736**	3.75595274	2.06E-20	1099172	SCO3736	SCO3736SCH22A.14c	RNA polymerase ECF sigma factor
**SCO5525**	3.02459348	0.01739439	1100965	SCO5525	SCO5525 ureAB	bifunctional urease subunit gamma/beta
**SCO5535**	2.49833124	1.43E-05	1100975	SCO5535	SCO5535SC1C2.16 accB	carboxyl transferase
**SCO4175**	2.45797758	0.01910115	1099615	SCO4175	SCO4175SCD66.12c	hypothetical protein
**SCO2414**	2.34375333	0.01646835	1097848	SCO2414	SCO2414SC8A2.02c mce	hypothetical protein, possibly part of Mce protein complex
**SCO7415**	2.33829765	0.00423036	1102853	SCO7415	SCO7415SC6D11.11	racemase
**SCO3706**	2.24156167	6.43E-07	1099142	SCO3706	SCO3706SCH35.18c	ABC transporter ATP-binding protein
**SCO2387**	2.15966885	1.68E-06	1097821	SCO2387	SCO2387SC4A7.15, fabD	ACP S-malonyltransferase
**SCO2472**	2.07413879	0.0185831	1097906	SCO2472	SCO2472SC7A8.11	hypothetical protein secreted, SanA
**SCO3707**	2.0308984	2.37E-06	1099143	SCO3707	SCO3707SCH35.17	lipoprotein
**SCO1815**	1.91707253	2.30E-05	1097249	SCO1815	SCO1815SCI28.09c, fabG	3-oxacyl-ACP reductase
**SCO1335**	1.91485495	0.00349242	1096758	SCO1335	SCO13352SCG61.17c	oxidoreductase
**SCO2026**	1.90640417	0.01568253	1097460	SCO2026	SCO2026SC3A3.04c, gltB	glutamate synthase
**SCO5526**	1.89953742	0.00261889	1100966	ureC	SCO5526	urease subunit alpha
**SCO1909**	1.8639413	0.0012721	1097343	SCO1909	SCO1909SCI7.27c	hypothetical protein
**SCO1814**	1.83510828	7.85E-05	1097248	SCO1814	SCO1814SCI28.08c, fabI	enoyl-ACP reductase
**SCO5950**	1.81382462	0.0292013	1101392	SCO5950	SCO5950SC7H1.20c	export protein, drug resistance transporter EmrB/QacA subfamily
**SCO5092**	1.77342064	0.04872546	1100533	SCO5092	SCO5092SCBAC28G1.18, actVB	actinorhodin polyketide dimerase
**SCO5527**	1.76300958	0.00632636	1100967	SCO5527	SCO5527SC1C2.08	hypothetical protein, putative Peptidyl-arginine deiminase (PAD)
**SCO5515**	1.75904007	9.90E-06	1100955	SCO5515	SCO5515SC8D9.27, serA	D-3-phosphoglycerate dehydrogenase
**SCO2025**	1.74066765	0.00900683	1097459	gltD	SCO2025SC3A3.03c	glutamate synthase
**SCO7036**	1.69428699	5.97E-05	1102474	SCO7036	SCO7036SC4G1.02, argG	argininosuccinate synthase
**SCO6999**	1.68886923	0.04785862	1102437	SCO6999	SCO6999SC8F11.25c.	hypothetical protein, putative HutG, N-formylglutamate amidohydrolase
**SCO1233**	1.68478024	0.00332918	1096656	SCO1233	SCO12332SCG1.08c, ureF	urease accessroy protein UreF
**SCO3656**	1.68323618	0.00030916	1099092	SCO3656	SCO3656SCH10.34c	hypothetical protein
**SCO2388**	1.67143723	3.75E-05	1097822	SCO2388	SCO2388SC4A7.16, fabH	3-oxoacyl-ACP synthase
**SCO1182**	1.66368654	1.09E-07	1096605	SCO1182	SCO1182SCG11A.13	hypothetical protein
**SCO2481**	1.63093372	0.0487074	1097915	SCO2481	SCO2481SC7A8.20c	hypothetical protein, putative regulator
**SCO3104**	1.5777876	0.02384028	1098538	SCO3104	SCO3104SCE41.13c	hypothetical protein
**SCO6564**	1.56557775	0.0002604	1102003	SCO6564	SCO6564SC4B5.14, fabH2	3-oxoacyl-ACP synthase
**SCO1342**	1.54547041	0.00054352	1096765	SCO1342	SCO13422SCG61.24c	hypothetical protein
**SCO3207**	1.52575574	0.00203127	1098641	SCO3207	SCO3207SCE22.24c	**TetR family transcriptional regulator**
**SCO4914**	1.52406327	0.04451133	1100355	SCO4914	SCO4914SCK13.06c	deoxyribose-phosphate aldolase
**SCO1577**	1.52073619	0.01257825	1097008	argD	SCO1577SCL24.13c argD	acetylornithine aminotransferase
**SCO5516**	1.51468065	0.00013034	1100956	SCO5516	SCO5516SC8D9.28c	integral membrane efflux protein, EmrB/QacA family drug efflux
**SCO7268**	1.50978732	0.00888465	1102706	SCO7268	SCO7268SC5H1.24c, add	adenosine deaminase
**SCO3127**	1.44487769	0.00144788	1098561	SCO3127	SCO3127SCE66.06c, ppc	phosphoenolpyruvate carboxylase
**SCO4913**	1.43999556	0.02698643	1100354	SCO4913	SCO4913SCK13.05c	aldehyde dehydrogenase
**SCO3771**	1.43601259	0.03782396	1099207	SCO3771	SCO3771SCH63.18c	penicillin binding protein, FtsI like/ PBP2
**SCO3379**	1.42736306	0.03915888	1098816	SCO3379	SCO3379SCE94.30c	hypothetical protein
**SCO1908**	1.42242332	0.0022921	1097342	SCO1908	SCO1908SCI7.26	large hypothetical protein, putative extracelluar nuclease
**SCOr18**	1.41525103	0.03274651	1101187	rrnE	SCOr18	5S ribosomal RNA

**SCO5554**	1.4041096	0.02526811	1100994	leuD	SCO5554	isopropylmalate isomerase small subunit
**SCO4142**	1.39513004	0.01836659	1099582	SCO4142	SCO4142SCD84.09c, pstS	phosphate-binding protein
**SCO2391**	1.39182774	0.0003971	1097825	SCO2391	SCO2391SC4A7.19c	hypothetical protein
**SCO4912**	1.39003632	0.02110597	1100353	SCO4912	SCO4912SCK13.04c	aldehyde dehydrogenase
**SCO6172**	1.36834937	0.002008	1101613	SCO6172	SCO6172SC6C5.08	oxidoreductase
**SCO1580**	1.36247597	0.00578432	1097011	argC	SCO1580	N-acetyl-gamma-glutamyl-phosphate reductase
**SCO4239**	1.35956269	0.04525137	1099679	SCO4239	SCO4239SCD8A.12c	small membrane protein
**SCO1579**	1.35398966	0.00398484	1097010	argJ	SCO1579SCL24.15c argJ	bifunctional ornithine acetyltransferase/N-acetylglutamate synthase
**SCO6804**	1.34946767	0.00096466	1102243	SCO6804	SCO6804SC1A2.13	hypothetical protein
**SCO7724**	1.34579418	0.04097975	1103162	SCO7724	SCO7724SC8D11.15c	hypothetical protein
**SCO4049**	1.33545477	0.02645228	1099485	SCO4049	SCO40492SCD60.15	antibiotic binding protein, Penicillin amidase or penicillin acylase
**SCO7705**	1.33398042	0.00974989	1103143	SCO7705	SCO7705SCBAC12C8.06	oxidoreductase
**SCO1334**	1.33242233	0.00769503	1096757	SCO1334	SCO13342SCG61.16	hypothetical protein
**SCO6271**	1.28937357	0.00138585	1101712	SCO6271	SCO6271SC2C4.01c, SCAH10.36c, acc	acyl-CoA carboxylase complex A subunit
**SCO1570**	1.28882089	0.00365482	1097001	SCO1570	SCO1570SCL24.06c, argH	argininosuccinate lyase
**SCO4891**	1.27425517	0.03088234	1100332	SCO4891	SCO48912SCK8.17c	hypothetical protein possibly secreted
**SCO1244**	1.26977039	0.0009418	1096667	SCO1244	SCO12442SCG1.19, bioB	biotin synthase
**SCO2630**	1.26129089	0.01042897	1098064	SCO2630	SCO2630SC8E4.05c	biotin synthase
**SCO3206**	1.2612665	0.0023635	1098640	SCO3206	SCO3206SCE22.23c	transmembrane efflux protein, EmrB/QacA subfamily
**SCO2210**	1.25974103	0.01352136	1097643	SCO2210	SCO2210SC10B7.05, glnII	glutamine synthetase
**SCO1846**	1.25526263	0.01628379	1097280	SCO1846	SCO1846SCI8.31	hypothetical protein
**SCO5184**	1.25323458	0.01451459	1100625	SCO5184	SCO51842SC3B6.08	ATP-dependent DNA helicase
**SCO4369**	1.23752475	0.0273341	1099809	SCO4369	SCO43692SCD52.01, SCD10.01	hypothetical protein
**SCO6347**	1.22874848	0.03018267	1101788	SCO6347	SCO6347SC3A7.15	beta-galactosidase
**SCOt33**	1.22595676	0.02414746	3240036	SCOt33	SCOt33	tRNA
**SCO5584**	1.22589266	0.04645387	1101025	SCO5584	SCO5584SC7A1.28, glnB	nitrogen regulatory protein P-II, GlnK
**SCO0756**	1.22576664	0.04168175	1096179	SCO0756	SCO0756SCF81.15	ABC transporter, part of a lantibiotic/bacteriocin export system
**SCO1343**	1.22540857	0.00163953	1096766	SCO1343	SCO13432SCG61.25c, ung	uracil-DNA glycosylase
**SCO4921**	1.2243441	5.85E-05	1100362	SCO4921	SCO4921SCK13.13c, accA2	acyl-CoA carboxylase complex A subunit
**SCO3280**	1.21950295	0.02431245	1098714	SCO3280	SCO3280SCE39.30	hypothetical protein
**SCO4107**	1.21897177	0.00029895	1099544	SCO4107	SCO4107SCD17.11	hypothetical protein, serine phosphatase SpoIIE like
**SCO5553**	1.21710062	0.01090225	1100993	SCO5553	SCO5553leuC	isopropylmalate isomerase large subunit
**SCO2014**	1.21258777	1.55E-05	1097448	SCO2014	SCO2014SC7H2.28c, pyk1	pyruvate kinase
**SCO5522**	1.21184626	0.03700166	1100962	SCO5522	SCO5522leuB	3-isopropylmalate dehydrogenase
**SCO2810**	1.20592847	0.03404994	1098244	SCO2810	SCO28102SCC13.18, SCBAC17F8.01	hypothetical protein, putative helicase
**SCO3383**	1.20443628	0.00130087	1098820	panC	SCO3383SCE126.01c, SCE94.34c	pantoate--beta-alanine ligase
**SCO2228**	1.20391017	0.02180695	1097661	SCO2228	SCO2228SC10B7.23c, aglA	alpha-glucosidase
**SCO1487**	1.20013849	0.02369211	1096913	pyrB	SCO1487SC9C5.11c pyrB	aspartate carbamoyltransferase catalytic subunit
**SCO5391**	1.1955678	0.00884508	1100831	SCO5391	SCO53912SC6G5.35	ATP/GTP-binding protein
**SCO5106**	1.1770594	0.00533819	1100547	SCO5106	SCO5106SCBAC31E11.02c, shdB2	fumarate reductase iron-sulfur subunit
**SCO4901**	1.12828194	0.02954604	1100342	SCO4901	SCO49012SCK8.27	adenosine deaminase
**SCO2230**	1.12470606	0.04729329	1097663	SCO2230	SCO2230SC10B7.25c, malF	maltose permease
**SCO5425**	1.11431344	0.0062404	1100865	SCO5425	SCO5425SC6A11.01c, SC8F4.29c, pta	phosphate acetyltransferase
**SCO6565**	1.11076799	0.00126016	1102004	SCO6565	SCO6565SC4B5.15	**transcriptional regulator**
**SCOt31**	1.10861535	0.00228837	3240034	SCOt31	SCOt31	tRNA
**SCO6173**	1.10783534	0.00787534	1101614	SCO6173	SCO6173SC6C5.09	permease SC6C509, putative xanthin/uracil/vitC uptake
**SCO5860**	1.10288406	0.01083112	1101302	SCO5860	SCO5860SC9B10.27, suhB	SuhB protein, inositol monophosphatase
**SCO1742**	1.08977573	0.01638626	1097173	SCO1742	SCO1742SCI11.31	ABC transporter ATP-binding protein, part of a putative bacitracin resistance efflux system.

**SCO1232**	1.08518138	0.01057889	1096655	SCO1232	SCO12322SCG1.07c, ureG	urease accessory protein
**SCO4637**	1.08005808	0.01441786				FabA subunit 3-hydroxyacyl-ACP dehydratase
**SCO3964**	1.07969242	0.01713679	1099400	SCO3964	SCO3964SCBAC25E3.01c, SCD78.31c	hypothetical protein, putative copper export protein efflux
**SCO1245**	1.05843852	0.01101014	1096668	SCO1245	SCO12452SCG1.20, bioA	adenosylmethionine-8-amino-7-oxononanoate aminotransferase
**SCO5797**	1.05718402	0.00902066	1101239	SCO5797	SCO5797SC4H2.18c	hypothetical protein lipoprotein
**SCO5423**	1.05629964	0.03764574	1100863	SCO5423	SCO5423SC8F4.27c, pyk2	pyruvate kinase
**SCO5609**	1.05282785	0.00209643	1101050	SCO5609	SCO5609SC2E1.26c	hypothetical protein
**SCO5467**	1.05020555	0.00601183	1100907	SCO5467	SCO5467SC2A11.01, SC3D11.24	muramoyl-pentapeptide carboxypeptidase
**SCO3345**	1.047996	0.02035022	1098782	SCO3345	SCO3345SCE7.12c	dihydroxy-acid dehydratase
**SCO3310**	1.04736819	0.02343298	1098744	SCO3310	SCO3310SCE68.08	hypothetical protein
**SCO4159**	1.04450919	0.04076035	1099599	SCO4159	SCO4159SCD84.26c, glnR	**transcriptional regulator GlnR**
**SCO0030**	1.04322879	0.00725767	1095463	SCO0030	SCO0030SCJ4.11	hypothetical protein
**SCO1243**	1.04033225	0.01963125	1096666	SCO1243	SCO12432SCG1.18c, bioF	8-amino-7-oxononanoate synthase
**SCO4445**	1.03570115	0.01228103	1099885	SCO4445	SCO4445SCD6.23c	**transcriptional regulator**
**SCO2961**	1.03204411	0.00254762	1098394	SCO2961	SCO2961SCE59.20c	hypothetical protein, putative acyltransferase
**SCO2232**	1.02767584	0.00594784	1097665	SCO2232	SCO2232SC10B7.27, malR	maltose operon transcriptional repressor
**SCO1486**	1.0256003	0.03351964	1096912	pyrC	SCO1486SC9C5.10c	dihydroorotase
**SCO2093**	1.02062894	0.01268068	1097527	SCO2093	SCO2093SC4A10.26	hypothetical protein
**SCO1662**	1.02036049	0.01032877	1097093	SCO1662	SCO1662SCI52.04	hypothetical protein, proteosome assembly chaperone
**SCO2048**	1.01788602	0.0089681	1097482	SCO2048	SCO2048SC4G6.17c	imidazole glycerol phosphate synthase subunit HisF
**SCO3058**	1.0141049	0.03331063	1098491	SCO3058	SCO3058SCBAC19G2.13c	membrane dipeptidase
**SCO3311**	1.01250948	0.00625896	1098745	SCO3311	SCO3311SCE68.09c, hemB	delta-aminolevulinic acid dehydratase
**SCO7020**	1.00575796	0.0115876	1102458	SCO7020	SCO7020amlB	alpha-amylase, secreted
**SCO1813**	1.00534147	0.0131562	1097247	SCO1813	SCO1813SCI28.07	**GntR family transcriptional regulator**
**SCO3756**	1.00096581	0.02969241	1099192	SCO3756	SCO3756SCH63.03c	two component system response regulator
**SCO5839**	0.99455399	0.0498894	1101281	SCO5839	SCO5839SC9B10.06	hypothetical protein, putative metalloendopeptidase
**SCO0870**	0.99411984	0.01418603	1096293	SCO0870	SCO0870SCM1.03c	**two-component system response regulator**
**SCO1578**	0.99121965	0.01200289	1097009	SCO1578	SCO1578SCL24.14c, argB	acetylglutamate kinase
**SCOt46**	0.97294247	0.04112216	3240049	SCOt46	SCOt46	tRNA
**SCO1922**	0.96992786	0.0206485	1097356	SCO1922	SCO1922SCC22.04c	ABC transporter ATP-binding protein, part of SUF system; acts in Fe-S cluster formation in oxidative stress
**SCO3450**	0.95810869	0.03356476	1098887	SCO3450	SCO3450SCE46.07c	**ECF subfamily RNA polymerase sigma factor**
**SCO4962**	0.94620683	0.04195971	1100403	SCO4962	SCO49622SCK31.22	threonine dehydratase
**SCO3309**	0.93780531	0.02314469	1098743	SCO3309	SCO3309SCE68.07c	hypothetical protein, putative cell division protein ZipA
**SCO1214**	0.92591595	0.01497731	1096637	SCO1214	SCO12142SCG58.14, pfkA3	6-phosphofructokinase
**SCO1490**	0.92540503	0.04266022	1096916	nusB	SCO1490SC9C5.14c	transcription antitermination protein NusB
**SCO4038**	0.92218709	0.03334874	1099474	SCO4038	SCO40382SCD60.04c	putative cyctosine/adenosie deaminase
**SCO0280**	0.92204628	0.03001684	1095704	SCO0280	SCO0280SCF85.08c	hypothetical protein, putative GDP-mannose pyrophosphatase NudK
**SCO4636**	0.91782728	0.0489006	1100077	SCO4636	SCO4636SCD82.07	Putative (3R)-hydroxyacyl-ACP dehydratase subunit HadA; FabA (Singh and Reynolds 2016)
**SCO5289**	0.8818767	0.03172312	1100730	SCO5289	SCO5289SC6G9.44c, SCCB12.13, cvnA	**two component sensor kinase**
**SCO4464**	0.87878623	0.01946299	1099904	SCO4464	SCO4464SCD65.07c	hydrolase
**SCO7422**	0.87566319	0.03686061	1102860	SCO7422	SCO7422SC6D11.18c, cvnA10	**sensor histidine-kinase**
**SCOt62**	0.87500337	0.03832349	3240065	SCOt62	SCOt62	tRNA
**SCO5290**	0.86676088	0.03748657	1100731	SCO5290	SCO5290SC6G9.43c, cvnB5	hypothetical protein
**SCO2094**	0.86673866	0.00403334	1097528	SCO2094	SCO2094SC4A10.27	**regulatory protein**
**SCO1938**	0.86111753	0.02722785	1097372	SCO1938	SCO1938SCC22.20	hypothetical protein, putative glucose-6-phosphate dehydrogenase assembly protein OpcA
**SCO1483**	0.86075683	0.03945893	1096909	carB	SCO1483SC9C5.07c, pyrA	carbamoyl phosphate synthase large subunit
**SCO3059**	0.85872702	0.03255055	1098492	SCO3059	SCO3059SCBAC19G2.14c, purE	phosphoribosylaminoimidazole carboxylase catalytic subunit PurE
**SCOt37**	0.85344638	0.02425942	3240040	SCOt37	SCOt37	tRNA

**SCO5291**	0.84747199	0.04778846	1100732	SCO5291	SCO5291SC6G9.42c, cvnC5	hypothetical protein
**SCO2457**	0.84069294	0.01390485	1097891	SCO2457	SCO2457SCC24.28c	lipoprotein
**SCO5424**	0.83296057	0.01745499	1100864	SCO5424	SCO5424SC8F4.28c, ackA	acetate kinase
**SCO0871**	0.83212333	0.0045943	1096294	SCO0871	SCO0871SCM1.04c	**two-component sensor protein**
**SCO2390**	0.83131564	0.02491241	1097824	SCO2390	SCO2390SC4A7.18, fabF	3-oxoacyl-ACP synthase, FabF/FAbB
**SCO5583**	0.82348181	0.01692363	1101024	SCO5583	SCO5583SC7A1.27	ammonium transporter
**SCO7219**	0.82254635	0.0313864	1102657	SCO7219	SCO7219SC2H12.18c	phosphoglycerate mutase
**SCO7465**	0.82195193	0.00570404	1102903	SCO7465	SCO7465SCBAC14E8.05, cvnC13	hypothetical protein
**SCO3382**	0.81082749	0.01351651	1098819	SCO3382	SCO3382SCE94.33c, nadB	L-aspartate oxidase
**SCO0872**	0.8099131	0.02929893	1096295	SCO0872	SCO0872SCM1.05c	hypothetical protein, regulator putative serine phosphatase
**SCO7466**	0.80662976	0.03643229	1102904	SCO7466	SCO7466SCBAC14E8.06, cvnD13	ATP/GTP-binding protein
**SCO7439**	0.80537378	0.03456663	1102877	SCO7439	SCO7439SC6D11.35	hypothetical protein, putative Type 1 glutamine amidotransferase (GATase1)-like domain
**SCO4510**	0.80001471	0.01144239	1099950	SCO4510	SCO4510SCD35.17c	hypothetical protein
**SCO0249**	0.79006137	0.02272439	1095673	SCO0249	SCO0249SCJ9A.28c	hypothetical protein
**SCO5732**	0.78854428	0.01261973	1101171	SCO5732	SCO5732SC3C3.18c	hypothetical protein
**SCO0461**	0.77262507	0.0497584	1095884	SCO0461	SCO0461SCF51A.39, SCF76.01	hydrolase MhpC like
**SCO1946**	0.77134417	0.02375494	1097380	pgk	SCO1946SCC54.06c	phosphoglycerate kinase
**SCO5220**	0.76023795	0.01074036	1100661	SCO5220	SCO5220SC7E4.17	hypothetical protein
**SCO5145**	0.73991642	0.04089665	1100586	SCO5145	SCO5145SCP8.08	hypothetical protein
**SCO5564**	0.72522182	0.02820353	1101005	rpmB	SCO5564SC7A1.08c	50S ribosomal protein L28
**SCO5719**	0.70566034	0.04573059	1101158	SCO5719	SCO5719SC3C3.05c	hypothetical protein
**SCO5731**	0.70282232	0.02252611	1101170	SCO5731	SCO5731SC3C3.17c	serine protease
**SCO2962**	0.6833757	0.03620477	1098395	SCO2962	SCO2962SCE59.21c	bifunctional transferase/deacetylase membrane spanning
**SCO3791**	0.68302483	0.00967171	1099227	SCO3791	SCO3791SCH63.38	hypothetical protein
**SCO3148**	0.66795775	0.03892634	1098582	SCO3148	SCO3148SCE66.27c	4-diphosphocytidyl-2-C-methyl-D-erythritol kinase
**SCO5734**	0.65853344	0.03651915	1101173	SCO5734	SCO5734SC3C3.20c	ATP/GTP binding protein membrane protein
**SCO4423**	0.6542979	0.04577039	1099863	SCO4423	SCO4423SC6F11.21, SCD6.01, afsK	**Ser/Thr protein kinase**
**SCO2051**	0.64578243	0.04603788	1097485	hisH	SCO2051SC4G6.20c	imidazole glycerol phosphate synthase subunit HisH
**SCO2303**	0.63952223	0.01674217	1097737	SCO2303	SCO2303SCC30.11	hypothetical protein 55aa
**SCO3767**	0.63533862	0.01146803	1099203	SCO3767	SCO3767SCH63.14	hypothetical protein, TerB tellurium resistance
**SCO3381**	0.63398957	0.04223023	1098818	SCO3381	SCO3381SCE94.32c, nadC	nicotinate-nucleotide pyrophosphorylase
**SCO5835**	0.6213556	0.04275658	1101277	SCO5835	SCO5835SC9B10.02	ATP/GTP-binding protein
**SCO2455**	0.59068226	0.02904178	1097889	SCO2455	SCO2455SCC24.26c	spermidine synthase
**SCO4039**	0.57947617	0.04596553	1099475	SCO4039	SCO40392SCD60.05c	hypothetical protein, putative tRNA adenosine deaminase
**SCO3343**	0.57866166	0.04633327	1098780	SCO3343	SCO3343SCE7.10c	hypothetical protein
**SCO5252**	-0.5940067	0.04352194	1100693	SCO5252	SCO52522SC7G11.14	hypothetical protein
**SCO2067**	-0.6371408	0.02022089	1097501	SCO2067	SCO2067SC4G6.36	hypothetical protein
**SCO0805**	-0.6802069	0.02791649	1096228	SCO0805	SCO0805SCF43.16c	prolyl aminopeptidase
**SCO2004**	-0.6820355	0.01963905	1097438	SCO2004	SCO2004SC7H2.18	formate dehydrogenase
**SCO4872**	-0.6923778	0.04984318	1100313	SCO4872	SCO4872SCK20.13c	hypothetical protein
**SCO4822**	-0.7060296	0.02779588	1100263	SCO4822	SCO4822SC2A6.07c	hypothetical protein
**SCO1024**	-0.7096473	0.03665338	1096447	SCO1024	SCO1024SCG20A.04	hypothetical protein
**SCO4259**	-0.7398655	0.01735029	1099699	SCO4259	SCO4259SCD8A.32c	ATPase AAA
**SCO1314**	-0.7449593	0.048915	1096737	SCO1314	SCO1314SCBAC36F5.25c	sugar acetyltransferase
**SCO0101**	-0.7473248	0.0453803	1095526	SCO0101	SCO0101SCJ11.30c	hypothetical protein
**SCO3484**	-0.7481896	0.04314368	1098921	SCO3484	SCO3484SCE65.20c	sugar-binding protein
**SCO3356**	-0.7550618	0.0239024	1098793	SCO3356	SCO3356SCE94.07	**ECF sigma factor, SigE**
**SCO6623**	-0.7602652	0.0476237	1102062	SCO6623	SCO6623SC1F2.20	ATP/GTP binding protein

**SCO4964**	-0.7700103	0.01450179	1100405	SCO4964	SCO49642SCK31.24	integral membrane transport protein
**SCO3975**	-0.7733291	0.01371516	1099411	SCO3975	SCO3975SCBAC25E3.12c	**regulator**
**SCO6008**	-0.7901769	0.0374392	1101450	SCO6008	SCO6008SC7B7.05	transcriptional repressor protein
**SCO0087**	-0.7922533	0.01880639	1095515	SCO0087	SCO0087SCJ11.16c	hypothetical protein
**SCO2318**	-0.7996268	0.01396731	1097752	SCO2318	SCO2318SCC53.09c	glycosyl transferase
**SCO6065**	-0.8065341	0.04414436	1101506	SCO6065	SCO6065SC9B1.12	substrate-binding protein
**SCO5214**	-0.8084335	0.04385642	1100655	SCO5214	SCO5214SC7E4.11	hypothetical protein
**SCO6789**	-0.8148398	0.02012818	1102228	SCO6789	SCO6789SC6A5.38	fatty oxidation protein
**SCO1989**	-0.818492	0.049638	1097423	SCO1989	SCO1989SC7H2.03c	aminopeptidase
**SCO2954**	-0.8218257	0.04433948	1098387	SCO2954	SCO2954SCE59.13c	RNA polymerase sigma factor SigU; PMID: 18065550 elevates extracellular proteases
**SCO3203**	-0.8218867	0.02858457	1098637	SCO3203	SCO3203SCE22.20, bar	phosphinothricin acetyltransferase
**SCO1755**	-0.8225538	0.04504712	1097186	SCO1755	SCO17552SCI34.08c	hypothetical protein
**SCO4813**	-0.8258495	0.02229004	1100254	purN	SCO4813SCD63A.24	phosphoribosylglycinamide formyltransferase
**SCO6178**	-0.8348784	0.02091978	1101619	SCO6178	SCO6178SC6C5.14c	deacetylase, secreted possible PG deacetylase
**SCO1511**	-0.8364716	0.04011516	1096937	SCO1511	SCO1511SC9C5.35, SCL2.01	hypothetical protein
**SCO4994**	-0.8446655	0.03071142	1100435	SCO4994	SCO49942SCK36.17	hypothetical protein
**SCO7236**	-0.8456504	0.03761518	1102674	SCO7236	SCO7236qcrB3	ubiquinol-cytochrome C reductase cytochrome subunit B
**SCO1443**	-0.8515811	0.00244105	1096869	SCO1443	SCO1443SC6D7A.06c	riboflavin synthase subunit alpha
**SCO5385**	-0.8588378	0.02393705	1100825	SCO5385	SCO53852SC6G5.29	3-hydroxybutyryl-CoA dehydrogenase
**SCO4869**	-0.8623426	0.01576042	1100310	SCO4869	SCO4869SCK20.10, mutA2	methylmalonyl CoA mutase
**SCO4441**	-0.8719573	0.04498402	1099881	SCO4441	SCO4441SCD6.19	**DNA-binding protein**
**SCO4514**	-0.8785804	0.02885008	1099954	SCO4514	SCO4514SCD35.21c	hypothetical protein
**SCO6428**	-0.8805981	0.01562531	1101867	SCO6428	SCO6428SC1A6.17c	hypothetical protein
**SCO4777**	-0.8899836	0.01019354	1100218	SCO4777	SCO4777SCD63.09, pkaD	**protein Ser/Thr kinase**
**SCO5194**	-0.8992806	0.03228327	1100635	SCO5194	SCO51942SC3B6.18	hypothetical protein
**SCO1430**	-0.8995075	0.04334774	1096856	SCO1430	SCO1430SC6D7.09	**TetR family transcriptional regulator**
**SCO6053**	-0.9112592	0.02251604	1101494	SCO6053	SCO6053SC1B5.13c	hypothetical protein
**SCO0735**	-0.9120643	0.0406046	1096158	SCO0735	SCO07353SC5B7.13	oxidoreductase
**SCO5189**	-0.9121729	0.03121788	1100630	SCO5189	SCO51892SC3B6.13	hypothetical protein **CL35 Strakova et al**
**SCO4447**	-0.9167142	0.01917631	1099887	SCO4447	SCO4447SCD6.25	hypothetical protein
**SCO1444**	-0.9178788	0.02589119	1096870	SCO1444	SCO1444SCL6.01	chitinase
**SCO1982**	-0.9294227	0.01603753	1097416	SCO1982	SCO1982SC3C9.17c	hypothetical protein
**SCO7824**	-0.9412907	0.02948357	1103262	SCO7824	SCO7824SC8E7.21c	**TetR family transcriptional regulator**
**SCO5850**	-0.9423436	0.04083458	1101292	SCO5850	SCO5850SC9B10.17	hypothetical protein
**SCO5660**	-0.9458206	0.0383107	1101099	SCO5660	SCO5660SC6A9.07	AmpC beta-lactamase distantly related to pfam00905 and PF00768 D-alanyl-D-alanine carboxypeptidase.
**SCO2626**	-0.959086	0.03701158	1098060	SCO2626	SCO2626SCC80.11c	DNA repair hydrolase (fragment)
**SCO6004**	-0.9663489	0.01526711	1101446	SCO6004	SCO6004SC7B7.01c, SCBAC1C11.07c	ATP/GTP binding protein
**SCO0236**	-0.9692137	0.02582976	1095660	SCO0236	SCO0236SCJ9A.15c	DNA-binding protein
**SCO0105**	-0.9719724	0.03671155	1095530	SCO0105	SCO0105SCJ11.34c, xlnC	endo-1,4-beta-xylanase
**SCO4258**	-0.9746007	0.04326187	1099698	SCO4258	SCO4258SCD8A.31	hydrolytic protein carbohydrate binding
**SCO4598**	-0.9760018	0.04345377	1100038	SCO4598	SCO4598SCD20.16c	**two-component system sensor kinase**
**SCO5043**	-0.9948104	0.01407193	1100484	SCO5043	SCO5043SCK7.16c	hydrolase membrane protein
**SCO7823**	-1.0032944	0.04731835	1103261	SCO7823	SCO7823SC8E7.20c	hypothetical protein
**SCO2383**	-1.0044901	0.04910985	1097817	SCO2383	SCO2383SC4A7.11	hypothetical protein
**SCO5045**	-1.0056495	0.01096671	1100486	SCO5045	SCO5045SCK7.18	hypothetical protein
**SCO5044**	-1.0075861	0.02148664	1100485	SCO5044	SCO5044SCK7.17c, fumB	fumarate hydratase class I
**SCO0535**	-1.0143617	0.01769636	1095958	SCO0535	SCO0535SCF11.15	hydrolase MhpC like
**SCO2953**	-1.0144104	0.04179123	1098386	SCO2953	SCO2953SCE59.12c	hypothetical protein

**SCO7095**	-1.0150053	0.03613319	1102533	SCO7095	SCO7095SC3A4.21c	hydrolase
**SCO6388**	-1.0174551	0.01903839	1101829	SCO6388	SCO6388SC3C8.07c	hypothetical protein
**SCO1887**	-1.0177796	0.03493298	1097321	SCO1887	SCO1887SCI7.05c	integral membrane transport protein
**SCO1895**	-1.0187911	0.03939762	1097329	SCO1895	SCO1895SCI7.13c	5-dehydro-4-deoxyglucarate dehydratase
**SCO4079**	-1.0247206	0.00102117	1099516	SCO4079	SCO4079SCD25.15, purL	phosphoribosylformylglycinamidine synthase II
**SCO3009**	-1.0258731	0.01741134	1098442	SCO3009	SCO3009SCE33.11c	hypothetical protein
**SCO5783**	-1.0278388	0.00975505	1101225	SCO5783	SCO5783SC4H2.04c	hypothetical protein
**SCO5399**	-1.0292836	0.02462097	1100839	SCO5399	SCO5399SC8F4.03	acetyl-CoA acetyltransferase
**SCO2561**	-1.0348763	0.00039975	1097995	SCO2561	SCO2561SCC77.28c	long-chain fatty-acid CoA ligase
**SCO5028**	-1.0410003	0.0041875	1100469	SCO5028	SCO5028SCK7.01	ATP-binding protein
**SCO4759**	-1.0503524	0.0200821	1100200	SCO4759	SCO4759SC6G4.37	hypothetical protein
**SCO4055**	-1.0507445	0.01739508	1099491	SCO4055	SCO40552SCD60.21c	alcohol dehydrogenase
**SCO4078**	-1.0512646	0.00106214	1099515	SCO4078	SCO4078SCD25.14, purQ	phosphoribosylformylglycinamidine synthase I
**SCO2469**	-1.0514186	0.0171081	1097903	SCO2469	SCO2469SC7A8.08c	reductase
**SCO6732**	-1.052917	0.00200693	1102171	SCO6732	SCO6732SC5F2A.15	fatty acid oxidative multifunctional enzyme
**SCO1396**	-1.0549897	0.02383087	1096822	SCO1396	SCO1396SC1A8A.16c	D-alanyl-D-alanine dipeptidase
**SCO0346**	-1.0560937	0.03628197	1095769	SCO0346	SCO0346SCF41.05	2-hydroxyhepta-2,4-diene-1,7-dioate isomerase
**SCO1528**	-1.0595903	0.03660664	1096954	SCO1528	SCO1528fusA	elongation factor G
**SCO4352**	-1.064125	0.01892397	1099792	SCO4352	SCO4352SCD19.07	oxidoreductase
**SCO1224**	-1.0648446	0.04231456	1096647	SCO1224	SCO12242SCG58.24c	sugar-phosphate isomerase
**SCO7611**	-1.0702186	0.03149459	1103049	SCO7611	SCO7611SC2H2.09	hypothetical protein
**SCO0079**	-1.0703477	0.02171802	1095508	SCO0079	SCO0079SCJ11.08c	integral membrane transport protein
**SCO0766**	-1.073018	0.02951067	1096189	SCO0766	SCO0766SCF81.25c	beta-galactosidase
**SCO1533**	-1.0864658	0.02593568	1096959	SCO1533	SCO1533SCL2.23c	hypothetical protein
**SCO7012**	-1.0947742	0.03087336	1102450	SCO7012	SCO7012SC1H10.01c, SC8F11.38c	binding protein dependent transport protein
**SCO6952**	-1.100734	0.02483597	1102390	SCO6952	SCO6952SC6F7.05c	hypothetical protein
**SCO2018**	-1.1019587	0.03172033	1097452	SCO2018	SCO2018SC7H2.32c	aminopeptidase pepN
**SCO5026**	-1.1025966	0.0339411	1100467	SCO5026	SCO5026SCK15.28	hypothetical protein
**SCO3563**	-1.1038638	0.02627836	1098999	SCO3563	SCO3563SCH5.26, acsA	acetyl-CoA synthetase
**SCO0439**	-1.1074638	0.01211983	1095862	SCO0439	SCO0439SCF51A.17c	hypothetical protein
**SCO5657**	-1.1108087	0.03763986	1101096	SCO5657	SCO5657SC6A9.10c	aldehyde dehydrogenase
**SCO2800**	-1.1120818	0.03056538	1098234	SCO2800	SCO28002SCC13.08c	two component system histidine kinase
**SCO4077**	-1.1151632	0.00385661	1099514	SCO4077	SCO4077SCD25.13	phosphoribosylformylglycinamidine synthase subunit PurS
**SCO3164**	-1.1225888	0.01811912	1098598	SCO3164	SCO3164SCE87.15c	hypothetical protein
**SCO5025**	-1.1237068	0.01203305	1100466	SCO5025	SCO5025SCK15.27	**transcriptional regulator**
**SCO4005**	-1.1246788	0.03083396	1099441	SCO4005	SCO40052SC10A7.09	**RNA polymerase sigma factor; PMID: 17683547 Induced by ppGpp**
**SCO6814**	-1.1248084	0.0492456	1102253	SCO6814	SCO6814SC1A2.23c	ABC transporter ATP-binding protein
**SCO5299**	-1.1254025	0.0497461	1100739	SCO5299	SCO5299SC6G9.34	hypothetical protein
**SCO7710**	-1.1356304	0.03537994	1103148	SCO7710	SCO7710SC8D11.01, SCBAC12C8.11	phosphotransferase
**SCO6384**	-1.1416249	0.00261905	1101825	SCO6384	SCO6384SC3C8.03c	integral membrane lysyl-tRNA synthetase
**SCO0783**	-1.1431431	0.01181955	1096206	SCO0783	SCO07833SCF60.15, tetM	tetracycline resistance protein
**SCO6399**	-1.1546158	0.01041136	1101838	SCO6399	SCO6399SC3C8.18c	hypothetical protein
**SCO5398**	-1.1549266	0.03979902	1100838	SCO5398	SCO5398SC8F4.02c	hypothetical protein, putative methylmalonyl-CoA epimerase.
**SCO7013**	-1.1577663	0.02666938	1102451	SCO7013	SCO7013SC1H10.02c	sugar-binding lipoprotein
**SCO4071**	-1.1583569	0.0117192	1099508	hemH	SCO4071SCD25.07, purC	phosphoribosylaminoimidazole-succinocarboxamide synthase
**SCO4440**	-1.1593685	0.04985673	1099880	SCO4440	SCO4440SCD6.18	hypothetical protein
**SCO6027**	-1.1600757	0.04632064	1101468	SCO6027	SCO6027SC1C3.15c	acetyl-CoA acetyltransferase
**SCO5035**	-1.1642095	0.04903448	1100476	SCO5035	SCO5035SCK7.08c	ABC transporter ATP-binding protein

**SCO1440**	-1.1668322	0.00635516	1096866	ribH	SCO1440SC6D7A.03c	6,7-dimethyl-8-ribityllumazine synthase
**SCO6373**	-1.1678056	0.04497161	1101814	SCO6373	SCO6373SC4A2.09	hypothetical protein
**SCO7555**	-1.1678075	0.0498048	1102993	SCO7555	SCO7555SC5F1.09	solute binding lipoprotein
**SCO1441**	-1.1684141	0.00281166	1096867	SCO1441	SCO1441SC6D7A.04c, ribAB	bifunctional 3,4-dihydoxy-2-butanone 4-phosphate synthase/GTP cyclohydrolase II protein
**SCO5148**	-1.1710116	0.04570437	1100589	SCO5148	SCO5148SCP8.11	hypothetical protein
**SCO1240**	-1.188578	0.04799388	1096663	SCO1240	SCO12402SCG1.15	hypothetical protein **CL35 Strakova et al**
**SCO5782**	-1.1938642	0.02696515	1101224	SCO5782	SCO5782SC4H2.03c	transmembrane transport protein
**SCO0021**	-1.1993581	0.00441403	1095456	SCO0021	SCO0021SCJ4.02	hypothetical protein
**SCO5258**	-1.2014174	0.04519989	1100699	SCO5258	SCO52582SC7G11.20c, atrC	ATP-binding protein
**SCO0322**	-1.2027997	0.01226895	1095746	SCO0322	SCO0322SC5G9.31c, SCF12.01c	ABC transport ATP-binding subunit
**SCO7544**	-1.2072474	0.0431692	1102982	SCO7544	SCO7544SC8G12.20	ABC transporter membrane protein
**SCO0309**	-1.212534	0.03364248	1095733	SCO0309	SCO0309SC5G9.18c	amide hydrolase
**SCO0600**	-1.2169915	0.01965013	1096023	SCO0600	SCO0600SCF55.24, sig8	**RNA polymerase sigma factor sig8/sigB**
**SCO2729**	-1.2195013	0.02878267	1098163	SCO2729	SCO2729SCC46.14	acetyltransferase
**SCO2698**	-1.221986	0.02201606	1098132	SCO2698	SCO2698SCC61A.19	small hydrophilic protein
**SCO6730**	-1.2250856	0.02620335	1102169	SCO6730	SCO6730SC5F2A.13	racemase
**SCO4564**	-1.2284437	0.02100994	1100004	SCO4564	SCO4564SCD16A.19c, nuoC	NADH dehydrogenase subunit C
**SCO7410**	-1.2303826	0.01798887	1102848	SCO7410	SCO7410SC6D11.06c	binding-protein dependent transport protein
**SCO7197**	-1.2305069	0.01913662	1102635	SCO7197	SCO7197SC8A11.25c	amino acid ABC transporter permease
**SCO7265**	-1.2319619	0.02199026	1102703	SCO7265	SCO7265SC5H1.27	hypothetical protein
**SCO6332**	-1.2339692	0.04536992	1101773	SCO6332	SCO6332SC10H5.08c	hypothetical protein
**SCO0137**	-1.2374128	0.01701312	1095561	SCO0137	SCO0137SCJ21.18c, SCJ33.01c	sugar-transport protein
**SCO3010**	-1.2375496	0.01237786	1098443	SCO3010	SCO3010SCE33.12c	hypothetical protein
**SCO1218**	-1.2390603	0.03868886	1096641	SCO1218	SCO12182SCG58.18c	transmembrane transport protein
**SCO0815**	-1.2408014	0.03540589	1096238	SCO0815	SCO0815SCF43A.05	hypothetical protein
**SCO4256**	-1.2517025	0.00725892	1099696	SCO4256	SCO4256SCD8A.29	hydrolytic protein carbohydrate binding
**SCO1366**	-1.2517451	0.04440305	1096789	SCO1366	SCO1366SC10A9.08c	hypothetical protein
**SCO5259**	-1.2577006	0.0321283	1100700	SCO5259	SCO52592SC7G11.21c, atrB	permease
**SCO5017**	-1.2578031	0.04475442	1100458	SCO5017	SCO5017SCK15.19	**AraC family transcription regulator**
**SCO1729**	-1.261507	0.04290643	1097160	SCO1729	SCO1729SCI11.18	hypothetical protein
**SCO1988**	-1.2627759	0.00476581	1097422	SCO1988	SCO1988SC7H2.02	hypothetical protein
**SCO4783**	-1.2705064	0.03836347	1100224	SCO4783	SCO4783SCD63.15	hypothetical protein
**SCO1147**	-1.2715908	0.00116743	1096570	SCO1147	SCO11472SCG38.40, SCG8A.01	ABC transporter transmembrane subunit **CL35 Strakova et al**
**SCO6731**	-1.2756499	0.00054097	1102170	SCO6731	SCO6731SC5F2A.14	acetyl-CoA acetyltransferase
**SCO3815**	-1.2959344	0.02615942	1099251	SCO3815	SCO3815SCGD3.16c, bkdC1	branched-chain alpha-keto acid dehydrogenase E2
**SCO5415**	-1.2985974	0.02764478	1100855	SCO5415	SCO5415SC8F4.19, icmA	isobutyryl-CoA mutase A
**SCO2827**	-1.2993047	0.03393689	1098261	SCO2827	SCO2827SCBAC17F8.18c	hypothetical protein
**SCO6719**	-1.3019551	0.02322505	1102158	SCO6719	SCO6719SC5F2A.02c	UvrA-like ABC transporter
**SCO4257**	-1.3041952	0.00119154	1099697	SCO4257	SCO4257SCD8A.30	hydrolytic protein carbohydrate binding
**SCO1442**	-1.3149347	0.00215866	1096868	SCO1442	SCO1442SC6D7A.05c	hypothetical protein
**SCO3667**	-1.319192	0.03204494	1099103	SCO3667	SCO3667SCH44.07c	solute-binding protein
**SCO0409**	-1.3258528	0.04350296	1095832	SCO0409	SCO0409SCF51.08c, sapA	spore-associated protein
**SCO5785**	-1.325935	0.00822481	1101227	SCO5785	SCO5785SC4H2.06	**two-component regulator**
**SCO0725**	-1.331583	0.00628857	1096148	SCO0725	SCO07253SC5B7.03	hypothetical protein
**SCO5380**	-1.3330979	0.03992505	1100820	SCO5380	SCO53802SC6G5.24	hypothetical protein
**SCO0939**	-1.3341378	0.0480469	1096362	SCO0939	SCO0939SCM10.27c	hydrolase MhpC like
**SCO0533**	-1.3351314	0.04425517	1095956	SCO0533	SCO0533SCF11.13	sugar transporter membrane protein
**SCO3816**	-1.3426793	0.03257553	1099252	SCO3816	SCO3816SCGD3.17c, bkdB1	branched-chain alpha-keto acid dehydrogenase E1 subunit beta **CL35 Strakova et** al

**SCO1640**	-1.3439945	0.04030802	1097071	SCO1640	SCO1640SCI41.23c	hypothetical protein
**SCO2587**	-1.3453967	2.04E-05	1098021	SCO2587	SCO2587proB	gamma-glutamyl kinase
**SCO1082**	-1.3478495	4.91E-05	1096505	SCO1082	SCO1082SCG22.28c	electron transfer flavoprotein subunit beta
**SCO5973**	-1.3483439	0.02510209	1101415	SCO5973	SCO5973StBAC16H6.08	phosphatase
**SCO5429**	-1.3487441	0.02978033	1100869	SCO5429	SCO5429SC6A11.05c	integral membrane transport protein
**SCO7277**	-1.3674692	0.00197996	1102715	SCO7277	SCO7277SC5H1.15c	**regulator protein**
**SCO1354**	-1.3681399	0.03479062	1096777	SCO1354	SCO13542SCG61.36c	hypothetical protein
**SCO0260**	-1.3738624	0.02544004	1095684	SCO0260	SCO0260SCF1.02	hypothetical protein
**SCO6784**	-1.3774459	0.0109934	1102223	SCO6784	SCO6784SC6A5.33c	regulatory protein
**SCO2673**	-1.3846957	0.03016795	1098107	SCO2673	SCO2673SC6D10.16	hypothetical protein
**SCO6721**	-1.3855864	0.00090274	1102160	SCO6721	SCO6721SC5F2A.04	hypothetical protein
**SCO1732**	-1.3877999	0.01331755	1097163	SCO1732	SCO1732SCI11.21	hypothetical protein
**SCO6228**	-1.3887744	0.02487651	1101669	SCO6228	SCO6228SC2H4.10	hypothetical protein
**SCO7028**	-1.3955846	0.01850385	1102466	SCO7028	SCO7028SC1H10.17	sugar-binding lipoprotein
**SCO4262**	-1.4022549	0.00101382	1099702	SCO4262	SCO4262SCD49.03	hypothetical protein
**SCO0203**	-1.4034202	0.01489267	1095627	SCO0203	SCO0203SCJ12.15c	two-component sensor
**SCO5884**	-1.4050464	0.03746746	1101326	SCO5884	SCO5884SC3F7.04c	hypothetical protein
**SCO1669**	-1.4066087	0.0027955	1097100	SCO1669	SCO1669SCI52.11c	ATP/GTP-binding protein
**SCO3113**	-1.4072659	0.03319066	1098547	SCO3113	SCO3113SCE41.22	SCO3113 transposase remnant
**SCO6738**	-1.4109534	0.01799603	1102177	SCO6738	SCO6738SC5F2A.21	carboxypeptidase
**SCO4597**	-1.4173771	0.02562279	1100037	SCO4597	SCO4597SCD20.15c	**two-component system sensor kinase**
**SCO0952**	-1.4236577	0.00326967	1096375	SCO0952	SCO0952SCM11.07c	solute-binding protein
**SCO1914**	-1.4269417	0.02597813	1097348	SCO1914	SCO1914SCI7.32	hypothetical protein
**SCO6011**	-1.431962	4.81E-05	1101453	SCO6011	SCO6011SC7B7.08	ABC transporter
**SCO0974**	-1.4402864	0.03534966	1096397	SCO0974	SCO0974SCBAC19F3.01c, SCM11.29c	hypothetical protein
**SCO4681**	-1.4438688	0.00668191	1100122	SCO4681	SCO4681SCD31.06	short chain dehydrogenase
**SCO3957**	-1.4453186	0.04694573	1099393	SCO3957	SCO3957SCD78.24	hypothetical protein **CL35 Strakova et al**
**SCO2493**	-1.4633095	0.00241752	1097927	SCO2493	SCO2493SC7A8.32c	hypothetical protein
**SCO1839**	-1.4660987	0.04727474	1097273	SCO1839	SCO1839SCI8.24c	**transcriptional regulator**
**SCO5260**	-1.4737187	0.03445479	1100701	SCO5260	SCO52602SC7G11.22c, atrA	hypothetical protein
**SCO5229**	-1.4737989	0.02312831	1100670	SCO5229	SCO5229SC7E4.26c	permease
**SCO3958**	-1.4739248	0.03536152	1099394	SCO3958	SCO3958SCD78.25	ABC transporter ATP-binding protein
**SCO6010**	-1.4793502	3.57E-05	1101452	SCO6010	SCO6010SC7B7.07	ABC transporter ATP-binding protein
**SCO6989**	-1.4920113	0.0316697	1102427	SCO6989	SCO6989SC8F11.15c	hypothetical protein
**SCO1056**	-1.4933751	0.02832881	1096479	SCO1056	SCO1056SCG22.02	sugar transport sugar binding protein
**SCO0591**	-1.5009327	0.02864391	1096014	SCO0591	SCO0591SCF55.15	endo-N-acetylmuramidases
**SCO1340**	-1.5083141	0.00412099	1096763	SCO1340	SCO13402SCG61.22	hypothetical protein
**SCO6718**	-1.5121362	0.03719433	1102157	SCO6718	SCO6718SC5F2A.01c, ddah	dimethylarginine dimethylaminohydrolase
**SCO2925**	-1.5136013	0.01115333	1098358	SCO2925	SCO2925SCE19A.25c	oxidoreductase subunit
**SCO6968**	-1.5190717	0.01563016	1102406	SCO6968	SCO6968SC6F7.21	long-chain-fatty-acid-CoA ligase
**SCO0178**	-1.5248701	0.0288573	1095602	SCO0178	SCO0178SCJ1.27	hypothetical protein
**SCO0255**	-1.5264789	0.00996091	1095679	SCO0255	SCO0255SCF20.01c, SCJ9A.34c	**sigma factor**
**SCO5230**	-1.5281394	0.015086	1100671	SCO5230	SCO5230SC7E4.27c	hypothetical protein
**SCO4254**	-1.5326734	0.01373842	1099694	SCO4254	SCO4254SCD8A.27	hypothetical protein
**SCO0544**	-1.5402096	0.02949671	1095967	SCO0544	SCO0544SCF11.24	hypothetical protein
**SCO5889**	-1.5469515	0.04596249	1101331	SCO5889	SCO5889SC3F7.09, redO	hypothetical protein
**SCO2999**	-1.5472914	0.00442127	1098432	SCO2999	SCO2999SCE33.01c, SCE99.06c	Bacterial NAD-glutamate dehydrogenase.
**SCO1112**	-1.5553157	0.00840536	1096535	SCO1112	SCO11122SCG38.05	oxidoreductase

**SCO0698**	-1.5567765	0.00788208	1096121	SCO0698	SCO0698SCF42.08c	hypothetical protein
**SCO2895**	-1.5659291	0.03439587	1098328	SCO2895	SCO2895SCE6.32c	integral membrane transport protein
**SCO5324**	-1.5674382	0.00252939	1100764	SCO5324	SCO5324SC6G9.09c	oxidoreductase
**SCO2247**	-1.5709454	0.03633818	1097680	SCO2247	SCO2247SC1G2.09c	hypothetical protein
**SCO1081**	-1.5725544	9.34E-05	1096504	SCO1081	SCO1081SCG22.27c	electron transfer flavoprotein subunit alpha
**SCO0540**	-1.5736178	0.02723296	1095963	SCO0540	SCO0540SCF11.20	sugar transport membrane protein
**SCO0876**	-1.5859918	0.04100203	1096299	SCO0876	SCO0876SCM1.09c	hypothetical protein
**SCO6702**	-1.6056288	0.00962701	1102141	SCO6702	SCO6702SC4C6.12c, pcaJ	3-oxoadipate CoA-transferase subunit B
**SCO1585**	-1.6090088	0.01702944	1097016	SCO1585	SCO1585SCI35.07c	hypothetical protein
**SCO6009**	-1.6099713	1.18E-05	1101451	SCO6009	SCO6009SC7B7.06	solute-binding protein, possible xylose binding
**SCO0009**	-1.6102124	0.02088183	1095441	SCO0009	SCO0009SCJ30.04c	hypothetical protein
**SCO2746**	-1.6125097	0.03043273	1098180	SCO2746	SCO2746SCC57A.17	ABC transporter ATP-binding protein
**SCO4222**	-1.6198737	0.00140301	1099662	SCO4222	SCO42222SCD46.36	hypothetical protein
**SCO7221**	-1.6234576	0.04543529	1102659	SCO7221	SCO7221SC2H12.20c	polyketide synthase
**SCO2829**	-1.6272084	0.01469204	1098263	SCO2829	SCO2829SCE20.03	amino acid ABC transporter transmembrane protein
**SCO4828**	-1.6282017	0.03599873	1100269	SCO4828	SCO4828SC2A6.13, gbsA	betaine aldehyde dehydrogenase
**SCO4255**	-1.6372744	0.00033718	1099695	SCO4255	SCO4255SCD8A.28	hypothetical protein **CL35 Strakova et a**l
**SCO0427**	-1.6384131	0.00220241	1095850	SCO0427	SCO0427SCF51A.05	hydrolase MhpC like
**SCO5656**	-1.6386654	0.01930151	1101095	SCO5656	SCO5656SC6A9.11	**transcriptional regulator**
**SCO0410**	-1.640241	0.00927831	1095833	SCO0410	SCO0410SCF51.09c	hypothetical protein
**SCO2379**	-1.6424981	0.01374332	1097813	SCO2379	SCO2379SC4A7.07	acetyltransferase
**SCO6744**	-1.64427	0.00580727	1102183	SCO6744	SCO6744SC5F2A.27	hypothetical protein
**SCO1196**	-1.6458162	0.00851179	1096619	SCO1196	SCO1196SCG11A.27c	hypothetical protein
**SCO6500**	-1.647057	0.01839677	1101939	SCO6500	SCO6500gvpA	gas vesicle synthesis-like protein
**SCO2831**	-1.6531334	0.00634472	1098265	SCO2831	SCO2831SCE20.05	amino acid ABC transporter ATP-binding protein
**SCO5655**	-1.6614101	0.00694098	1101094	SCO5655	SCO5655SC6A9.12	hypothetical protein
**SCO7175**	-1.6638867	0.03859212	1102613	SCO7175	SCO7175SC8A11.03	regulator **CL35 Strakova et al**
**SCO1346**	-1.6683049	1.70E-06	1096769	SCO1346	SCO13462SCG61.28c, fabG3	3-oxoacyl-ACP reductase
**SCO2830**	-1.6766753	0.00459166	1098264	SCO2830	SCO2830SCE20.04	amino acid ABC transporter transmembrane protein
**SCO7806**	-1.6768261	0.00201268	1103244	SCO7806	SCO7806SC8E7.03c	**DNA-binding protein**
**SCO0592**	-1.688755	0.04393063	1096015	SCO0592	SCO0592SCF55.16c	hypothetical protein
**SCO7063**	-1.6892506	0.01408007	1102501	SCO7063	SCO7063SC4G1.29c	hypothetical protein
**SCO6745**	-1.6952238	0.0009285	1102184	SCO6745	SCO6745SC5F2A.28c	hypothetical protein
**SCO4006**	-1.7141558	0.00198959	1099442	SCO4006	SCO40062SC10A7.10	long-chain-fatty-acid--CoA ligase
**SCO0616**	-1.7174517	0.01027731	1096039	SCO0616	SCO0616SCF55.40c	hypothetical protein
**SCO6544**	-1.7174754	0.04932425	1101983	SCO6544	SCO6544SC5C7.29	hypothetical protein
**SCO7329**	-1.7188796	0.04711807	1102767	SCO7329	SCO7329SC4G10.08c	long-chain-fatty-acid-CoA ligase
**SCO6499**	-1.7201011	0.00108622	1101938	SCO6499	SCO6499gvpO	gas vesicle synthesis protein
**SCO2828**	-1.7208402	0.00298018	1098262	SCO2828	SCO2828SCE20.02	amino acid ABC transporter substrate-binding protein
**SCO3928**	-1.7218374	0.03277359	1099364	SCO3928	SCO3928SCQ11.11, thiC	thiamine biosynthesis protein ThiC
**SCO6987**	-1.7297005	0.02949153	1102425	SCO6987	SCO6987SC8F11.13	hypothetical protein
**SCO6433**	-1.7558408	0.00347704	1101872	SCO6433	SCO6433SC1A6.22	hypothetical protein
**SCO0111**	-1.7574439	0.02698317	1095536	SCO0111	SCO0111SCJ11.40	oxidoreductase
**SCO3710**	-1.7677601	0.01811382	1099146	SCO3710	SCO3710SCH35.14c	large integral membrane protein
**SCO6504**	-1.7729228	0.00985469	1101943	SCO6504	SCO6504SC1E6.13	hypothetical protein
**SCO1824**	-1.7815857	0.00935306	1097258	SCO1824	SCO1824ssp	subtilisin-like protease
**SCO7606**	-1.7816208	0.01054566	1103044	SCO7606	SCO7606SC2H2.04	amino acid binding protein
**SCO7608**	-1.7922566	0.02636609	1103046	SCO7608	SCO7608SC2H2.06	hypothetical protein

**SCO2007**	-1.8016251	0.0163183	1097441	SCO2007	SCO2007SC7H2.21	hypothetical protein
**SCO3079**	-1.8074593	4.25E-06	1098512	SCO3079	SCO3079SCE25.20	acetyl-CoA acetyltransferase
**SCO1683**	-1.8139783	0.00106767	1097114	SCO1683	SCO1683SCI30A.04c	amino acid permease
**SCO1317**	-1.8149255	0.00252191	1096740	SCO1317	SCO1317SCBAC36F5.28c	hypothetical protein
**SCO1453**	-1.8278918	0.00020913	1096879	SCO1453	SCO1453SCL6.10	hypothetical protein
**SCO1225**	-1.8353608	0.00033228	1096648	SCO1225	SCO12252SCG58.25	osmoprotectant transporter
**SCO6765**	-1.8372297	0.01354668	1102204	SCO6765	SCO6765SC6A5.14	lipoprotein
**SCO3247**	-1.8432387	0.03439402	1098681	SCO3247	SCO3247SCE29.16c	acyl CoA oxidase
**SCO7747**	-1.8437846	0.00200927	1103185	SCO7747	SCO7747SC8D11.38	hypothetical protein
**SCO2978**	-1.8456453	0.01011318	1098411	SCO2978	SCO2978SCE50.06	hypothetical protein
**SCO0543**	-1.8492501	8.91E-05	1095966	SCO0543	SCO0543SCF11.23	Murein DD-endopeptidase MepM and murein hydrolase activator NlpD, contain LysM domain
**SCO7311**	-1.8507136	0.03265893	1102749	SCO7311	SCO7311SC5F8.21c	amino acid decarboxylase
**SCO0408**	-1.8541555	0.0035851	1095831	SCO0408	SCO0408SCF51.07	methyltransferase
**SCO3711**	-1.855714	0.00155208	1099147	SCO3711	SCO3711SCH35.13c	small membrane protein
**SCO0273**	-1.8621277	0.01916741	1095697	SCO0273	SCO0273SCF1.15, SCF85.01	substrate binding protein
**SCO6503**	-1.8629766	0.00416809	1101942	SCO6503	SCO6503SC1E6.12	hypothetical protein
**SCO1838**	-1.8653484	0.00722646	1097272	SCO1838	SCO1838SCI8.23c	enoyl-CoA hydratase/isomerase
**SCO0435**	-1.8656458	0.01537345	1095858	SCO0435	SCO0435malY	aminotransferase
**SCO0618**	-1.866493	0.01869663	1096041	SCO0618	SCO0618SCF56.02	hypothetical protein
**SCO3055**	-1.8724747	0.001664	1098488	SCO3055	SCO3055SCBAC19G2.10	hypothetical protein **CL35 Strakova et al**
**SCO2248**	-1.8763558	0.02242751	1097681	SCO2248	SCO2248SC1G2.10	hypothetical protein
**SCO3051**	-1.8826185	6.97E-09	1098484	SCO3051	SCO3051SCBAC19G2.06c, fadE	acyl-CoA dehydrogenase
**SCO2980**	-1.8906462	0.02505335	1098413	SCO2980	SCO2980SCE50.08	integral membrane transport protein
**SCO1345**	-1.8925424	7.29E-06	1096768	fabG	SCO13452SCG61.27c, fabG2	3-ketoacyl-ACP reductase
**SCO0458**	-1.9212826	0.04442668	1095881	SCO0458	SCO0458SCF51A.36	Beta-glucosidase
**SCO7210**	-1.9217412	0.00194741	1102648	SCO7210	SCO7210SC2H12.09	hypothetical protein
**SCO2727**	-1.9218336	0.04248327	1098161	SCO2727	SCO2727SCC46.12c	hypothetical protein
**SCO1563**	-1.9265657	0.00454179	1096994	SCO1563	SCO1563SCL11.19c	acetyltransferase
**SCO3090**	-1.9277931	0.02030102	1098524	SCO3090	SCO3090SCE25.31	ABC transporter
**SCO3362**	-1.9347792	0.03096271	1098799	SCO3362	SCO3362SCE94.13	hypothetical protein
**SCO7596**	-1.9419372	0.00503083	1103034	SCO7596	SCO7596SC7H9.08c	integral membrane transport protein
**SCO5911**	-1.9424452	0.01533385	1101353	SCO5911	SCO5911SC10A5.16c	oligopeptide binding protein
**SCO2912**	-1.9444554	0.02690586	1098345	SCO2912	SCO2912SCE19A.12c	hypothetical protein
**SCO1307**	-1.9490462	0.00288363	1096730	SCO1307	SCO1307SCBAC36F5.18c	hypothetical protein
**SCO7721**	-1.9559289	0.0447063	1103159	SCO7721	SCO7721SC8D11.12	hypothetical protein
**SCO6362**	-1.9743461	0.00515902	1101803	SCO6362	SCO6362SC3A7.30	two-component sensor
**SCO2861**	-1.9801648	0.00104837	1098295	SCO2861	SCO2861SCE20.35.	hypothetical protein
**SCO1692**	-1.9919414	0.0140943	1097123	SCO1692	SCO1692SCI30A.13c	oxidoreductase
**SCO0992**	-1.9933745	0.02638156	1096415	SCO0992	SCO09922SCG2.05	cysteine synthase
**SCO5430**	-1.9986602	0.00190696	1100870	SCO5430	SCO5430SC6A11.06c	extracellular solute-binding lipoprotein
**SCO2417**	-2.0057761	0.00808366	1097851	SCO2417	SCO2417SC8A2.05c mce	hypothetical protein, possibly part of Mce protein complex
**SCO4980**	-2.013444	1.19E-05	1100421	SCO4980	SCO49802SCK36.03c	hypothetical protein
**SCO4930**	-2.0147131	0.01007507	1100371	SCO4930	SCO4930SCK13.22	enoyl-CoA hydratase
**SCO0596**	-2.0161697	0.00065766	1096019	SCO0596	SCO0596SCF55.20	DNA-binding protein
**SCO5915**	-2.0169579	0.02003694	1101357	SCO5915	SCO5915SC10A5.20	hypothetical protein
**SCO3607**	-2.0193936	0.0002716	1099043	SCO3607	SCO3607SC66T3.18c	hypothetical protein
**SCO4207**	-2.0205335	0.00545021	1099647	SCO4207	SCO42072SCD46.21	hypothetical protein **CL35 Strakova et al**
**SCO6700**	-2.024708	0.0228484	1102139	SCO6700	SCO6700SC4C6.10c, pcaH	protocatechuate 3,4-dioxygenase subunit beta

**SCO2401**	-2.0352812	0.00081296	1097835	SCO2401	SCO2401SC4A7.29	dehydratase
**SCO4562**	-2.0574858	0.00263565	1100002	SCO4562	SCO4562SCD16A.21c, nuoA	NADH dehydrogenase subunit A
**SCO0257**	-2.0580139	0.018112	1095681	SCO0257	SCO0257SCF20.03	hypothetical protein
**SCO2749**	-2.063794	0.00078669	1098183	SCO2749	SCO2749SCC57A.20	D-ribose pyranase
**SCO2774**	-2.0670871	2.15E-09	1098208	SCO2774	SCO2774SCC105.05c, acdH2	acyl-CoA dehydrogenase
**SCO2466**	-2.0813891	0.00766622	1097900	SCO2466	SCO2466SC7A8.05	hypothetical protein
**SCO6986**	-2.0879227	0.04448497	1102424	SCO6986	SCO6986SC8F11.12	**DNA-binding protein**
**SCO5916**	-2.0968425	0.01802199	1101358	SCO5916	SCO5916SC10A5.21	hypothetical protein
**SCO2213**	-2.099307	0.0191776	1097646	SCO2213	SCO2213SC10B7.08	**regulatory protein**
**SCO3945**	-2.1005832	0.00285678	1099381	SCO3945	SCO3945SCD78.12, cydA	cytochrome oxidase subunit I
**SCO7040**	-2.1078393	0.00076517	1102478	SCO7040	SCO7040SC4G1.06c, gap2	glyceraldehyde-3-phosphate dehydrogenase
**SCO2625**	-2.1165534	0.00384573	1098059	SCO2625	SCO2625SCC80.10	hypothetical protein
**SCO4477**	-2.1400828	0.00961205	1099917	SCO4477	SCO4477SCD65.20c	**MerR family transcriptional regulator**
**SCO1230**	-2.1437321	0.01162163	1096653	SCO1230	SCO12302SCG1.05c	tripeptidylaminopeptidase
**SCO6506**	-2.1450096	0.00787908	1101945	SCO6506	SCO6506gvpL	gas vesicle protein
**SCO6516**	-2.1493833	0.00124771	1101955	SCO6516	SCO6516SC1E6.25c, SC5C7.01c	hypothetical protein
**SCO1457**	-2.1642324	0.00052255	1096883	SCO1457	SCO1457SCL6.14c	transporter
**SCO6475**	-2.1729374	0.01552382	1101914	SCO6475	SCO6475SC9C7.11c	oxidoreductase
**SCO0107**	-2.1747363	5.06E-06	1095532	SCO0107	SCO0107SCJ11.36c	aminoglycoside nucleotidyltransferase
**SCO4054**	-2.1774137	0.00129533	1099490	SCO4054	SCO40542SCD60.20	hypothetical protein
**SCO4563**	-2.197312	0.00288274	1100003	SCO4563	SCO4563SCD16A.20c, nuoB	NADH dehydrogenase subunit B
**SCO6511**	-2.2095224	0.00464603	1101950	SCO6511	SCO6511SC1E6.20c	hypothetical protein
**SCO2009**	-2.2236138	0.0037835	1097443	SCO2009	SCO2009SC7H2.23	branched-chain amino acid ABC transporter permease
**SCO3946**	-2.2292424	0.00454854	1099382	SCO3946	SCO3946SCD78.13, cydB	cytochrome oxidase subunit II
**SCO0930**	-2.2332106	0.02251018	1096353	SCO0930	SCO0930SCM10.18c	lipoprotein
**SCO5910**	-2.2369145	0.02596678	1101352	SCO5910	SCO5910SC10A5.15	hypothetical protein
**SCO2773**	-2.2591878	2.09E-05	1098207	SCO2773	SCO2773SCC105.04c, tesB2	acyl CoA thioesterase II
**SCO0209**	-2.2686921	0.04521813	1095633	SCO0209	SCO0209SCJ12.21	hypothetical protein
**SCO5676**	-2.2848884	0.00011135	1101115	SCO5676	SCO5676gabT	4-aminobutyrate aminotransferase
**SCO1455**	-2.3130886	0.00045559	1096881	SCO1455	SCO1455SCL6.12c	hydrolase
**SCO1140**	-2.3251669	0.00196413	1096563	SCO1140	SCO11402SCG38.33	hypothetical protein
**SCO0172**	-2.3579051	0.00645518	1095596	SCO0172	SCO0172SCJ1.21	hypothetical protein
**SCO6200**	-2.3584895	0.02295048	1101641	SCO6200	SCO6200SC2G5.21c	hypothetical protein
**SCO6198**	-2.3798725	0.01452349	1101639	SCO6198	SCO6198SC2G5.19	hypothetical protein
**SCO3285**	-2.3838474	0.00094967	1098719	SCO3285	SCO3285SCE15.02c	large glycine/alanine rich protein
**SCO7005**	-2.3970192	0.00736406	1102443	SCO7005	SCO7005SC8F11.31	oxidoreductase
**SCO4931**	-2.4196458	0.00603599	1100372	SCO4931	SCO4931SCK13.23	hypothetical protein
**SCO0221**	-2.4203766	0.00909934	1095645	SCO0221	SCO0221SCJ12.33c	pseudo
**SCO2920**	-2.424557	0.00926131	1098353	SCO2920	SCO2920SCE19A.20c	protease
**SCO5531**	-2.438489	0.03588651	1100971	SCO5531	SCO5531SC1C2.12c	hypothetical protein
**SCO1426**	-2.4477108	0.00357054	1096852	SCO1426	SCO1426SC6D7.13c	hypothetical protein
**SCO5667**	-2.457523	0.00321084	1101106	SCO5667	SCO5667SC8B7.11c	ABC transporter substrate-binding protein
**SCO0335**	-2.5212643	9.28E-05	1095758	SCO0335	SCO0335SCF12.14	hypothetical protein
**SCO5671**	-2.5377301	0.00122644	1101110	SCO5671	SCO5671SC8B7.07c	oxidoreductase **CL35 Strakova et al**
**SCO7205**	-2.5545345	0.00151073	1102643	SCO7205	SCO7205SC2H12.04	hydrolase
**SCO0426**	-2.5681194	0.00053011	1095849	SCO0426	SCO0426SCF51A.04	hypothetical protein
**SCO3287**	-2.5854135	0.00067478	1098721	SCO3287	SCO3287SCE15.04	serine/arginine rich protein
**SCO5666**	-2.5889309	0.00543364	1101105	SCO5666	SCO5666SC6A9.01c, SC8B7.12c	gamma-aminobutyraldehyde dehydrogenase

**SCO6007**	-2.5899495	0.004395	1101449	SCO6007	SCO6007SC7b7.04	transmembrane transport protein
**SCO0256**	-2.6018131	0.00702507	1095680	SCO0256	SCO0256SCF20.02	short chain oxidoreductase
**SCO1454**	-2.6137139	2.22E-06	1096880	SCO1454	SCO1454SCL6.11c	amino oxidase
**SCO6196**	-2.6207195	0.00342721	1101637	SCO6196	SCO6196SC2G5.17	AMP-binding domain-containing protein
**SCO3286**	-2.6207248	0.00033637	1098720	SCO3286	SCO3286SCE15.03c	hypothetical protein
**SCO0177**	-2.6338865	0.00384842	1095601	SCO0177	SCO0177SCJ1.26	hypothetical protein
**SCO0198**	-2.6708248	0.00286179	1095622	SCO0198	SCO0198SCJ12.10c	hypothetical protein
**SCO0208**	-2.6836314	0.001454	1095632	SCO0208	SCO0208SCJ12.20	pyruvate phosphate dikinase
**SCO7204**	-2.7186897	0.0011227	1102642	SCO7204	SCO7204SC2H12.03c	hypothetical protein
**SCO0165**	-2.7236501	3.26E-05	1095589	SCO0165	SCO0165SCJ1.14c	hypothetical protein
**SCO6502**	-2.7357936	0.00025518	1101941	SCO6502	SCO6502gvpG	gas vesicle synthesis protein
**SCO7447**	-2.7476314	0.00069955	1102885	SCO7447	SCO7447SC5C11.04c	acetyltranferase
**SCO7473**	-2.7563449	0.03747439	1102911	SCO7473	SCO7473SCBAC17A6.06, paaC	phenylacetic acid degradation protein PaaC
**SCO6197**	-2.7753295	0.00547881	1101638	SCO6197	SCO6197SC2G5.18c	hypothetical protein
**SCO5669**	-2.8319512	0.00115794	1101108	SCO5669	SCO5669SC8B7.09c	polyamine ABC-transporter integral memb rane protein
**SCO2474**	-2.848668	0.02367058	1097908	SCO2474	SCO2474SC7A8.13	metalloproteinase probably secreted
**SCO0166**	-2.8578221	0.00024295	1095590	SCO0166	SCO0166SCJ1.15	**regulator**
**SCO4689**	-2.8596219	0.03752547	1100130	SCO4689	SCO4689SCD31.14	hypothetical protein
**SCO2453**	-2.8709398	0.04036891	1097887	SCO2453	SCO2453SCC24.24	hypothetical protein
**SCO0179**	-2.8791703	0.00226599	1095603	SCO0179	SCO0179SCJ1.28c	zinc-containing dehydrogenase
**SCO5786**	-2.8970565	0.0223686	1101228	SCO5786	SCO5786SC4H2.07c	hydrolase
**SCO0274**	-2.9504152	0.02510314	1095698	SCO0274	SCO0274SCF85.02	alpha-galactosidase
**SCO1137**	-2.9576077	7.88E-05	1096560	SCO1137	SCO11372SCG38.30c	**two component system histidine kinase**
**SCO2011**	-2.9688662	0.00142617	1097445	SCO2011	SCO2011SC7H2.25	branched-chain amino acid ABC transporter ATP-binding protein
**SCO7255**	-2.9954394	0.02974349	1102693	SCO7255	SCO7255SC5H1.37	hypothetical protein
**SCO1459**	-3.0104249	1.63E-05	1096885	SCO1459	SCO1459SCL6.16c	amino acid transporter
**SCO2008**	-3.019596	0.00199129	1097442	SCO2008	SCO2008SC7H2.22	branched-chain amino acid ABC transporter substrate-binding protein
**SCO7242**	-3.0306136	0.03338414	1102680	SCO7242	SCO7242SC7A12.09	hypothetical protein
**SCO7446**	-3.0638414	2.49E-05	1102884	SCO7446	SCO7446SC5C11.03	**regulator**
**SCO1222**	-3.0665127	0.00050732	1096645	SCO1222	SCO12222SCG58.22	hypothetical protein
**SCO4621**	-3.0774142	0.0336443	1100061	SCO4621	SCO4621SCD39.21c, traA1	sporulation-like protein
**SCO5441**	-3.0962338	0.03133934	1100881	SCO5441	SCO5441SC6A11.17c, pep2A	hypothetical protein
**SCO2779**	-3.2523475	1.54E-05	1098213	SCO2779	SCO2779SCC105.10, acdH	acyl-CoA dehydrogenase
**SCO0167**	-3.2872483	7.17E-05	1095591	SCO0167	SCO0167SCJ1.16c	hypothetical protein **CL35 Strakova et al**
**SCO4690**	-3.4117588	8.71E-05	1100131	SCO4690	SCO4690SCD31.15	hypothetical protein
**SCO0205**	-3.4621291	0.00255928	1095629	SCO0205	SCO0205SCJ12.17c	pseudo
**SCO7469**	-3.4697893	0.00046468	1102907	SCO7469	SCO7469SCBAC17A6.02c, paaK	phenylacetate-CoA ligase
**SCO2402**	-3.5939142	0.01327689	1097836	SCO2402	SCO2402SC4A7.30	dehydrogenase
**SCO0168**	-3.5952134	0.00027506	1095592	SCO0168	SCO0168SCJ1.17	**regulator protein CL35 Strakova et al**
**SCO0923**	-3.6880393	4.77E-05	1096346	sdhA	SCO0923SCM10.11c	succinate dehydrogenase flavoprotein subunit
**SCO0181**	-3.7000628	0.00021901	1095605	SCO0181	SCO0181SCJ1.30c	hypothetical protein
**SCO2776**	-3.835056	1.49E-06	1098210	SCO2776	SCO2776SCC105.07, accD1	acetyl/propionyl CoA carboxylase subunit beta
**SCO4938**	-3.8870903	0.02420272	1100379	SCO4938	SCO4938SCK13.30	**ECF-sigma factor**
**SCO0211**	-4.0072317	0.00032479	1095635	SCO0211	SCO0211SCJ12.23	hypothetical protein
**SCO2492**	-4.0254929	0.00038101	1097926	SCO2492	SCO2492SC7A8.31	hypothetical protein
**SCO0174**	-4.108257	1.93E-05	1095598	SCO0174	SCO0174SCJ1.23c	**DNA-binding protein**
**SCO6560**	-4.1102196	0.01983053	1101999	SCO6560	SCO6560SC4B5.10c	respiratory chain oxidoreductase
**SCO1175**	-4.1127909	0.00121496	1096598	SCO1175	SCO1175SCG11A.06c	hypothetical protein

**SCO0215**	-4.169633	1.84E-05	1095639	SCO0215	SCO0215SCJ12.27c	hypothetical protein

Dataset S4: GO analysis
Upregulated enriched:

GOID	Ontology	Term	Level	q	m	t	k	probes	annotations	log_odds_ratio	p
GO:0003674	molecular_function	molecular_function	0	112	3899	7910	175	SCO5525 // SCO5535 // SCO7415 // SCO3706 // SCO2387 // SCO1815 // SCO1335 // SCO2026 // SCO5526 // SCO1814 // SCO5092 // SCO5527 // SCO5515 // SCO2025 // SCO7036 // SCO1233 // SCO2388 // SCO2481 // SCO3104 // SCO6564 // SCO4914 // SCO1577 // SCO5516 // SCO7268 // SCO3127 // SCO4913 // SCO3771 // SCO5554 // SCO4142 // SCO4912 // SCO6172 // SCO1580 // SCO1579 // SCO4049 // SCO7705 // SCO6271 // SCO1570 // SCO1244 // SCO3206 // SCO2210 // SCO5184 // SCO6347 // SCO5584 // SCO0756 // SCO1343 // SCO4921 // SCO5553 // SCO2014 // SCO5522 // SCO3383 // SCO2228 // SCO1487 // SCO5106 // SCO4901 // SCO5425 // SCO6565 // SCO6173 // SCO5860 // SCO1742 // SCO1232 // SCO3964 // SCO1245 // SCO5423 // SCO5467 // SCO3345 // SCO4159 // SCO1243 // SCO2232 // SCO1486 // SCO2093 // SCO2048 // SCO3058 // SCO3311 // SCO7020 // SCO1813 // SCO3756 // SCO5839 // SCO0870 // SCO1578 // SCO1922 // SCO3450 // SCO4962 // SCO1214 // SCO1490 // SCO4038 // SCO0280 // SCO4464 // SCO2094 // SCO1483 // SCO3059 // SCO5424 // SCO0871 // SCO2390 // SCO5583 // SCO7219 // SCO3382 // SCO0872 // SCO7466 // SCO7439 // SCO0249 // SCO0461 // SCO1946 // SCO5564 // SCO5731 // SCO2962 // SCO3148 // SCO5734 // SCO4423 // SCO2051 // SCO3381 // SCO5835 // SCO2455	GO:0005737	0.37671735	0.01161216
GO:0009987	biological_process	cellular process	1	93	2868	7910	175	SCO5525 // SCO5535 // SCO3706 // SCO2387 // SCO1815 // SCO2026 // SCO5526 // SCO1814 // SCO5950 // SCO5092 // SCO5527 // SCO5515 // SCO2025 // SCO7036 // SCO2388 // SCO2481 // SCO3104 // SCO6564 // SCO4914 // SCO1577 // SCO5516 // SCO7268 // SCO3127 // SCO4913 // SCO3771 // SCO5554 // SCO4142 // SCO4912 // SCO6172 // SCO1580 // SCO1579 // SCO4049 // SCO6271 // SCO1570 // SCO1244 // SCO3206 // SCO2210 // SCO5184 // SCO5584 // SCO0756 // SCO1343 // SCO4921 // SCO5553 // SCO2014 // SCO5522 // SCO3383 // SCO1487 // SCO5106 // SCO4901 // SCO5425 // SCO6565 // SCO6173 // SCO5860 // SCO1245 // SCO5423 // SCO5467 // SCO3345 // SCO4159 // SCO1243 // SCO2232 // SCO1486 // SCO2048 // SCO3058 // SCO3311 // SCO1813 // SCO3756 // SCO0870 // SCO1578 // SCO3450 // SCO4962 // SCO1214 // SCO1490 // SCO4038 // SCO4464 // SCO1483 // SCO3059 // SCO5424 // SCO0871 // SCO2390 // SCO5583 // SCO7219 // SCO3382 // SCO0872 // SCO7466 // SCO0249 // SCO1946 // SCO5564 // SCO3148 // SCO5734 // SCO4423 // SCO2051 // SCO3381 // SCO2455	GO:0005737	0.55158037	0.00106157
GO:0003824	molecular_function	catalytic activity	1	90	2821	7910	175	SCO5525 // SCO5535 // SCO7415 // SCO3706 // SCO2387 // SCO1815 // SCO1335 // SCO2026 // SCO5526 // SCO1814 // SCO5092 // SCO5527 // SCO5515 // SCO2025 // SCO7036 // SCO2388 // SCO3104 // SCO6564 // SCO4914 // SCO1577 // SCO7268 // SCO3127 // SCO4913 // SCO3771 // SCO5554 // SCO4912 // SCO6172 // SCO1580 // SCO1579 // SCO4049 // SCO7705 // SCO6271 // SCO1570 // SCO1244 // SCO2210 // SCO5184 // SCO6347 // SCO0756 // SCO1343 // SCO4921 // SCO5553 // SCO2014 // SCO5522 // SCO3383 // SCO2228 // SCO1487 // SCO5106 // SCO4901 // SCO5425 // SCO5860 // SCO1742 // SCO1232 // SCO1245 // SCO5423 // SCO5467 // SCO3345 // SCO1243 // SCO1486 // SCO2093 // SCO2048 // SCO3058 // SCO3311 // SCO7020 // SCO5839 // SCO1578 // SCO1922 // SCO4962 // SCO1214 // SCO4038 // SCO0280 // SCO4464 // SCO2094 // SCO1483 // SCO3059 // SCO5424 // SCO0871 // SCO2390 // SCO7219 // SCO3382 // SCO7439 // SCO0249 // SCO0461 // SCO1946 // SCO5731 // SCO2962 // SCO3148 // SCO4423 // SCO2051 // SCO3381 // SCO2455	GO:0005737	0.528113013	0.00309601
GO:0008152	biological_process	metabolic process	1	102	3638	7910	175	SCO5525 // SCO5535 // SCO7415 // SCO3706 // SCO2387 // SCO1815 // SCO1335 // SCO2026 // SCO5526 // SCO1814 // SCO5092 // SCO5527 // SCO5515 // SCO2025 // SCO7036 // SCO1233 // SCO2388 // SCO2481 // SCO3104 // SCO6564 // SCO4914 // SCO1577 // SCO7268 // SCO3127 // SCO4913 // SCO3771 // SCO5554 // SCO4912 // SCO6172 // SCO1580 // SCO1579 // SCO4049 // SCO7705 // SCO6271 // SCO1570 // SCO1244 // SCO3206 // SCO2210 // SCO5184 // SCO6347 // SCO5584 // SCO0756 // SCO1343 // SCO4921 // SCO5553 // SCO2014 // SCO5522 // SCO3383 // SCO2228 // SCO1487 // SCO5106 // SCO4901 // SCO5425 // SCO6565 // SCO5860 // SCO1742 // SCO1232 // SCO1245 // SCO5423 // SCO5467 // SCO3345 // SCO4159 // SCO1243 // SCO2232 // SCO1486 // SCO2093 // SCO2048 // SCO3058 // SCO3311 // SCO7020 // SCO1813 // SCO3756 // SCO5839 // SCO1578 // SCO1922 // SCO3450 // SCO4962 // SCO1214 // SCO1490 // SCO4038 // SCO0280 // SCO4464 // SCO2094 // SCO1483 // SCO3059 // SCO5424 // SCO0871 // SCO2390 // SCO7219 // SCO3382 // SCO7439 // SCO0249 // SCO0461 // SCO1946 // SCO5564 // SCO5731 // SCO2962 // SCO3148 // SCO4423 // SCO2051 // SCO3381 // SCO2455	GO:0005737	0.341746382	0.07709404
GO:0071704	biological_process	organic substance metabolic process	2	85	2534	7910	175	SCO5525 // SCO5535 // SCO7415 // SCO2387 // SCO1815 // SCO2026 // SCO5526 // SCO1814 // SCO5527 // SCO5515 // SCO2025 // SCO7036 // SCO2388 // SCO2481 // SCO3104 // SCO6564 // SCO4914 // SCO1577 // SCO7268 // SCO3127 // SCO4913 // SCO3771 // SCO5554 // SCO4912 // SCO6172 // SCO1580 // SCO1579 // SCO6271 // SCO1570 // SCO1244 // SCO2210 // SCO5184 // SCO6347 // SCO5584 // SCO0756 // SCO1343 // SCO4921 // SCO5553 // SCO2014 // SCO5522 // SCO3383 // SCO2228 // SCO1487 // SCO4901 // SCO5425 // SCO6565 // SCO5860 // SCO1245 // SCO5423 // SCO5467 // SCO3345 // SCO4159 // SCO1243 // SCO2232 // SCO1486 // SCO2048 // SCO3058 // SCO3311 // SCO7020 // SCO1813 // SCO3756 // SCO5839 // SCO1578 // SCO3450 // SCO4962 // SCO1214 // SCO1490 // SCO4038 // SCO4464 // SCO1483 // SCO3059 // SCO5424 // SCO0871 // SCO2390 // SCO7219 // SCO3382 // SCO7439 // SCO1946 // SCO5564 // SCO5731 // SCO3148 // SCO4423 // SCO2051 // SCO3381 // SCO2455	GO:0005737	0.600440995	0.00106157
GO:0009058	biological_process	biosynthetic process	2	61	1597	7910	175	SCO5535 // SCO2387 // SCO1815 // SCO2026 // SCO1814 // SCO5092 // SCO5527 // SCO5515 // SCO2025 // SCO7036 // SCO2388 // SCO2481 // SCO6564 // SCO1577 // SCO7268 // SCO3771 // SCO5554 // SCO4912 // SCO1580 // SCO1579 // SCO4049 // SCO6271 // SCO1570 // SCO1244 // SCO3206 // SCO2210 // SCO5184 // SCO5584 // SCO4921 // SCO5553 // SCO5522 // SCO3383 // SCO1487 // SCO4901 // SCO5425 // SCO6565 // SCO1245 // SCO3345 // SCO4159 // SCO1243 // SCO2232 // SCO1486 // SCO2048 // SCO3311 // SCO1813 // SCO3756 // SCO1578 // SCO3450 // SCO4962 // SCO1490 // SCO4464 // SCO1483 // SCO3059 // SCO5424 // SCO2390 // SCO3382 // SCO5564 // SCO3148 // SCO2051 // SCO3381 // SCO2455	GO:0000166	0.787839608	0.00112817
GO:0043167	molecular_function	ion binding	2	62	1653	7910	175	SCO5525 // SCO5535 // SCO7415 // SCO3706 // SCO2026 // SCO5526 // SCO5092 // SCO5515 // SCO2025 // SCO7036 // SCO1233 // SCO1577 // SCO7268 // SCO3127 // SCO3771 // SCO4142 // SCO6172 // SCO6271 // SCO1244 // SCO2210 // SCO5184 // SCO5584 // SCO0756 // SCO4921 // SCO5553 // SCO2014 // SCO5522 // SCO3383 // SCO2228 // SCO1487 // SCO5106 // SCO4901 // SCO5860 // SCO1742 // SCO1232 // SCO3964 // SCO1245 // SCO5423 // SCO5467 // SCO3345 // SCO1243 // SCO1486 // SCO2093 // SCO3058 // SCO3311 // SCO7020 // SCO1578 // SCO1922 // SCO4962 // SCO1214 // SCO4038 // SCO0280 // SCO2094 // SCO1483 // SCO5424 // SCO0871 // SCO7466 // SCO1946 // SCO3148 // SCO5734 // SCO4423 // SCO5835	GO:0005737	0.761576169	0.00159874
GO:0044237	biological_process	cellular metabolic process	2	81	2464	7910	175	SCO5525 // SCO5535 // SCO2387 // SCO1815 // SCO2026 // SCO5526 // SCO1814 // SCO5092 // SCO5527 // SCO5515 // SCO2025 // SCO7036 // SCO2388 // SCO2481 // SCO3104 // SCO6564 // SCO4914 // SCO1577 // SCO7268 // SCO3127 // SCO4913 // SCO3771 // SCO5554 // SCO4912 // SCO6172 // SCO1580 // SCO1579 // SCO4049 // SCO6271 // SCO1570 // SCO1244 // SCO3206 // SCO2210 // SCO5184 // SCO5584 // SCO1343 // SCO4921 // SCO5553 // SCO2014 // SCO5522 // SCO3383 // SCO1487 // SCO5106 // SCO4901 // SCO5425 // SCO6565 // SCO5860 // SCO1245 // SCO5423 // SCO3345 // SCO4159 // SCO1243 // SCO2232 // SCO1486 // SCO2048 // SCO3058 // SCO3311 // SCO1813 // SCO3756 // SCO1578 // SCO3450 // SCO4962 // SCO1214 // SCO1490 // SCO4038 // SCO4464 // SCO1483 // SCO3059 // SCO5424 // SCO0871 // SCO2390 // SCO7219 // SCO3382 // SCO0249 // SCO1946 // SCO5564 // SCO3148 // SCO4423 // SCO2051 // SCO3381 // SCO2455	GO:0005737	0.57131433	0.00397412

GO:0044710	biological_process	single-organism metabolic process	2	57	1557	7910	175	SCO5525 // SCO5535 // SCO2387 // SCO1815 // SCO1335 // SCO2026 // SCO5526 // SCO1814 // SCO5092 // SCO5515 // SCO2025 // SCO7036 // SCO2388 // SCO6564 // SCO4914 // SCO1577 // SCO7268 // SCO3127 // SCO4913 // SCO3771 // SCO5554 // SCO4912 // SCO6172 // SCO1580 // SCO1579 // SCO7705 // SCO6271 // SCO1570 // SCO1244 // SCO2210 // SCO4921 // SCO5553 // SCO5522 // SCO3383 // SCO1487 // SCO5106 // SCO4901 // SCO5860 // SCO1245 // SCO3345 // SCO1243 // SCO1486 // SCO2048 // SCO3058 // SCO3311 // SCO1578 // SCO4962 // SCO4464 // SCO1483 // SCO3059 // SCO5424 // SCO2390 // SCO3382 // SCO0249 // SCO3148 // SCO2051 // SCO3381	GO:0005737	0.726587653	0.00773984
GO:0044238	biological_process	primary metabolic process	2	73	2273	7910	175	SCO5535 // SCO7415 // SCO2387 // SCO1815 // SCO2026 // SCO1814 // SCO5515 // SCO2025 // SCO7036 // SCO2388 // SCO2481 // SCO3104 // SCO6564 // SCO4914 // SCO1577 // SCO7268 // SCO3127 // SCO5554 // SCO4912 // SCO6172 // SCO1580 // SCO1579 // SCO6271 // SCO1570 // SCO2210 // SCO5184 // SCO6347 // SCO5584 // SCO0756 // SCO1343 // SCO4921 // SCO5553 // SCO2014 // SCO5522 // SCO3383 // SCO2228 // SCO1487 // SCO5106 // SCO4901 // SCO6565 // SCO5860 // SCO5423 // SCO5467 // SCO3345 // SCO4159 // SCO2232 // SCO1486 // SCO2048 // SCO3058 // SCO7020 // SCO1813 // SCO3756 // SCO1578 // SCO3450 // SCO4962 // SCO1214 // SCO1490 // SCO4038 // SCO4464 // SCO1483 // SCO3059 // SCO0871 // SCO2390 // SCO7219 // SCO3382 // SCO7439 // SCO1946 // SCO5564 // SCO5731 // SCO3148 // SCO4423 // SCO2051 // SCO3381	GO:0000166	0.537693458	0.02884059
GO:0006807	biological_process	nitrogen compound metabolic process	2	55	1567	7910	175	SCO5525 // SCO2026 // SCO5526 // SCO5527 // SCO5515 // SCO2025 // SCO7036 // SCO1233 // SCO2481 // SCO3104 // SCO4914 // SCO1577 // SCO7268 // SCO3771 // SCO5554 // SCO6172 // SCO1580 // SCO1579 // SCO1570 // SCO1244 // SCO2210 // SCO5184 // SCO5584 // SCO1343 // SCO5553 // SCO5522 // SCO3383 // SCO1487 // SCO4901 // SCO6565 // SCO1232 // SCO1245 // SCO3345 // SCO4159 // SCO1243 // SCO2232 // SCO1486 // SCO2048 // SCO3058 // SCO3311 // SCO1813 // SCO3756 // SCO5839 // SCO1578 // SCO3450 // SCO4962 // SCO1490 // SCO4038 // SCO4464 // SCO1483 // SCO3059 // SCO3382 // SCO2051 // SCO3381 // SCO2455	GO:0005737	0.665821117	0.02985799
GO:0044281	biological_process	small molecule metabolic process	3	48	842	7910	175	SCO5525 // SCO5535 // SCO2387 // SCO1815 // SCO2026 // SCO5526 // SCO1814 // SCO5515 // SCO2025 // SCO7036 // SCO2388 // SCO6564 // SCO4914 // SCO1577 // SCO7268 // SCO3127 // SCO5554 // SCO6172 // SCO1580 // SCO1579 // SCO6271 // SCO1570 // SCO1244 // SCO2210 // SCO4921 // SCO5553 // SCO5522 // SCO3383 // SCO1487 // SCO4901 // SCO5860 // SCO1245 // SCO3345 // SCO1243 // SCO1486 // SCO2048 // SCO3058 // SCO1578 // SCO4962 // SCO4464 // SCO1483 // SCO3059 // SCO5424 // SCO2390 // SCO3382 // SCO3148 // SCO2051 // SCO3381	GO:0005737	1.365536945	4.53E-07
GO:0044711	biological_process	single-organism biosynthetic process	3	31	400	7910	175	SCO5535 // SCO2387 // SCO1815 // SCO2026 // SCO1814 // SCO5515 // SCO2025 // SCO7036 // SCO2388 // SCO6564 // SCO1577 // SCO5554 // SCO1580 // SCO1579 // SCO6271 // SCO1570 // SCO1244 // SCO2210 // SCO4921 // SCO5553 // SCO5522 // SCO3383 // SCO1245 // SCO3345 // SCO1243 // SCO2048 // SCO1578 // SCO4962 // SCO1483 // SCO2390 // SCO2051	GO:0000166	1.808590988	6.81E-07
GO:1901564	biological_process	organonitrogen compound metabolic p	3	40	646	7910	175	SCO5525 // SCO2026 // SCO5526 // SCO5527 // SCO5515 // SCO2025 // SCO7036 // SCO4914 // SCO1577 // SCO7268 // SCO3771 // SCO5554 // SCO6172 // SCO1580 // SCO1579 // SCO1570 // SCO1244 // SCO2210 // SCO5553 // SCO5522 // SCO3383 // SCO1487 // SCO4901 // SCO1245 // SCO3345 // SCO1243 // SCO1486 // SCO2048 // SCO3058 // SCO3311 // SCO5839 // SCO1578 // SCO4962 // SCO4464 // SCO1483 // SCO3059 // SCO3382 // SCO2051 // SCO3381 // SCO2455	GO:0005737	1.484788608	1.05E-06
GO:0044249	biological_process	cellular biosynthetic process	3	61	1539	7910	175	SCO5535 // SCO2387 // SCO1815 // SCO2026 // SCO1814 // SCO5092 // SCO5527 // SCO5515 // SCO2025 // SCO7036 // SCO2388 // SCO2481 // SCO6564 // SCO1577 // SCO7268 // SCO3771 // SCO5554 // SCO4912 // SCO1580 // SCO1579 // SCO4049 // SCO6271 // SCO1570 // SCO1244 // SCO3206 // SCO2210 // SCO5184 // SCO5584 // SCO4921 // SCO5553 // SCO5522 // SCO3383 // SCO1487 // SCO4901 // SCO5425 // SCO6565 // SCO1245 // SCO3345 // SCO4159 // SCO1243 // SCO2232 // SCO1486 // SCO2048 // SCO3311 // SCO1813 // SCO3756 // SCO1578 // SCO3450 // SCO4962 // SCO1490 // SCO4464 // SCO1483 // SCO3059 // SCO5424 // SCO2390 // SCO3382 // SCO5564 // SCO3148 // SCO2051 // SCO3381 // SCO2455	GO:0000166	0.841210689	0.00035896
GO:1901576	biological_process	organic substance biosynthetic process	3	57	1498	7910	175	SCO5535 // SCO2387 // SCO1815 // SCO2026 // SCO1814 // SCO5527 // SCO5515 // SCO2025 // SCO7036 // SCO2388 // SCO2481 // SCO6564 // SCO1577 // SCO7268 // SCO3771 // SCO5554 // SCO1580 // SCO1579 // SCO6271 // SCO1570 // SCO1244 // SCO2210 // SCO5184 // SCO5584 // SCO4921 // SCO5553 // SCO5522 // SCO3383 // SCO1487 // SCO4901 // SCO5425 // SCO6565 // SCO1245 // SCO3345 // SCO4159 // SCO1243 // SCO2232 // SCO1486 // SCO2048 // SCO3311 // SCO1813 // SCO3756 // SCO1578 // SCO3450 // SCO4962 // SCO1490 // SCO4464 // SCO1483 // SCO3059 // SCO5424 // SCO2390 // SCO3382 // SCO5564 // SCO3148 // SCO2051 // SCO3381 // SCO2455	GO:0000166	0.782318973	0.00289885
GO:0005622	cellular_component	intracellular	3	42	1086	7910	175	SCO5525 // SCO5535 // SCO2387 // SCO2026 // SCO5526 // SCO2025 // SCO7036 // SCO1233 // SCO2388 // SCO6564 // SCO4914 // SCO1577 // SCO7268 // SCO3771 // SCO5554 // SCO4912 // SCO1580 // SCO1579 // SCO7705 // SCO1570 // SCO2210 // SCO5184 // SCO6347 // SCO1343 // SCO5522 // SCO3383 // SCO2228 // SCO4901 // SCO5425 // SCO1232 // SCO1245 // SCO2048 // SCO3756 // SCO1578 // SCO1214 // SCO5424 // SCO7219 // SCO3382 // SCO1946 // SCO5564 // SCO5731 // SCO2051	GO:0005737	0.805759903	0.02985799
GO:0016810	molecular_function	hydrolase activity, acting on carbon-nit	3	9	95	7910	175	SCO5525 // SCO5526 // SCO5527 // SCO7268 // SCO4049 // SCO4901 // SCO1486 // SCO4038 // SCO4464	GO:0005737	2.098320261	0.04061661
GO:0043168	molecular_function	anion binding	3	38	979	7910	175	SCO5535 // SCO3706 // SCO5092 // SCO5515 // SCO2025 // SCO7036 // SCO1577 // SCO3771 // SCO4142 // SCO6271 // SCO2210 // SCO5184 // SCO5584 // SCO0756 // SCO4921 // SCO2014 // SCO3383 // SCO1487 // SCO1742 // SCO1232 // SCO1245 // SCO5423 // SCO1243 // SCO3058 // SCO1578 // SCO1922 // SCO4962 // SCO1214 // SCO2094 // SCO1483 // SCO5424 // SCO0871 // SCO7466 // SCO1946 // SCO3148 // SCO5734 // SCO4423 // SCO5835	GO:0000166	0.811013331	0.0526016
GO:0043169	molecular_function	cation binding	3	34	868	7910	175	SCO5525 // SCO7415 // SCO2026 // SCO5526 // SCO2025 // SCO1233 // SCO7268 // SCO3127 // SCO6172 // SCO6271 // SCO1244 // SCO4921 // SCO5553 // SCO2014 // SCO5522 // SCO2228 // SCO5106 // SCO4901 // SCO5860 // SCO1232 // SCO3964 // SCO5423 // SCO5467 // SCO3345 // SCO1486 // SCO2093 // SCO3058 // SCO3311 // SCO7020 // SCO1214 // SCO4038 // SCO0280 // SCO1483 // SCO5424	GO:0005737	0.824162476	0.07429049
GO:0044283	biological_process	small molecule biosynthetic process	4	31	363	7910	175	SCO5535 // SCO2387 // SCO1815 // SCO2026 // SCO1814 // SCO5515 // SCO2025 // SCO7036 // SCO2388 // SCO6564 // SCO1577 // SCO5554 // SCO1580 // SCO1579 // SCO6271 // SCO1570 // SCO1244 // SCO2210 // SCO4921 // SCO5553 // SCO5522 // SCO3383 // SCO1245 // SCO3345 // SCO1243 // SCO2048 // SCO1578 // SCO4962 // SCO1483 // SCO2390 // SCO2051	GO:0000166	1.94862144	1.37E-07
GO:1901566	biological_process	organonitrogen compound biosyntheti	4	34	439	7910	175	SCO2026 // SCO5527 // SCO5515 // SCO2025 // SCO7036 // SCO1577 // SCO7268 // SCO3771 // SCO5554 // SCO1580 // SCO1579 // SCO1570 // SCO1244 // SCO2210 // SCO5553 // SCO5522 // SCO3383 // SCO1487 // SCO4901 // SCO1245 // SCO3345 // SCO1243 // SCO1486 // SCO2048 // SCO3311 // SCO1578 // SCO4962 // SCO4464 // SCO1483 // SCO3059 // SCO3382 // SCO2051 // SCO3381 // SCO2455	GO:0003824	1.807636579	1.55E-07
GO:0006082	biological_process	organic acid metabolic process	4	36	531	7910	175	SCO5535 // SCO2387 // SCO1815 // SCO2026 // SCO1814 // SCO5515 // SCO2025 // SCO7036 // SCO2388 // SCO6564 // SCO1577 // SCO3127 // SCO5554 // SCO1580 // SCO1579 // SCO6271 // SCO1570 // SCO1244 // SCO2210 // SCO4921 // SCO5553 // SCO5522 // SCO3383 // SCO1487 // SCO1245 // SCO3345 // SCO1243 // SCO2048 // SCO3058 // SCO1578 // SCO4962 // SCO1483 // SCO5424 // SCO2390 // SCO3148 // SCO2051	GO:0000166	1.615607818	8.31E-07
GO:0044424	cellular_component	intracellular part	4	42	1025	7910	175	SCO5525 // SCO5535 // SCO2387 // SCO2026 // SCO5526 // SCO2025 // SCO7036 // SCO1233 // SCO2388 // SCO6564 // SCO4914 // SCO1577 // SCO7268 // SCO3771 // SCO5554 // SCO4912 // SCO1580 // SCO1579 // SCO7705 // SCO1570 // SCO2210 // SCO5184 // SCO6347 // SCO1343 // SCO5522 // SCO3383 // SCO2228 // SCO4901 // SCO5425 // SCO1232 // SCO1245 // SCO2048 // SCO3756 // SCO1578 // SCO1214 // SCO5424 // SCO7219 // SCO3382 // SCO1946 // SCO5564 // SCO5731 // SCO2051	GO:0005737	0.889160096	0.00955875

GO:0016774	molecular_function	phosphotransferase activity, carboxyl g	4	3	5	7910	175	SCO1578 // SCO5424 // SCO1946	GO:0000166	4.761285273	0.02218626
GO:1901658	biological_process	glycosyl compound catabolic process	4	3	9	7910	175	SCO4914 // SCO7268 // SCO4901	GO:0003824	3.913288367	0.09255808
GO:0046872	molecular_function	metal ion binding	4	33	851	7910	175	SCO5525 // SCO7415 // SCO2026 // SCO5526 // SCO2025 // SCO1233 // SCO7268 // SCO3127 // SCO6172 // SCO6271 // SCO1244 // SCO4921 // SCO5553 // SCO2014 // SCO5522 // SCO5106 // SCO4901 // SCO5860 // SCO1232 // SCO3964 // SCO5423 // SCO5467 // SCO3345 // SCO1486 // SCO2093 // SCO3058 // SCO3311 // SCO7020 // SCO1214 // SCO4038 // SCO0280 // SCO1483 // SCO5424	GO:0005737	0.809629665	0.09841731
GO:0016053	biological_process	organic acid biosynthetic process	5	31	276	7910	175	SCO5535 // SCO2387 // SCO1815 // SCO2026 // SCO1814 // SCO5515 // SCO2025 // SCO7036 // SCO2388 // SCO6564 // SCO1577 // SCO5554 // SCO1580 // SCO1579 // SCO6271 // SCO1570 // SCO1244 // SCO2210 // SCO4921 // SCO5553 // SCO5522 // SCO3383 // SCO1245 // SCO3345 // SCO1243 // SCO2048 // SCO1578 // SCO4962 // SCO1483 // SCO2390 // SCO2051	GO:0000166	2.343922721	1.36E-10
GO:0043436	biological_process	oxoacid metabolic process	5	35	516	7910	175	SCO5535 // SCO2387 // SCO1815 // SCO2026 // SCO1814 // SCO5515 // SCO2025 // SCO7036 // SCO2388 // SCO6564 // SCO1577 // SCO3127 // SCO5554 // SCO1580 // SCO1579 // SCO6271 // SCO1570 // SCO1244 // SCO2210 // SCO4921 // SCO5553 // SCO5522 // SCO3383 // SCO1487 // SCO1245 // SCO3345 // SCO1243 // SCO2048 // SCO3058 // SCO1578 // SCO4962 // SCO1483 // SCO2390 // SCO3148 // SCO2051	GO:0000166	1.616306629	1.18E-06
GO:0005737	cellular_component	cytoplasm	5	42	984	7910	175	SCO5525 // SCO5535 // SCO2387 // SCO2026 // SCO5526 // SCO2025 // SCO7036 // SCO1233 // SCO2388 // SCO6564 // SCO4914 // SCO1577 // SCO7268 // SCO3771 // SCO5554 // SCO4912 // SCO1580 // SCO1579 // SCO7705 // SCO1570 // SCO2210 // SCO5184 // SCO6347 // SCO1343 // SCO5522 // SCO3383 // SCO2228 // SCO4901 // SCO5425 // SCO1232 // SCO1245 // SCO2048 // SCO3756 // SCO1578 // SCO1214 // SCO5424 // SCO7219 // SCO3382 // SCO1946 // SCO5564 // SCO5731 // SCO2051	GO:0005737	0.948053785	0.00392468
GO:0004312	molecular_function	fatty acid synthase activity	5	5	25	7910	175	SCO2387 // SCO1815 // SCO1814 // SCO2388 // SCO6564	GO:0003824	3.176322773	0.03412824
GO:0019752	biological_process	carboxylic acid metabolic process	6	35	492	7910	175	SCO5535 // SCO2387 // SCO1815 // SCO2026 // SCO1814 // SCO5515 // SCO2025 // SCO7036 // SCO2388 // SCO6564 // SCO1577 // SCO3127 // SCO5554 // SCO1580 // SCO1579 // SCO6271 // SCO1570 // SCO1244 // SCO2210 // SCO4921 // SCO5553 // SCO5522 // SCO3383 // SCO1487 // SCO1245 // SCO3345 // SCO1243 // SCO2048 // SCO3058 // SCO1578 // SCO4962 // SCO1483 // SCO2390 // SCO3148 // SCO2051	GO:0000166	1.685019379	5.44E-07
GO:0016151	molecular_function	nickel cation binding	6	4	13	7910	175	SCO5525 // SCO5526 // SCO1233 // SCO1232	GO:0005737	3.797811149	0.02884059
GO:0046394	biological_process	carboxylic acid biosynthetic process	7	31	268	7910	175	SCO5535 // SCO2387 // SCO1815 // SCO2026 // SCO1814 // SCO5515 // SCO2025 // SCO7036 // SCO2388 // SCO6564 // SCO1577 // SCO5554 // SCO1580 // SCO1579 // SCO6271 // SCO1570 // SCO1244 // SCO2210 // SCO4921 // SCO5553 // SCO5522 // SCO3383 // SCO1245 // SCO3345 // SCO1243 // SCO2048 // SCO1578 // SCO4962 // SCO1483 // SCO2390 // SCO2051	GO:0000166	2.386357987	1.20E-10
GO:0006520	biological_process	cellular amino acid metabolic process	7	21	281	7910	175	SCO2026 // SCO5515 // SCO2025 // SCO7036 // SCO1577 // SCO5554 // SCO1580 // SCO1579 // SCO1570 // SCO2210 // SCO5553 // SCO5522 // SCO3383 // SCO1487 // SCO3345 // SCO2048 // SCO3058 // SCO1578 // SCO4962 // SCO1483 // SCO2051	GO:0003824	1.75614197	0.00035167
GO:0009164	biological_process	nucleoside catabolic process	7	3	7	7910	175	SCO4914 // SCO7268 // SCO4901	GO:0003824	4.275858446	0.0526016
GO:0009123	biological_process	nucleoside monophosphate metabolic	7	6	44	7910	175	SCO7268 // SCO1487 // SCO4901 // SCO1486 // SCO1483 // SCO3059	GO:0004000	2.62378175	0.05586605
GO:0032787	biological_process	monocarboxylic acid metabolic process	7	13	199	7910	175	SCO5535 // SCO2387 // SCO1815 // SCO1814 // SCO2388 // SCO6564 // SCO6271 // SCO1244 // SCO4921 // SCO1245 // SCO1243 // SCO2390 // SCO3148	GO:0000166	1.562065965	0.06217289
GO:1901293	biological_process	nucleoside phosphate biosynthetic pro	7	9	109	7910	175	SCO7268 // SCO1487 // SCO4901 // SCO1486 // SCO4464 // SCO1483 // SCO3059 // SCO3382 // SCO3381	GO:0004000	1.899991544	0.07777877
GO:0008652	biological_process	cellular amino acid biosynthetic proces	8	19	179	7910	175	SCO2026 // SCO5515 // SCO2025 // SCO7036 // SCO1577 // SCO5554 // SCO1580 // SCO1579 // SCO1570 // SCO2210 // SCO5553 // SCO5522 // SCO3383 // SCO3345 // SCO2048 // SCO1578 // SCO4962 // SCO1483 // SCO2051	GO:0003824	2.262362604	6.87E-06
GO:1901605	biological_process	alpha-amino acid metabolic process	8	19	211	7910	175	SCO2026 // SCO5515 // SCO2025 // SCO7036 // SCO1577 // SCO5554 // SCO1580 // SCO1579 // SCO1570 // SCO2210 // SCO5553 // SCO5522 // SCO3345 // SCO2048 // SCO3058 // SCO1578 // SCO4962 // SCO1483 // SCO2051	GO:0003824	2.025079192	8.81E-05
GO:0072330	biological_process	monocarboxylic acid biosynthetic proce	8	12	82	7910	175	SCO5535 // SCO2387 // SCO1815 // SCO1814 // SCO2388 // SCO6564 // SCO6271 // SCO1244 // SCO4921 // SCO1245 // SCO1243 // SCO2390	GO:0000166	2.725661364	9.58E-05
GO:0009064	biological_process	glutamine family amino acid metabolic	8	11	69	7910	175	SCO2026 // SCO2025 // SCO7036 // SCO1577 // SCO1580 // SCO1579 // SCO1570 // SCO2210 // SCO1578 // SCO1483 // SCO2051	GO:0003824	2.849158029	0.00012057
GO:0009081	biological_process	branched-chain amino acid metabolic p	8	5	25	7910	175	SCO5554 // SCO5553 // SCO5522 // SCO3345 // SCO4962	GO:0003861	3.176322773	0.03412824
GO:0009161	biological_process	ribonucleoside monophosphate metab	8	6	40	7910	175	SCO7268 // SCO1487 // SCO4901 // SCO1486 // SCO1483 // SCO3059	GO:0004000	2.761285273	0.03876423
GO:0009124	biological_process	nucleoside monophosphate biosynthet	8	6	43	7910	175	SCO7268 // SCO1487 // SCO4901 // SCO1486 // SCO1483 // SCO3059	GO:0004000	2.656948614	0.0526016
GO:0006768	biological_process	biotin metabolic process	8	3	8	7910	175	SCO1244 // SCO1245 // SCO1243	GO:0003824	4.083213368	0.06899586
GO:0006631	biological_process	fatty acid metabolic process	8	9	108	7910	175	SCO5535 // SCO2387 // SCO1815 // SCO1814 // SCO2388 // SCO6564 // SCO6271 // SCO4921 // SCO2390	GO:0000166	1.913288367	0.0750615
GO:0009084	biological_process	glutamine family amino acid biosynthe	9	10	35	7910	175	SCO2026 // SCO2025 // SCO7036 // SCO1577 // SCO1580 // SCO1579 // SCO1570 // SCO2210 // SCO1578 // SCO1483	GO:0003824	3.690895945	1.69E-06
GO:1901607	biological_process	alpha-amino acid biosynthetic process	9	18	150	7910	175	SCO2026 // SCO5515 // SCO2025 // SCO7036 // SCO1577 // SCO5554 // SCO1580 // SCO1579 // SCO1570 // SCO2210 // SCO5553 // SCO5522 // SCO3345 // SCO2048 // SCO1578 // SCO4962 // SCO1483 // SCO2051	GO:0003824	2.439357178	2.68E-06
GO:0006525	biological_process	arginine metabolic process	9	7	22	7910	175	SCO7036 // SCO1577 // SCO1580 // SCO1579 // SCO1570 // SCO1578 // SCO1483	GO:0000166	3.846174171	0.00012416
GO:0006633	biological_process	fatty acid biosynthetic process	9	9	64	7910	175	SCO5535 // SCO2387 // SCO1815 // SCO1814 // SCO2388 // SCO6564 // SCO6271 // SCO4921 // SCO2390	GO:0000166	2.668175869	0.00289885
GO:0009082	biological_process	branched-chain amino acid biosyntheti	9	5	19	7910	175	SCO5554 // SCO5553 // SCO5522 // SCO3345 // SCO4962	GO:0003861	3.572251449	0.01033554
GO:0009156	biological_process	ribonucleoside monophosphate biosyn	9	6	39	7910	175	SCO7268 // SCO1487 // SCO4901 // SCO1486 // SCO1483 // SCO3059	GO:0004000	2.797811149	0.03435746
GO:0009102	biological_process	biotin biosynthetic process	9	3	7	7910	175	SCO1244 // SCO1245 // SCO1243	GO:0003824	4.275858446	0.0526016
GO:0006551	biological_process	leucine metabolic process	9	3	9	7910	175	SCO5554 // SCO5553 // SCO5522	GO:0003861	3.913288367	0.09255808
GO:0006526	biological_process	arginine biosynthetic process	10	7	15	7910	175	SCO7036 // SCO1577 // SCO1580 // SCO1579 // SCO1570 // SCO1578 // SCO1483	GO:0000166	4.398715194	6.87E-06
GO:0009098	biological_process	leucine biosynthetic process	10	3	7	7910	175	SCO5554 // SCO5553 // SCO5522	GO:0003861	4.275858446	0.0526016
GO:0044205	biological_process	'de novo' UMP biosynthetic process	12	3	7	7910	175	SCO1487 // SCO1486 // SCO1483	GO:0004070	4.275858446	0.0526016

Down-regulated enriched

GOID	Ontology	Term	Level	q	m	t	k	probes	annotations	log_odds_ratio	p
GO:0051179	biological_process	localization	1	57	695	7910	398	SCO3484 // SCO4964 // SCO6065 // SCO1887 // SCO5783 // SCO0079 // SCO7012 // SCO6814 // SCO7013 // SCO5035 // SCO7555 // SCO5782 // SCO5258 // SCO0322 // SCO7544 // SCO4564 // SCO7410 // SCO7197 // SCO0137 // SCO1218 // SCO5259 // SCO1147 // SCO3667 // SCO0533 // SCO5429 // SCO7028 // SCO0952 // SCO6011 // SCO5260 // SCO5229 // SCO3958 // SCO6010 // SCO1056 // SCO0540 // SCO6009 // SCO2746 // SCO2829 // SCO2831 // SCO2830 // SCO2828 // SCO1225 // SCO2978 // SCO0273 // SCO2980 // SCO3090 // SCO7596 // SCO5911 // SCO5430 // SCO4562 // SCO4563 // SCO2009 // SCO5667 // SCO6007 // SCO5669 // SCO2011 // SCO1459 // SCO2008	GO:0005215	0.704858205	0.05432081
GO:0051234	biological_process	establishment of localization	2	57	693	7910	398	SCO3484 // SCO4964 // SCO6065 // SCO1887 // SCO5783 // SCO0079 // SCO7012 // SCO6814 // SCO7013 // SCO5035 // SCO7555 // SCO5782 // SCO5258 // SCO0322 // SCO7544 // SCO4564 // SCO7410 // SCO7197 // SCO0137 // SCO1218 // SCO5259 // SCO1147 // SCO3667 // SCO0533 // SCO5429 // SCO7028 // SCO0952 // SCO6011 // SCO5260 // SCO5229 // SCO3958 // SCO6010 // SCO1056 // SCO0540 // SCO6009 // SCO2746 // SCO2829 // SCO2831 // SCO2830 // SCO2828 // SCO1225 // SCO2978 // SCO0273 // SCO2980 // SCO3090 // SCO7596 // SCO5911 // SCO5430 // SCO4562 // SCO4563 // SCO2009 // SCO5667 // SCO6007 // SCO5669 // SCO2011 // SCO1459 // SCO2008	GO:0005215	0.709015831	0.05432081
GO:0042597	cellular_component	periplasmic space	3	17	83	7910	398	SCO3484 // SCO6065 // SCO1444 // SCO2383 // SCO7555 // SCO3667 // SCO0952 // SCO5260 // SCO1056 // SCO6009 // SCO2828 // SCO2978 // SCO0273 // SCO0458 // SCO5911 // SCO5667 // SCO2008	GO:0005215	2.025260769	0.0018004
GO:0006810	biological_process	transport	3	57	693	7910	398	SCO3484 // SCO4964 // SCO6065 // SCO1887 // SCO5783 // SCO0079 // SCO7012 // SCO6814 // SCO7013 // SCO5035 // SCO7555 // SCO5782 // SCO5258 // SCO0322 // SCO7544 // SCO4564 // SCO7410 // SCO7197 // SCO0137 // SCO1218 // SCO5259 // SCO1147 // SCO3667 // SCO0533 // SCO5429 // SCO7028 // SCO0952 // SCO6011 // SCO5260 // SCO5229 // SCO3958 // SCO6010 // SCO1056 // SCO0540 // SCO6009 // SCO2746 // SCO2829 // SCO2831 // SCO2830 // SCO2828 // SCO1225 // SCO2978 // SCO0273 // SCO2980 // SCO3090 // SCO7596 // SCO5911 // SCO5430 // SCO4562 // SCO4563 // SCO2009 // SCO5667 // SCO6007 // SCO5669 // SCO2011 // SCO1459 // SCO2008	GO:0005215	0.709015831	0.05432081

GO:0044712	biological_process	single-organism catabolic process	3	19	151	7910	398	SCO6789 // SCO4869 // SCO1895 // SCO2469 // SCO6732 // SCO6027 // SCO6730 // SCO6968 // SCO2999 // SCO3247 // SCO1838 // SCO2401 // SCO2774 // SCO2773 // SCO5676 // SCO0256 // SCO7473 // SCO7469 // SCO2776	GO:0003824	1.322360133	0.06922117
GO:0071702	biological_process	organic substance transport	4	31	261	7910	398	SCO1887 // SCO5035 // SCO7555 // SCO5258 // SCO7410 // SCO7197 // SCO0137 // SCO1147 // SCO3667 // SCO0952 // SCO6011 // SCO5260 // SCO5229 // SCO6010 // SCO0540 // SCO6009 // SCO2746 // SCO2829 // SCO2831 // SCO2830 // SCO2828 // SCO1225 // SCO2978 // SCO2980 // SCO5911 // SCO5430 // SCO2009 // SCO5667 // SCO2011 // SCO1459 // SCO2008	GO:0005886	1.239127673	0.00751505
GO:0016042	biological_process	lipid catabolic process	4	11	48	7910	398	SCO6789 // SCO4869 // SCO2469 // SCO6732 // SCO6027 // SCO6730 // SCO6968 // SCO3247 // SCO1838 // SCO2774 // SCO2773	GO:0003824	2.187306477	0.01627492
GO:0044282	biological_process	small molecule catabolic process	4	19	151	7910	398	SCO6789 // SCO4869 // SCO1895 // SCO2469 // SCO6732 // SCO6027 // SCO6730 // SCO6968 // SCO2999 // SCO3247 // SCO1838 // SCO2401 // SCO2774 // SCO2773 // SCO5676 // SCO0256 // SCO7473 // SCO7469 // SCO2776	GO:0003824	1.322360133	0.06922117
GO:0044242	biological_process	cellular lipid catabolic process	5	11	34	7910	398	SCO6789 // SCO4869 // SCO2469 // SCO6732 // SCO6027 // SCO6730 // SCO6968 // SCO3247 // SCO1838 // SCO2774 // SCO2773	GO:0003824	2.684806136	0.0018004
GO:0030258	biological_process	lipid modification	5	9	33	7910	398	SCO6789 // SCO4869 // SCO6732 // SCO6027 // SCO6730 // SCO6968 // SCO3247 // SCO1838 // SCO2774	GO:0003824	2.438368241	0.0178864
GO:0015849	biological_process	organic acid transport	5	13	69	7910	398	SCO5258 // SCO7197 // SCO5260 // SCO5229 // SCO2829 // SCO2831 // SCO2830 // SCO2828 // SCO1225 // SCO2009 // SCO2011 // SCO1459 // SCO2008	GO:0000166	1.90475262	0.02006722
GO:0016054	biological_process	organic acid catabolic process	5	17	121	7910	398	SCO6789 // SCO4869 // SCO1895 // SCO6732 // SCO6027 // SCO6730 // SCO6968 // SCO2999 // SCO3247 // SCO1838 // SCO2401 // SCO2774 // SCO2773 // SCO5676 // SCO7473 // SCO7469 // SCO2776	GO:0003824	1.481436963	0.05432081
GO:0008643	biological_process	carbohydrate transport	5	13	80	7910	398	SCO1887 // SCO7555 // SCO7410 // SCO0137 // SCO3667 // SCO6011 // SCO6010 // SCO0540 // SCO6009 // SCO2746 // SCO2978 // SCO2980 // SCO5430	GO:0005886	1.691348982	0.06177801
GO:0034440	biological_process	lipid oxidation	6	9	24	7910	398	SCO6789 // SCO4869 // SCO6732 // SCO6027 // SCO6730 // SCO6968 // SCO3247 // SCO1838 // SCO2774	GO:0003824	2.89779986	0.00196951
GO:0006820	biological_process	anion transport	6	16	97	7910	398	SCO6814 // SCO5258 // SCO7197 // SCO0952 // SCO5260 // SCO5229 // SCO2829 // SCO2831 // SCO2830 // SCO2828 // SCO1225 // SCO7596 // SCO2009 // SCO2011 // SCO1459 // SCO2008	GO:0000166	1.712924517	0.0178864
GO:0015711	biological_process	organic anion transport	7	14	72	7910	398	SCO5258 // SCO7197 // SCO0952 // SCO5260 // SCO5229 // SCO2829 // SCO2831 // SCO2830 // SCO2828 // SCO1225 // SCO2009 // SCO2011 // SCO1459 // SCO2008	GO:0000166	1.950267279	0.0102471
GO:0046395	biological_process	carboxylic acid catabolic process	7	17	121	7910	398	SCO6789 // SCO4869 // SCO1895 // SCO6732 // SCO6027 // SCO6730 // SCO6968 // SCO2999 // SCO3247 // SCO1838 // SCO2401 // SCO2774 // SCO2773 // SCO5676 // SCO7473 // SCO7469 // SCO2776	GO:0003824	1.481436963	0.05432081
GO:0032787	biological_process	monocarboxylic acid metabolic process	7	23	199	7910	398	SCO6789 // SCO5385 // SCO4869 // SCO2561 // SCO6732 // SCO3563 // SCO6027 // SCO6730 // SCO0815 // SCO6968 // SCO1346 // SCO4006 // SCO7329 // SCO3247 // SCO1838 // SCO2401 // SCO2774 // SCO6475 // SCO2773 // SCO5676 // SCO0208 // SCO7473 // SCO7469	GO:0003824	1.199774694	0.06177801
GO:0072329	biological_process	monocarboxylic acid catabolic process	8	14	63	7910	398	SCO6789 // SCO4869 // SCO6732 // SCO6027 // SCO6730 // SCO6968 // SCO3247 // SCO1838 // SCO2401 // SCO2774 // SCO2773 // SCO5676 // SCO7473 // SCO7469	GO:0003824	2.142912357	0.00277394
GO:0046942	biological_process	carboxylic acid transport	8	13	69	7910	398	SCO5258 // SCO7197 // SCO5260 // SCO5229 // SCO2829 // SCO2831 // SCO2830 // SCO2828 // SCO1225 // SCO2009 // SCO2011 // SCO1459 // SCO2008	GO:0000166	1.90475262	0.02006722
GO:0006631	biological_process	fatty acid metabolic process	8	16	108	7910	398	SCO6789 // SCO5385 // SCO4869 // SCO2561 // SCO6732 // SCO6027 // SCO6730 // SCO6968 // SCO1346 // SCO4006 // SCO7329 // SCO3247 // SCO1838 // SCO2774 // SCO6475 // SCO2773	GO:0003824	1.557949857	0.05257308
GO:0009062	biological_process	fatty acid catabolic process	9	10	27	7910	398	SCO6789 // SCO4869 // SCO6732 // SCO6027 // SCO6730 // SCO6968 // SCO3247 // SCO1838 // SCO2774 // SCO2773	GO:0003824	2.879877952	0.0018004
GO:0019395	biological_process	fatty acid oxidation	9	9	24	7910	398	SCO6789 // SCO4869 // SCO6732 // SCO6027 // SCO6730 // SCO6968 // SCO3247 // SCO1838 // SCO2774	GO:0003824	2.89779986	0.00196951
GO:0006865	biological_process	amino acid transport	9	13	61	7910	398	SCO5258 // SCO7197 // SCO5260 // SCO5229 // SCO2829 // SCO2831 // SCO2830 // SCO2828 // SCO1225 // SCO2009 // SCO2011 // SCO1459 // SCO2008	GO:0000166	2.082539739	0.00820408
GO:0006635	biological_process	fatty acid beta-oxidation	10	9	24	7910	398	SCO6789 // SCO4869 // SCO6732 // SCO6027 // SCO6730 // SCO6968 // SCO3247 // SCO1838 // SCO2774	GO:0003824	2.89779986	0.00196951

Term	Definition
GOID	Gene Ontology identifier
Ontology	Category of GO identifier
Term	GOID term identification
Level	The level of the GO term, defined by the longest path connecting back to the root of the GO hierarchical tree
q	Count of genes associated with the listed GOID in the dataset
k	total number of probes in he dataset
m	count of genes associated with the listed GOID in the full gene list
t	number of genes in the full gene list
probes	genes associated with the GOID
log_odds_ratio	Log2 of the odds ratio for the enrichment of the GOID.
p	P-value of the significance for the enrichment in your dataset of the listed GOID
